# Genomic landscape of liquid biopsy mutations in *TP53* and DNA damage genes in cancer patients

**DOI:** 10.1038/s41698-024-00544-7

**Published:** 2024-02-26

**Authors:** Damien Vasseur, Ahmadreza Arbab, Fabiola Giudici, Christophe Marzac, Stefan Michiels, Marco Tagliamento, Arnaud Bayle, Cristina Smolenschi, Madona Sakkal, Mihaela Aldea, Hela Sassi, Filippo Gustavo Dall’Olio, Noémie Pata-Merci, Sophie Cotteret, Alice Fiévet, Nathalie Auger, Luc Friboulet, Francesco Facchinetti, Arthur Géraud, Santiago Ponce, Antoine Hollebecque, Benjamin Besse, Jean Baptiste Micol, Antoine Italiano, Ludovic Lacroix, Etienne Rouleau

**Affiliations:** 1 Medical Biology and Pathology Department, F-94805 Gustave Roussy, Villejuif, France; 2 AMMICa UAR3655/US23, F-94805 Gustave Roussy, Villejuif, France; 3 Oncostat U1018, Inserm, Université Paris-Saclay, Équipe Labellisée Ligue Contre le Cancer, Villejuif, France; 4 Bureau de Biostatistique et d’Épidémiologie, Gustave Roussy, Université Paris-Saclay, Villejuif, France; 5 Cancer Medicine, Gustave Roussy, Villejuif, France; 6 Drug Development Department (DITEP), Gustave Roussy, Villejuif, France; 7 Université Paris-Saclay, Institut Gustave Roussy, Inserm, Biomarqueurs prédictifs et nouvelles stratégies thérapeutiques en oncologie, Villejuif, France; 8 Department of Hematology, Gustave Roussy, Villejuif, France

**Keywords:** Cancer genomics, Metastasis

## Abstract

Next-generation sequencing (NGS) assays based on plasma cell-free DNA (cfDNA) are increasingly used for clinical trials inclusion. Their optimized limit of detection applied to a large number of genes leads to the identification of mutations not confirmed in tissue. It becomes essential to describe the characteristics and consequences of these *liquid biopsy-only mutations*. In the STING protocol (Gustave Roussy, NCT04932525), 542 patients with advanced solid cancer had cfDNA-based and tissue-based NGS analysis (performed by FoundationOne® Liquid CDx and FoundationOne CDx™, respectively). Mutations identified in the liquid biopsy but not in the paired tissue were considered as *liquid biopsy-only mutations* irrespective of their variant allelic frequency (VAF). Out of 542 patients, 281 (51.8%) harbored at least one *liquid biopsy-only mutation*. These patients were significantly older, and more heavily pretreated. *Liquid biopsy-only mutations* occurring in *TP53*, and in *DDR* genes (*ATM*, *CHEK2, ATR, BRCA2,* and *BRCA1)* accounted for 90.8% of all the mutations. The median VAF of these mutations was generally low (0.37% and 0.40% for *TP53* and *DDR* genes respectively). The variant type repartition depended on the gene. *Liquid biopsy-only mutations* affected hotspot in *TP53* codon 273, 125, 195, 176, 237 or 280 and *ATM* codon 2891 and 3008. In a subset of 37 patients, 75.0%, 53.5% and 83.3% of the *liquid biopsy-only mutations* occurring respectively in *ATM*, *TP53,* and *CHEK2* were confirmed in the matching whole blood sample. Although *liquid biopsy-only mutations* makes the interpretation of liquid biopsy results more complex, they have distinct characteristics making them more easily identifiable.

## Introduction

Being able to reflect tumor heterogeneity^1^, liquid biopsies are useful for identifying molecular targets, monitoring treatment response^2^ and identifying resistance mechanisms^3–5^. The use of liquid biopsies raises several questions regarding incidental germline alterations^6^, identification of occult malignancies^7^ or detection of hematological malignancies^8^. Additional technical advantages include low invasiveness of blood drawing, the easy repeatability of the analysis or the short turnaround time that ensures early initiation of treatment. Moreover, when tissue biopsy has failed due to low tumor cell content or the quality or quantity of material extracted, liquid biopsy can provide a molecular diagnosis.

The variety of the parameters tested with sensitivity and specificity (single nucleotide variations (SNV), fusion genes, copy number alterations, tumor mutational burden, microsatellite status, tumor fraction (TF))^9^ vouches for the clinical applicability of cfDNA technology in the field of precision oncology and immunotherapy. FDA recently approved two next-generation sequencing (NGS) large cancer-related genes panel (large CGP) on circulating cell free DNA (cfDNA): FoundationOne® Liquid CDx (Foundation Medicine, Inc; Cambridge, MA) and Guardant360® CDx (Guardant Health, Inc.; Redwood, CA, USA).

Besides it’s utilization in the clinical setting, these characteristics make liquid biopsy a useful tool for molecular tumor boards and clinical trials inclusion^9–11^ as an excellent complement to tissue biopsy^12^, still considered the gold standard. Clinical trials increasingly allow the use of liquid biopsy only as a screening tool for trial eligibility. Therefore, it will become very common to have to compare the results of a liquid biopsy with an analysis performed on an already available archival tissue sample. Nevertheless, to date, few pan-cancer studies have compared the concordance of the two matrices using large NGS panels.

In recent years, many clinical trials have focused on DNA damage response (DDR) pathways, notably through the use of poly(ADP) ribose polymerase inhibitors (PARPi) or ATR inhibiting molecules^13^; many of such trials use the presence of *DDR* alterations as a biomarker for trial eligibility. More recently, some molecules have been able to target the *TP53* pathway^14^. The *TP53* gene, because of its high frequency of somatic mutation in solid tumor, is often used as a marker to assess the presence of tumor-derived DNA in liquid biopsy. The main objective is to retain in the liquid biopsy only the variants that are also present in the tumor, i.e., *concordant mutations*. In fact, the *liquid biopsy-only mutations* should not be retained because their involvement in the solid tumor development is unclear. Some of these mutations may be clonal hematopoiesis-like while others are subclonal mutations acquired later in the cancer evolution which would question their actionability.

In the present study, we analyzed *liquid biopsy-only mutations* occurring in *TP53* or in *DDR* genes regardless of the observed variant allelic frequency (VAF) and *concordant mutations* detected in paired plasma and tissue sample analysis in order to identify specific features of *liquid biopsy-only mutations*. We also confirmed the hematopoietic origin of a subset of mutations by NGS performed on whole blood samples.

On this basis, we describe the context in which *liquid biopsy-only mutations* are found, confounding factors, specific traits related to these mutations in terms of variant type, genes involved, VAF and recurrent altered positions.

Overall, the aim of this work is to provide consolidated data for clinicians and molecular biologists to interpret the results of cfDNA large CGP and to improve the description of *liquid biopsy-only mutations* occurring in *TP53* or in *DDR* genes.

## Results

### Patients and sample characteristics

The characteristics of the 542 patients included in this study are summarized in Table 1 (and Supplementary Table S1).

**Table 1:** Patients and samples characteristics

		No *liquid biopsy-only mutation*	At least 1 *liquid biopsy-only mutation*	*p*-value	Overall
		*n* = 261 (48.2%)	*n* = 281 (51.8%)		*n* = 542 (100.0%)
Age at blood collection	Mean (SD)	56.9 (13.0)	62.8 (10.8)		60.0 (12.3)
	Median (IQR)	58 (48–66)	64 (56–71)	<0.001	61 (54–69)
	Min–Max	21–84	30–85		21–85
Gender	Male	146 (55.9%)	125 (44.5%)	0.009	271 (50.0%)
	Female	115 (44.1%)	156 (55.5%)		271 (50.0%)
Delta Tissue-Liquid (m)	Mean (SD)	18.2 (25.4)	24.2 (28.9)		21.3 (27.4)
	Median (IQR)	11 (2–25)	16 (4–34)	0.003	13 (3–28)
	Min–Max	0–189	0–218		0–218
Tumor fraction (when evaluable)^a^	Mean (SD)	31.4% (16.8)	26.4% (16.2)	0.02	29.0% (16.7)
	Median (IQR)	58(17–45)	21(14–34)		25(15–40)
	Min–Max	10–72	09–72		09–72
Blood TMB (mut/Mb)	Mean (SD)	5.9 (6.8)	9.8 (23.1)	0.005	7.7 (17.4)
	Median (IQR)	3.8 (1.3–7.6)	3.8 (2.5–8.9)		3.8 (1.3–7.6)
	Min–Max	0–45.5	0–269.4		0–269.4
Smoking	Yes	109 (41.8%)	124 (44.1%)	0.12	233 (43.0%)
	No	88 (33.7%)	108 (38.5%)		196 (36.2%)
	Unknown	64 (24.5%)	49 (17.4%)		113 (20.8%)
Prior treatment lines	Mean (SD)	1.6 (1.4)	2.1 (1.9)	<0.001	1.9 (1.7)
Inflammation (CRP > or <10 mg/L)	Yes	74 (28.4%)	102 (36.3%)	0.11	176 (32.5%)
	No	69 (26.4%)	60 (21.4%)		129 (23.8%)
	Unknown	118 (45.2%)	119 (42.3%)		237 (43.7%)
Primary tumor type	Colorectal	66 (25.3%)	37 (13.2%)	<0.001	103 (19.0%)
	Lung	44 (16.9%)	48 (17.1%)	1.0	92 (17.0%)
	Breast	28 (10.7%)	27 (9.6%)	0.77	55 (10.1%)
	Pancreas	24 (9.2%)	23 (8.2%)	0.79	47 (8.7%)
	Liver and biliary tract	15 (5.7%)	15 (5.3%)	0.98	30 (5.5%)
	Head and neck	10 (3.8%)	19 (6.8%)	0.19	29 (5.3%)
	Prostate	11 (4.2%)	16 (5.7%)	0.55	27 (5.0%)
	Other	8 (3.1%)	19 (6.8%)	0.08	27 (5.0%)
	Uterus	7 (2.7%)	19 (6.8%)	0.04	26 (4.8%)
	Stomach	14 (5.4%)	10 (3.6%)	0.42	24 (4.4%)
	Urothelial	11 (4.2%)	11 (3.9%)	1.0	22 (4.1%)
	Unknown	6 (2.3%)	14 (5.0%)	0.15	20 (3.7%)
	Ovarian	6 (2.3%)	12 (4.3%)	0.30	18 (3.3%)
	Oesophagus	10 (3.8%)	4 (1.4%)	0.14	14 (2.6%)
	Thyroid	1 (0.4%)	7 (2.5%)	0.09	8 (1.5%)

^a^Available for *n* = 93 no *liquid biopsy-only mutations* and *n* = 85 at least 1 *liquid biopsy-only mutations*

At least one *liquid biopsy-only mutation* occurring in *TP53* or in *DDR* genes was identified in 51.8% of the patients (*n* = 281/542).

Patients with at least one *liquid biopsy-only mutation* were significantly older (mean: 62.8 vs 56.9 year-old *p* < 0.001) with the proportion of patient concerned by *liquid biopsy-only mutation* increasing with age (Supplementary Fig. S1) and were female in 55.5% of cases (*n* = 156/281). Conversely, patients without any *liquid biopsy-only mutation* were more frequently male (*n* = 146/261).

The median time between blood and tissue sampling was statistically different between patients with or without *liquid biopsy-only mutations* (16 vs 11 months respectively, *p* = 0.003). Overall, more than 50% of the liquid samples were collected within a period of less than 13 months after the corresponding tissue samples (Supplementary Fig. S2).

TF was evaluable for 33.0% (*n* = 178/542) of patients with a mean of 31.4% for patients without *liquid biopsy-only mutations* and 26.4% for patients with at least one *liquid biopsy-only mutation* (*p* = 0.02). Patients with at least one *tissue biopsy-only mutation* more frequently had undetectable TF than those without (80.0% (132/165) vs 61.3% (*n* = 232/377) respectively, *p* < 0.001). Detectable TF was also strongly dependent on cancer subtype. Prostate (55.6%, *n* = 15/27), urothelial (54.5%, *n* = 12/22), esophageal (50.0%, *n* = 7/14), gastric (41.7%, *n* = 10/24) and colorectal (39.8%, *n* = 41/103) cancers were the most frequent cancers for which a TF could be estimated. Conversely, for head and neck (13.8%, *n* = 4/29), ovarian (16.7%, *n* = 3/18), breast (21.8%, *n* = 12/55), pancreatic (23.4%, *n* = 11/47) and liver and biliary tract (26.7%, *n* = 8/30) cancers, TF was less frequently evaluable. Finally, detectable TF was more common in male patients (37.6%, *n* = 102/271) than in female patients (28.0%, *n* = 76/271) (*p* = 0.02).

bTMB was higher for patients with at least one *liquid biopsy-only mutation* (9.8 mut/Mb vs 5.9 mut/Mb, *p* = 0.005) and mean average bTMB ranged from 3.51 mut/Mb for ovarian cancers to 10.2 mut/Mb for gastric cancer (Supplementary Fig. S3).

No statistical association was found between smoking status and the presence of *liquid biopsy-only mutations*. 44.1% of patients with at least one *liquid biopsy-only mutation* were smokers compared with 41.8% of patient without any *liquid biopsy-only mutations* (*p* = 0.12).

Patients with at least one *liquid biopsy-only mutation* were significantly more heavily pre treated with an average of 2.1 prior treatment lines vs 1.6 for patients without *liquid biopsy-only mutations* (*p* < 0.001).

Of the 542 patients, 305 (56.3%) had a CRP dosage at the time of liquid biopsy collection. The 176 patients with increased CRP, classically associated with inflammation, were not more likely to have *liquid biopsy-only mutations* than the others although the threshold for significance was close (*p* = 0.11).

Regardless of the presence or absence of *liquid biopsy-only mutations*, the three most prevalent tumor histologies in this cohort remained consistent: colorectal cancer, lung cancer, and breast cancer. Nevertheless, the proportion of colorectal cancer in patients without *liquid biopsy-only mutations* was much higher than in patients with at least one *liquid biopsy-only mutation* (25.3% vs 13.2% *p* < 0.001). The six most common cancers in women were breast (20.3%, *n* = 55/271), colorectal (19.2%, *n* = 52/271), lung (13.3%, *n* = 36/271), uterine (9.6%, *n* = 26/271), pancreatic (7.7%, *n* = 21/271) and ovarian (6.6%, *n* = 18/271) cancers. In men, the most common cancers were lung (20.7%, *n* = 56/271), colorectal (18.8%, *n* = 51/271), prostate (10.0%, *n* = 27/271), pancreatic (9.6%, *n* = 26/271), head and neck (8.8%, *n* = 22/271) and urothelial (6.3%, *n* = 17/271) cancers.

**Fig. 1:**
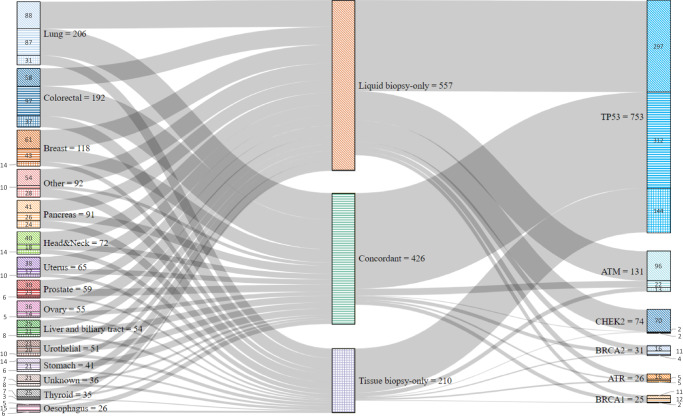
Uncovering the origin and impacted genes of the 1193 identified mutations. Sankey diagram representing the histological localization of the 1193 mutations identified in this cohort and the main genes involved. Inverted diagonal stripes represent *liquid biopsy-only mutations*, thin horizontal stripes represent *concordant mutations*, and thin horizontal cross-hatching represent *tissue biopsy-only mutations*.

### Origin of identified mutations and affected genes (Fig. 1)

A total of 1193 mutations were collected, of which 557 (46.7%, *n* = 557/1193) were *liquid biopsy-only mutations*, 426 (35.7%, *n* = 426/1193) were *concordant mutations*, and 210 (17.6%, *n* = 210/1193) were *tissue biopsy-only mutations* (Supplementary Table S2).

Over half of the mutations observed were found in the four most frequent cancers described earlier: lung (17.3%, *n* = 206/1193 mutations), colorectal (16.1%, *n* = 192/1193 mutations), breast (9.9%, *n* = 118/1193 mutations) and other cancers (7.7%, *n* = 92/1193 mutations). For all sites, with the exception of digestive cancers like colorectal, stomach and esophagus, the relative majority of the identified mutations are *liquid biopsy-only mutations*.

The six most frequently mutated genes were *TP53* (63.1%, *n* = 753/1193), *ATM* (11.0%, *n* = 131/1193), *CHEK2* (6.2%, *n* = 74/1193), *BRCA2* (2.6%, *n* = 31/1193), *ATR* (2.2%, *n* = 26/1193) and *BRCA1* (2.1%, *n* = 25/1193). *TP53*, *ATM,* and *CHEK2* alone accounted for 80.3% (958/1193) of all identified mutations and 83.1% (463/557) of all *liquid biopsy-only mutations*. For mutations identified in *TP53*, 41.4% (*n* = 312/753) were *concordant mutations*, 39.4% (n = 297/753) were *liquid biopsy-only mutations*, and 19.1% (*n* = 144/753) were *tissue biopsy-only mutations*. For *ATM*, the majority of the mutations were *liquid biopsy-only mutations* 73.3% (*n* = 96/131), 16.8% (*n* = 22/131) were *concordant mutations,* and 9.9% (*n* = 13/131) were *tissue biopsy-only mutations*. *CHEK2* had the highest proportion of *liquid biopsy-only mutations* reaching 94.6% (*n* = 70/74). Finally, for *BRCA2, ATR,* and *BRCA1*, almost half of the identified mutations were *liquid biopsy-only mutations* 51.6% (*n* = 16/31), 61.5% (*n* = 16/26), and 44% (*n* = 11/25) respectively. Whatever the localization of the primary tumor, *TP53* is the most mutated among the *liquid biopsy-only mutations* (between 38% of *liquid biopsy-only mutations* for colorectal cancers and 71% of *liquid biopsy-only mutations* for cancers of unknown origin) (Supplementary Fig. S4).

The relative proportion of *liquid biopsy-only mutations* to *concordant mutations* demonstrated an over representation of *ATM* (17.3% vs 5.2%) and *CHEK2* (12.6% vs 0.5%) whereas *MUTYH* was under represented (0.4% vs 5.2%) (Supplementary Fig. S5).

### *Liquid biopsy-only mutations* were identified with very low VAF (Supplementary Fig. S6)

**Fig. 2:**
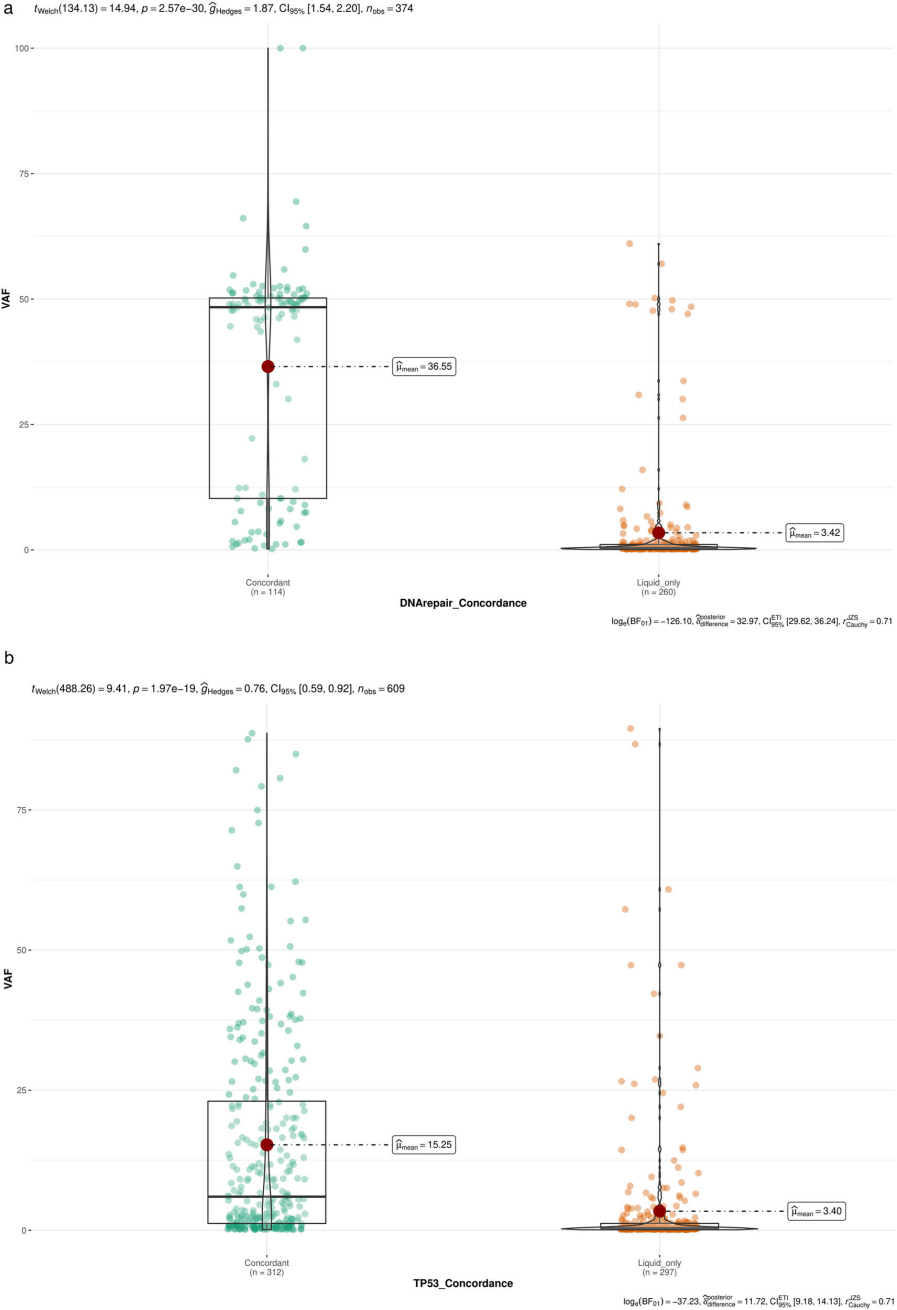
VAF comparison between *liquid biopsy-only mutations* and *concordant mutations*. **a** Boxplot representing the VAF of *concordant mutations* (light blue) and *liquid biopsy-only mutations* (light red) occurring in *DDR* genes. **b** Boxplot representing the VAF of *concordant mutations* (light blue) and *liquid biopsy-only mutations* (light red) occurring in *TP53*.

The VAF distribution of *concordant mutations* and *liquid biopsy-only mutations* was statistically different with a 100-fold higher median for *concordant mutations* considering *DDR* mutations (48.3% vs 0.4%, *p* < 0.001) (Fig. 2a).

Regarding *TP53* mutations, 75% of *liquid biopsy-only mutations* had a VAF lower than 1.27%, a frequency close to 1.22% which was the VAF of the first quartile of *concordant mutations*. The median VAF was also statistically different and significantly higher for *concordant mutations* than for *liquid biopsy-only mutations* (6.0 vs 0.4, *p* < 0.001 respectively) (Fig. 2b).

### Variant type differed between liquid biopsy-only mutations and concordant mutations

**Fig. 3:**
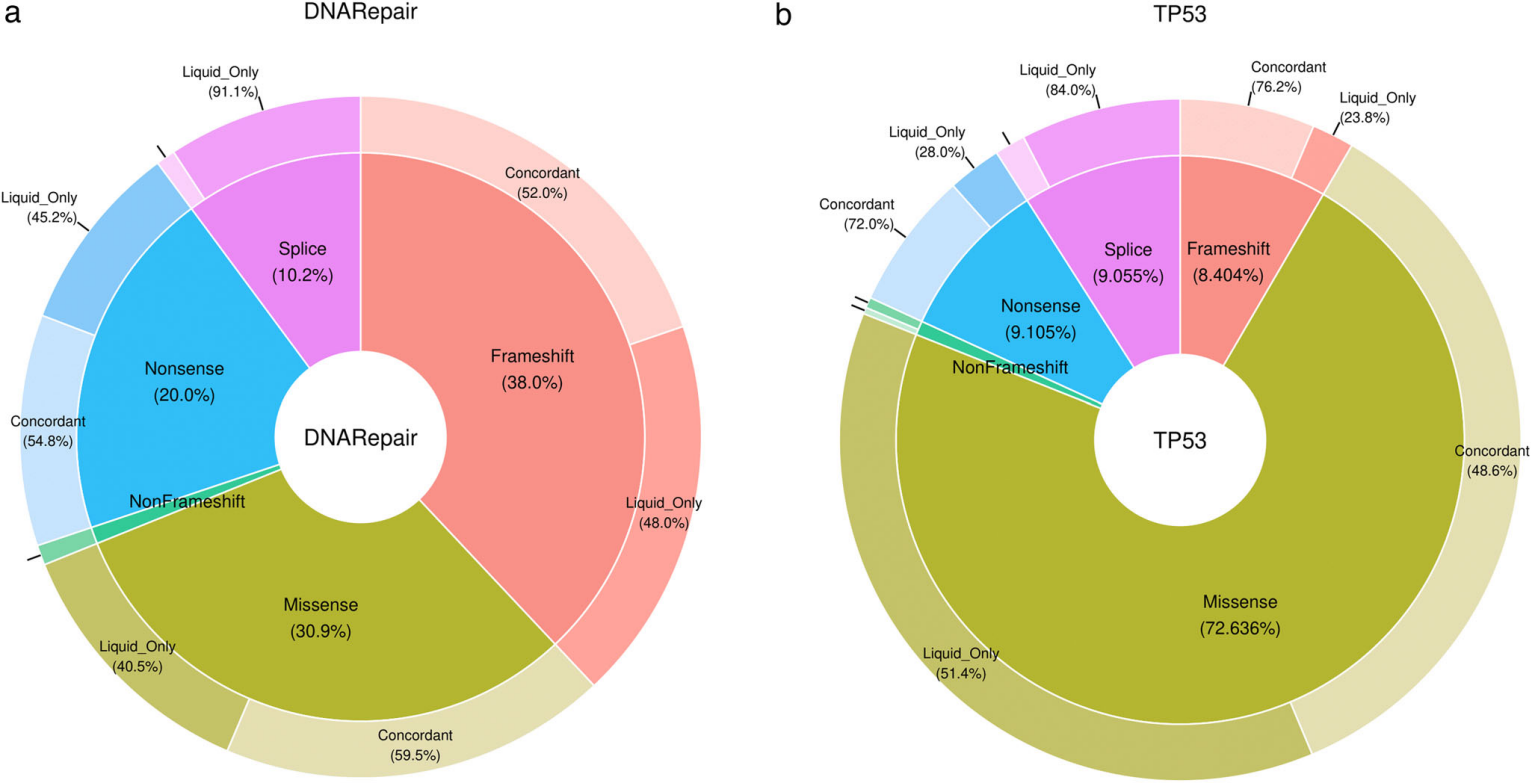
Illustrating the distinctions in variant types between *liquid biopsy-only mutations* and *concordant mutations*. Pie donut chart representing the proportions of the different variant types. Frameshift are represented in red, Missense in green, Non sense in blue, Splice site in purple and Non frameshift (insertion and deletion inframe) in dark green. **a** Repartition of variant type between *concordant mutations* and *liquid biopsy-only mutations* occurring in *DDR* genes; **b** Repartition of variant type between *concordant mutations* and *liquid biopsy-only mutations* occurring in *TP53*.

For *DDR* genes (Fig. 3a), missense mutations were more frequently *concordant mutations* than *liquid biopsy-only mutations* (59.5% vs 40.5% respectively, *p* = 0.03).

For *DDR* genes and *TP53*, splice site mutations were more frequently *liquid biopsy-only mutations* than *concordant mutations* (91.1% vs 8.9%, *p* < 0.001 and 84.0% vs 16.0%, *p* < 0.001 respectively).

Finally, for *TP53* (Fig. 3b), frameshift and nonsense mutations were more frequently *concordant mutations* than *liquid biopsy-only mutations* (76.2% vs 23.8%, p < 0.001 and 72.0% vs 28.0%, p < 0.001).

### *Liquid biopsy-only mutations* in *TP53* and *ATM* differ from classical hotspot

The positions of the recurrent *liquid biopsy-only mutations* were different from the classical hotspot.

For *concordant mutations*, the six most affected amino acids in *TP53* (NM_000546.5) were p.Arg248 (24 mutations, 7.7%), p.Arg175 (23 mutations, 7.4%), p.Arg273 (20 mutations, 6.4%), p.Arg196 (11 mutations, 3.5%), p.Arg213 and p.Gly245 (10 mutations each, 3.2%). These results, with the exception of p.Arg196, are similar to what was observed in the MSK-Impact Clinical Sequencing cohort.

**Fig. 4:**
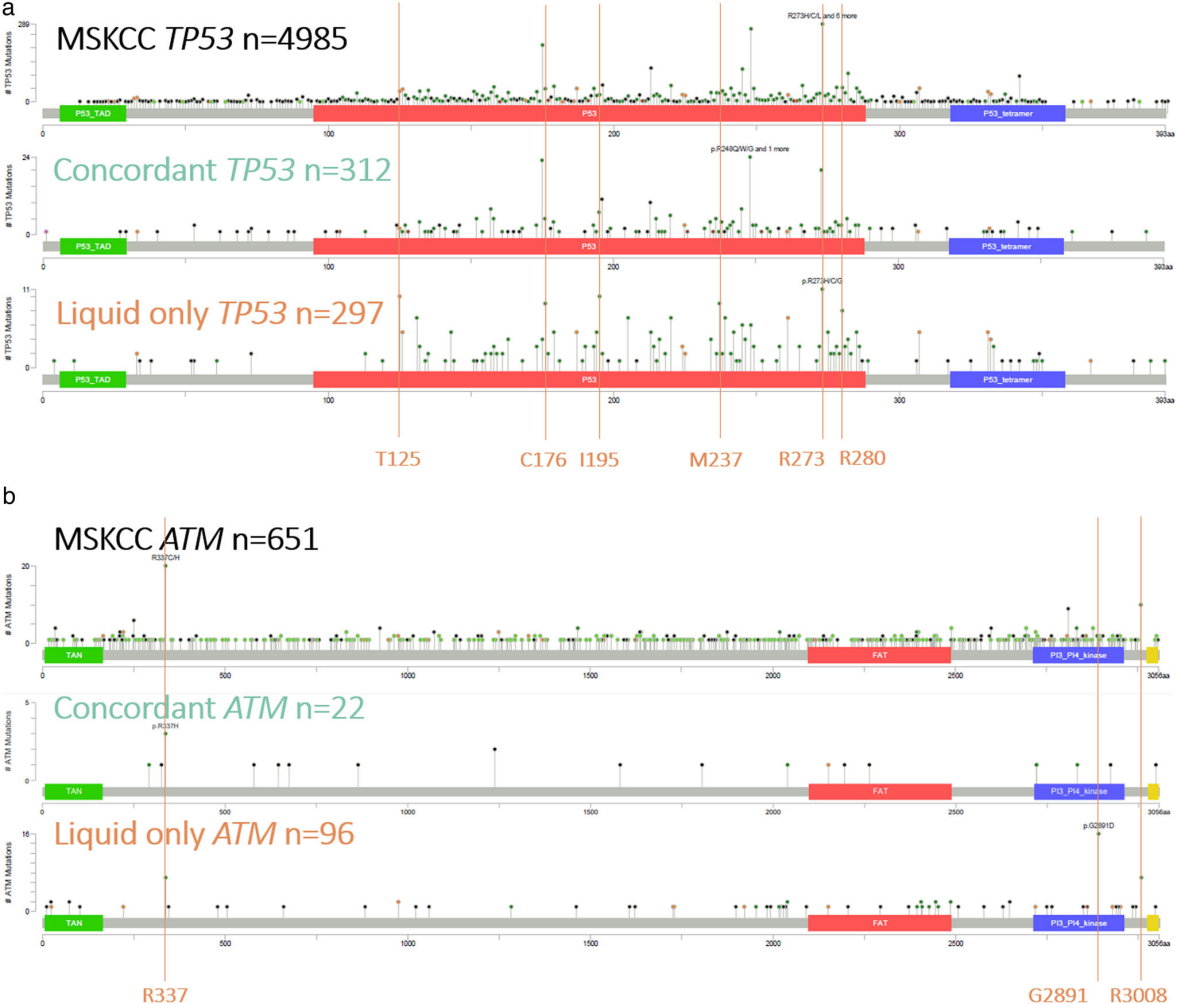
Identifying distinct mutational hotspots in *liquid biopsy-only mutations* compared to *concordant mutations*. *TP53* and *ATM* lollipop plot. The structural domains of the gene are represented by color. Missense variants are represented in green, truncating variants in black and splice variants in orange. The upper panel represents the repartition of the variants identified in the MSKCC cohort, the intermediate panel represents the repartition of the *concordant mutations* identified in our cohort and the lower panel represents the repartition of the *liquid biopsy-only mutations* identified in our cohort. **a** Lollipop plot depicting the *TP53* mutations. **b** Lollipop plot depicting the *ATM* mutations.

The six most altered positions in the 297 *TP53 liquid biopsy-only mutations* were p.Arg273 (11 mutations, 3.7%), p.Ile195 and p.Thr125 (10 mutations each, 3.4%), p.Cys176 and p.Met237 (9 mutations each, 3.0%) and p.Arg280 (8 mutations, 2.7%) (Fig. 4a).

While p.Arg273 was also commonly reported as *concordant mutations*, the residues p.Thr125, p.Ile195, p.Cys176, p.Met237 and p.Arg280 positions were rare in our *concordant mutations* group as well as in the MSK-Impact Clinical Sequencing Cohort (40 (0.8%), 27 (0.5%), 49 (1.0%), 32 (0.6%), 53 (1.1%) mutations respectively).

Similar observations can be made for *ATM* (NM_000051.3). In the 96 *liquid biopsy-only mutations* occurring in *ATM* mutations identified in this work, while positions p.Arg3008 and p.Arg337 were the second most impacted amino acid with 7 (7.3%) mutations, the most impaired amino acid was p.Gly2891 with 16 mutations (16.7%), a position described only 2 times out of 651 mutations in the MSK-Impact cohort (0.3%) compared with 20 (3.1%) for p.Arg337 and 10 (1.5%) for p.Arg3008 (Fig. 4b).

**Fig. 5:**
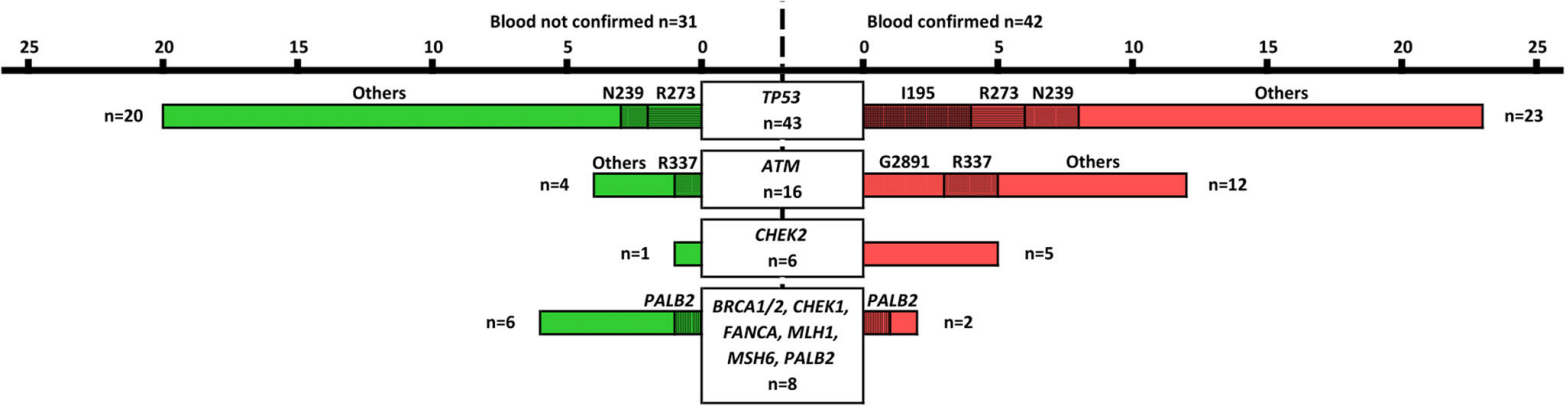
Validation of a subset of *liquid biopsy-only mutations* through whole blood NGS. Butterfly chart presenting the level of confirmation of 73 *liquid biopsy-only mutations* in the matched whole blood.

### NGS in whole blood focused on *liquid biopsy-only mutations* (Fig. 5)

A total of 37 blood samples were sequenced to confirm the hematopoietic origin of 73 *liquid biopsy-only mutations* (Supplementary Table S3). Out of these mutations, 42 (57.5%) were confirmed in the matched blood sample. Specifically, 23 out of 43 (53.5%) *TP53 liquid biopsy-only mutations* were confirmed in the matching blood, particularly those affecting the amino acid p.Ile195 that were systematically confirmed. Regarding *ATM*, *liquid biopsy-only mutations* were highly confirmed (75%, 12/16) as were mutations occurring in *CHEK2* (83.3%, 5/6). Finally, 2 out of 8 *liquid biopsy-only mutations* affecting *BRCA1*, *BRCA2*, *CHEK1*, *FANCA*, *MLH1, MSH6*, and *PALB2* were confirmed in the blood sample.

## Discussion

In our cohort, *liquid biopsy-only mutations* affecting *TP53* or *DDR* genes were relatively common events, occurring in half of the patients, making the interpretation of clinical reports particularly complex and at risk to misguide treatment selection in patients with cancer.

Although these mutations do not meet the criteria to be classified as clonal hematopoiesis (CH) in terms of VAF and genes involved, they share some common characteristics with CH. Aging seems to be a parameter that impacts the emergence of *liquid biopsy-only mutations* and patients with at least one *liquid biopsy-only mutation* were significantly older than others. The link between aging and CH has already been well documented and can be explained by the accumulation of mutations due to hematopoietic stem cell replication errors, as well as by stem cell exhaustion leading to a reduced repertoire diversity^15^. Prior lines of treatment can also significantly impact the emergence of *liquid biopsy-only mutations*, the mean number of previous lines of treatment was 2.1 in the subgroup of patients with *liquid biopsy-only mutations*, compared to 1.6 in the other group. Previous studies have also shown that mutations in *DDR* genes were strongly associated with prior exposure to cancer therapy^16^.

However, the *liquid biopsy-only mutations* identified in this cohort differed from CH in terms of lack of correlation between these mutations and the inflammatory status, although CRP is not a robust marker of chronic inflammation^17^. Inflammation is strongly associated with the development of CH mutations, being related to natural aging phenomena through inflammaging^18^. It appears that dysregulation of inflammation processes contributes to clonal expansion through various cytokines (IL6, TNF-α,…). Interestingly, the CH mutants also acts on inflammation in a self-perpetuating cycle of inflammation and expansion^19^.

*Liquid biopsy-only mutations* identified in this work were more commonly found in women than in men because of the different cancer subtypes and their respective TF. Women were predominantly affected by breast cancer, a subtype for which TF was evaluable in only 21.8% of the cases. In contrast, prostate cancer for which TF was evaluable in 55.6% of the cases, was one of the most common tumor in men.

bTMB was more elevated in the samples with at least one *liquid biopsy-only mutation*. As these mutations were mostly not known in polymorphism databases like dbSNP or ExAc nor classical oncogenic drive databases, they enter in the calculation of the TMB increasing it artificially.

*Liquid biopsy-only mutations* appear to be rarer in patients with colorectal cancers. While the differences in age and tumor fraction between colorectal cancer patients and those with other cancers are not statistically significant, certain trends can be observed. Specifically, colorectal cancer patients tend to be younger (58.1 years old vs 60.4 years old respectively *p* = 0.1). In addition, a higher proportion of colorectal cancer patients had evaluable tumor fractions compared to other types of cancer (*n* = 41/103 (39.8%) vs *n* = 137/439 (31.2%) respectively *p* = 0.12). These observations are consistent with what reported in previous studies, demonstrating that colorectal cancers were among the cancers with the highest tumor shedding^20^. Moreover, colon cancer is one of the few solid tumors for which no increased risk of myelodysplastic syndrome or acute myeloid leukemia has been observed after chemotherapy^21^.

*Liquid biopsy-only mutations* have intrinsic characteristics in terms of affected genes, variant type, impacted amino acid and VAF.

Unsurprisingly, in this cohort of metastatic cancer patients, and with the large CGP used, *TP53* emerged as the most frequently mutated gene, accounting for 63.1% of all identified mutations. Furthermore, 39.4% of the mutations observed in *TP53* were *liquid biopsy-only mutations* without easy determination of their origin as either hematopoietic or solid tumor related. A much higher proportion of *liquid biopsy-only mutations* can be observed for *ATM* and for *CHEK2* (73.3% and 94.6% respectively), the second and third most mutated genes in our cohort. The relative proportion of *liquid biopsy-only mutations* to *concordant mutations* was high because of their likely linkage to CH. Conversely, the relative proportion of *MUTYH* was low because of the likely germline origin of the mutations occurring in this gene. Interestingly, we were able to confirm the hematopoietic origin of 53.5% (23/43 mutations), 75.0% (12/16 mutations) and 83.3% (5/6 mutations) of the *liquid biopsy-only mutations* tested occurring in *TP53*, *ATM* and *CHEK2* respectively. The *ATM* gene has been described as a key element in the reconstitution of HSC capacity, a non-functional gene leading to increased reactive oxygen species and bone narrow failure^22^. More broadly, 46.7% of the mutations occurring in the *DDR* genes and *TP53* were *liquid biopsy-only mutations* and we were able to confirm a subset of these in blood in 57.5% (42/73 mutations) of cases. This point is crucial, since deleterious variants in genes included in the *DDR* pathway are increasingly part of inclusion criteria for clinical trials evaluating PARPi^23^. Validating the inclusion on the sole basis of liquid biopsy result may lead to lack of efficacy and to the risk of not giving the patient the best possible chance^24^.

Another specificity of *liquid biopsy-only mutations* was the distribution of variant types. In our cohort, splice site mutations were found almost exclusively in the *liquid biopsy-only mutations* subgroup. 84.0% and 91.1% respectively of splice sites occurring in *TP53* and in *DDR* genes were *liquid biopsy-only mutations*. For *TP53*, frameshift and nonsense mutations were more frequent in the *concordant mutations* and for *DDR* genes, missenses mutations were more frequent for *concordant mutations*.

*Liquid biopsy-only mutations* were identified at lower frequencies than *concordant mutations*. More than 75% of the *liquid biopsy-only mutations* were found with a VAF < 1.27% for *TP53* and <1.04% for *DDR* genes. With the decrease in LoD of the new cfDNA panels, these mutations were more frequently reported in clinical reports and need to be interpreted. While for *TP53* the median VAF of *concordant mutations* was 6.0%, the median VAF for *DDR* genes reached 48.3%. It is very likely that most of these *concordant mutations* were in fact germline mutations so that the level of confirmation of somatic mutations occurring in *DDR* genes was probably very low.

Last specificity of *liquid biopsy-only mutations* was the impacted amino acid. In the top six most altered amino acids of *TP53*, five atypical position were identified p.Ile195, p.Thr125, p.Cys176, p.Met237, and p.Arg280, the other being p.Arg273 an already known hotspot position^25^. Interestingly the amino acid p.Ile195 located in the DNA-binding domain was described as one of the most affected positions in an acute lymphoblastic leukemia cohort as well as the p.Arg273 position^26^. The same observation can be made for *ATM*, a gene for which the most altered position was p.Gly2891 in our cohort and not p.Arg337 or p.Arg3008 as in the MSK-Impact Clinical Sequencing Cohort^27^. This amino acid located in the kinase domain has been described in CH clones in the context of prostate cancer tested for PARPi eligibility^24^ but also in a cohort of T-cell prolymphocytic leukemia confirming its involvement in hematology^28^.

The gap time between liquid and tissue biopsy may be considered as an important limitation of this retrospective study. It is known that the proportion of patients with cancer related alterations increase with the time elapsed between tissue and ctDNA profiling^29^. Nevertheless, this condition is commonly encountered in real life scenarios as it is rare for two analyses to be performed synchronously in a diagnostic context.

In terms of confirmation rates, it is possible that certain *liquid biopsy-only mutations* may be missed in the tissue biopsy due to sampling bias, preventing the assessment of the tumor’s spatial and temporal heterogeneity. As a result, a tissue profile may falsely show a negative result for an alteration identified in the liquid biopsy. On the other hand, liquid biopsy is recognized as a reliable reflection of tumor heterogeneity and serves as a valuable tool for detecting second occult solid cancers^7^ or hematological malignancies^8^.

Lastly, as all cfDNA analyses were performed using a single NGS technique, it cannot be excluded that *liquid biopsy-only mutations* identified at very low VAF were NGS artifacts, particularly for frameshifts as we did not have access to the raw sequencing data and the low limit of detection. However, this hypothesis seems unlikely as tissue and liquid NGS were performed using highly validated and controlled FDA-approved techniques that are supposed to ensure highly reliable results.

Overall, mutations identified in liquid biopsies using large CGP and occurring in *TP53* or in *DDR* genes should be interpreted very carefully especially if they are identified at very low VAF, if the variant class is a splice site or if it affected a specific position previously described (Supplementary Fig. S7). While these mutations may not precisely align with the definition of CH, their confirmation through the NGS technique in paired blood samples suggests an improbable association with solid tumors. Consequently, their potential usefulness for clinical trials is limited. However, if there is any uncertainty regarding the origin of a mutation identified in a liquid biopsy within a tumor-informed context, it is essential to reference the previously conducted tissue analysis. If the mutation was not detected in the initial tissue analysis and the liquid biopsy was performed during progression, it is possible that this mutation is implicated in disease progression. In such cases, or in a tumor-uninformed context, it is advisable to conduct a tissue analysis if feasible. Tissue analysis remains the gold standard technique for confirmation and serves as the reference method for determining the origin of mutations.

## Methods

### Patients

Between December 2020 and July 2022, a total of 542 patients with advanced cancer were included in the STING protocol (Gustave Roussy Cancer Profiling, NCT04932525). This protocol was approved by the CPP (“Comité de protection des personnes”) and by the ANSM (“Agence nationale de sécurité du médicament et des produits de santé”). Written informed consent was obtained from all patients included in this study. This work was conducted in accordance with the Declaration of Helsinki.

Each patient had matched molecular profiles based on liquid and tissue biopsies. Each individual genomic report was reviewed and discussed within a multidisciplinary tumor board to define the presence or absence of actionable therapeutics targets, based on ESCAT classification^30^.

### Molecular analyses

Liquid biopsy analysis was performed on plasma samples collected when looking for a new actionable target using the FoundationOne® Liquid CDx (Foundation Medicine, Inc; Cambridge, MA) assay covering 324 genes and offering a limit of detection (LoD) as low as 0.1% VAF for SNVs^16^. Tissue biopsies were analyzed with the FoundationOne CDx™ (Foundation Medicine, Inc; Cambridge, MA) assay on formalin-fixed paraffin-embedded archival material of primary tumor or tumor metastasis containing at least 30% of tumor cells. The term “mutation” is used here for deleterious and probably deleterious variants.

Only SNV and insertions/deletions classified as pathogenic and present in the clinical report generated by Foundation Medicine were retained for this study. Mutations concordant between tissue and liquid biopsy were defined as *concordant mutations*, mutations identified only in the liquid biopsy were classified as *liquid biopsy-only mutations* and mutations identified only in the tissue biopsy were classified as *tissue biopsy-only mutations*.

TF was provided in the liquid biopsy clinical report and estimated based on a normalized coverage level across the genome.

Blood TMB (bTMB) also, was provided in the clinical report. It was calculated based on all the mutations identified with a VAF > 0.5%. Germline polymorphisms known in databases and oncogenic drivers were eliminated.

For the purpose of this study, the retained genes were the following:

- *TP53*, one of the most frequently mutated genes in cancer^31^, is also frequently mutated in clonal hematopoiesis^32^. For this reason, the simple identification of a *TP53* variant in a liquid biopsy will not distinguish a contributory liquid biopsy from a non-contributory one;
- a gene involved in a *DDR* pathway including base excision repair, mismatch excision repair, homologous recombination, DNA polymerases or chromatin remodeling^33^: *ATM*, *ATR*, *ATRX*, *BARD1*, *BRCA1*, *BRCA2*, *BRIP1*, *CDK12*, *CHEK1*, *CHEK2*, *ERCC4*, *FANCA*, *FANCC*, *FANCG*, *FANCL*, *MLH1*, *MRE11A*, *MSH2*, *MSH3*, *MSH6*, *MUTYH*, *NBN*, *PALB2*, *PARP1, PARP2, PARP3, PMS2, POLD1*, *POLE*, *RAD21*, *RAD51*, *RAD51B*, *RAD51C, RAD51D, RAD52*, and *RAD54L*. These mutations can be particularly challenging, especially if the liquid biopsy was performed for the purpose of enrolling the patient in a clinical trial recruiting for these alterations^24^.

The confirmation of the hematopoietic origin of certain *liquid biopsy-only mutations* was conducted using whole blood samples collected in EDTA tubes (BD Vacutainer, Beckton Dickinson and Company). Following a centrifugation step at 1000 × *g* for 10 min, the supernatant was removed, and DNA extraction from the whole blood was performed using the Maxwell® RSC Whole Blood DNA Kit (Promega, Charbonnières-les-Bains, France). Two targeted NGS panels were used. Panel 1 involved the use of SureSelectXTHS (Agilent) target enrichment with custom capture, while Panel 2 was the Oncomine Comprehensive Assay v3 (ThermoFisher). The mean (m) and standard deviation (sd) of the background noise of each mutation were calculated. The mutation was considered confirmed if the VAF was >m + 2*sd.

### C-reactive protein (CRP)

To assess the level of inflammation, CRP determination was performed in an accredited laboratory using the Atellica® CH C-Reactive Protein assay for in vitro diagnostic (Siemens Healthineers, Saint-Denis, France). The reference range for CRP concentration for adults is <10 mg/L (<1.0 mg/dL). Only CRP dosages performed within 30 days before or after liquid biopsy collection were included.

### Statistical analyses

Descriptive quantitative data were expressed as medians with interquartile ranges (IQRs) or mean with standard deviation (SD) for continuous variables according to data distribution (verified through Shapiro–Wilk test of normality); qualitative data were expressed as absolute frequencies and percentages. Mann Whitney test was performed to compare continuous variables. Pearson Chi-2 test (or Fisher exact test whenever appropriate) was used to analyze categorical variables. Statistical significance was set at *p* < 0.05.

### Reporting summary

Further information on research design is available in the Nature Research Reporting Summary linked to this article.

### Supplementary information

Reporting Summary

Supplementary information

## Data Availability

The data that support the findings of this study are available from the corresponding author upon reasonable request.

## Code availability

Sankey diagram was created using the function *sankeyNetwork* available in the networkD3 package (version 0.4). Boxplot and pie-donut chart were created using the functions *ggstatsplot* (version 0.9.5) and *webr* (version 0.1.5) available in the ggplot2,RRID:SCR_014601 package (version 3.3.5) of R statistical software. Lollipop plot were built using cBioPortal^34,35^. The frequencies of mutations identified in the MSK-Impact Clinical Sequencing cohort were obtained from cBioPortal. All statistical analyses were carried out in R software for statistical computing (Version 4.0.4; R development Core Team, 2020).

## References

[1] Russano M (2020). Liquid biopsy and tumor heterogeneity in metastatic solid tumors: the potentiality of blood samples. J. Exp. Clin. Cancer Res..

[2] Martins I (2021). Liquid biopsies: applications for cancer diagnosis and monitoring. Genes.

[3] Parikh AR (2019). Liquid versus tissue biopsy for detecting acquired resistance and tumor heterogeneity in gastrointestinal cancers. Nat. Med..

[4] Chabon JJ (2016). Circulating tumour DNA profiling reveals heterogeneity of EGFR inhibitor resistance mechanisms in lung cancer patients. Nat. Commun..

[5] Siravegna G (2015). Clonal evolution and resistance to EGFR blockade in the blood of colorectal cancer patients. Nat. Med..

[6] Stout LA (2021). Identification of germline cancer predisposition variants during clinical ctDNA testing. Sci. Rep..

[7] Aldea M (2021). Detection of additional occult malignancy through profiling of ctDNA in late-stage cancer patients. Ann. Oncol..

[8] Aldea M (2023). Liquid biopsies for circulating tumor DNA detection may reveal occult hematologic malignancies in patients with solid tumors. JCO Precis Oncol..

[9] Vasseur D (2022). Next-generation sequencing on circulating tumor DNA in advanced solid cancer: Swiss army knife for the molecular tumor board? A review of the literature focused on FDA approved test. Cells.

[10] Gray JE (2020). Clinical performance of a comprehensive novel liquid biopsy test for identifying non-small cell lung cancer (NSCLC) patients for treatment with osimertinib. J. Clin. Oncol..

[11] Jovelet C (2016). Circulating cell-free tumor DNA analysis of 50 genes by next-generation sequencing in the prospective MOSCATO trial. Clin. Cancer Res..

[12] Aggarwal C (2019). Clinical implications of plasma-based genotyping with the delivery of personalized therapy in metastatic non–small cell lung cancer. JAMA Oncol..

[13] Fizazi K (2023). Rucaparib or physician’s choice in metastatic prostate cancer. N. Engl. J. Med..

[14] Dumbrava EE (2022). First-in-human study of PC14586, a small molecule structural corrector of Y220C mutant p53, in patients with advanced solid tumors harboring a *TP53* Y220C mutation. J. Clin. Oncol..

[15] Heuser M, Thol F, Ganser A (2016). Clonal hematopoiesis of indeterminate potential. Dtsch Arzteblatt Int..

[16] Bolton KL (2020). Cancer therapy shapes the fitness landscape of clonal hematopoiesis. Nat. Genet..

[17] Sproston NR, Ashworth JJ (2018). Role of C-reactive protein at sites of inflammation and infection. Front. Immunol..

[18] Franceschi C (2006). Inflamm-aging: an evolutionary perspective on immunosenescence. Ann. N. Y. Acad. Sci..

[19] Cook EK, Luo M, Rauh MJ (2020). Clonal hematopoiesis and inflammation: Partners in leukemogenesis and comorbidity. Exp. Hematol..

[20] Bettegowda, C. et al. Detection of circulating tumor DNA in early- and late-stage human malignancies. *Sci. Transl. Med*. https://www.science.org/doi/10.1126/scitranslmed.3007094 (2014). 10.1126/scitranslmed.3007094 PMC4017867 24553385

[21] Morton LM (2019). Association of chemotherapy for solid tumors with development of therapy-related myelodysplastic syndrome or acute myeloid leukemia in the modern era. JAMA Oncol..

[22] Ito K (2004). Regulation of oxidative stress by ATM is required for self-renewal of haematopoietic stem cells. Nature.

[23] Abida W (2020). Non-BRCA DNA damage repair gene alterations and response to the PARP inhibitor rucaparib in metastatic castration-resistant prostate cancer: analysis from the phase II TRITON2 study. Clin. Cancer Res..

[24] Jensen K (2021). Association of clonal hematopoiesis in DNA Repair genes with prostate cancer plasma cell-free DNA testing interference. JAMA Oncol..

[25] Chen S (2019). Mutant p53 drives clonal hematopoiesis through modulating epigenetic pathway. Nat. Commun..

[26] Stengel A (2014). TP53 mutations occur in 15.7% of ALL and are associated with MYC-rearrangement, low hypodiploidy, and a poor prognosis. Blood.

[27] Zehir A (2017). Mutational landscape of metastatic cancer revealed from prospective clinical sequencing of 10,000 patients. Nat. Med..

[28] Kiel MJ (2014). Integrated genomic sequencing reveals mutational landscape of T-cell prolymphocytic leukemia. Blood.

[29] Bayle, A. et al. Liquid versus tissue biopsy for detecting actionable alterations according to ESCAT in patients with advanced cancer: a study from the French National Center for Precision Medicine (PRISM). *Ann Oncol*. S0923753422041473 (2022). 10.1016/j.annonc.2022.08.089 36122799

[30] Mateo J (2018). A framework to rank genomic alterations as targets for cancer precision medicine: the ESMO Scale for Clinical Actionability of molecular Targets (ESCAT). Ann. Oncol. J. Eur. Soc. Med. Oncol..

[31] Olivier M, Hollstein M, Hainaut P (2010). TP53 mutations in human cancers: origins, consequences, and clinical use. Cold Spring Harb. Perspect. Biol..

[32] Miller PG, Steensma DP (2020). Implications of clonal hematopoiesis for precision oncology. JCO Precis Oncol..

[33] Knijnenburg TA (2018). Genomic and molecular landscape of DNA damage repair deficiency across The Cancer Genome Atlas. Cell Rep..

[34] Gao J (2013). Integrative analysis of complex cancer genomics and clinical profiles using the cBioPortal. Sci. Signal..

[35] Cerami E (2012). The cBio Cancer Genomics Portal: An Open Platform for Exploring Multidimensional Cancer Genomics Data. Cancer Discov..

