# Supplementary material for: Genomic landscape of liquid biopsy mutations in TP53 and DNA damage genes in cancer patients
Source: NPJ Precis Oncol. 2024 Feb 26;8:51. doi: 10.1038/s41698-024-00544-7 (PMC10897416; doi:10.1038/s41698-024-00544-7)
Supplement: Supplementary file 2 — Supplementary information [file 41698_2024_544_MOESM2_ESM.pdf]

Supplementary Table S1

Patient characteristics and general parameters of the liquid biopsy

ID	Sex	Date of birth	Localisation	Number treatment lines	Collection date	Age (y)	Tobacco	Inflammation	Delta liquid-tissue (m)	Tumor Fraction liquid (%)	Blood TMB (mut/Mb)	Liquid biopsy-only mutation	Tissue biopsy-only mutation
12	M	08/08/63	Unknown	5	03/31/21	57	YES	YES	29	49	11.38	YES	NO
14	M	05/16/65	Pancreas	0	02/25/21	55	NO	YES	0	20	1.26	NO	NO
16	M	01/15/58	Pancreas	2	02/09/21	63	NO	NS	1	0	0	NO	YES
17	M	06/10/61	Pancreas	2	03/04/21	59	YES	NS	15	0	1.26	NO	NO
22	M	01/29/56	Colorectal	3	04/02/21	65	YES	NO	29	27	13.91	NO	NO
23	M	01/28/61	Liver and biliary tract	1	03/16/21	60	NS	NO	8	0	2.53	NO	NO
28	F	01/27/50	Colorectal	4	04/07/21	71	YES	YES	22	0	5.06	YES	NO
29	F	09/06/39	Uterus	2	03/18/21	81	NS	NO	34	0	5.06	YES	NO
30	F	06/15/50	Ovary	3	03/31/21	70	NS	NS	79	0	6.32	NO	NO
32	F	05/29/59	Uterus	1	03/18/22	62	NS	YES	25	0	5.06	NO	NO
33	M	08/03/55	Other	1	04/08/21	65	YES	NS	48	0	1.26	YES	NO
35	M	07/30/58	Head&Neck	0	04/02/21	62	YES	NO	1	0	2.53	NO	YES
36	M	06/14/46	Head&Neck	0	04/20/21	74	YES	YES	3	0	3.79	YES	YES
38	F	04/01/46	Lung	0	05/07/21	75	YES	NS	6	0	5.06	YES	NO
39	M	11/14/70	Lung	0	04/02/21	50	YES	YES	0	0	32.87	YES	YES
41	M	03/08/42	Lung	0	04/02/21	79	YES	NS	17	0	6.32	YES	NO
50	F	06/21/71	Breast	1	02/09/21	49	NS	NO	18	0	3.79	NO	YES
57	M	12/17/54	Prostate	5	04/01/21	66	YES	NS	58	40	160.58	YES	YES
60	M	06/10/50	Prostate	3	04/01/21	70	YES	NS	125	0	7.59	YES	NO
79	M	11/25/43	Prostate	4	04/15/21	77	NS	YES	157	0	3.79	NO	NO
80	M	12/29/70	Stomach	2	04/08/21	50	NO	NS	0	23	5.06	YES	NO
86	F	06/09/66	Stomach	5	04/08/21	54	NO	NO	29	14	13.91	YES	NO
87	M	03/05/47	Pancreas	2	04/08/21	74	NS	NS	0	0	2.53	YES	YES

89	M	10/17/44	Pancreas	0	04/08/21	76	YES	YES	1	0	12.64	YES	NO
90	M	09/13/61	Pancreas	0	04/08/21	59	YES	NS	16	0	2.53	YES	YES
93	F	06/03/46	Colorectal	5	04/09/21	74	NO	YES	25	0	34.14	YES	NO
96	M	04/11/48	Lung	2	04/09/21	72	YES	NS	7	16	8.85	NO	NO
97	F	01/06/43	Colorectal	1	04/09/21	78	NO	YES	42	0	2.53	NO	YES
99	M	11/03/43	Head&Neck	1	04/12/21	77	NS	NS	75	9	2.53	YES	NO
101	M	09/19/42	Urothelial	0	04/09/21	78	NO	NS	4	0	0.0	YES	YES
114	M	07/11/41	Head&Neck	1	04/14/21	79	NO	NS	13	0	3.79	YES	NO
118	F	11/21/60	Uterus	1	04/15/21	60	YES	NO	9	0	3.79	YES	YES
120	F	07/07/81	Breast	1	04/14/21	39	NS	NS	24	0	2.53	NO	NO
124	F	05/06/55	Head&Neck	4	03/16/21	65	NS	YES	47	0	3.79	YES	YES
127	F	07/10/64	Uterus	1	02/09/21	56	NO	NO	100	10	7.59	YES	NO
135	F	07/23/66	Other	2	03/26/21	54	YES	NO	3	0	2.53	YES	NO
138	F	06/21/66	Pancreas	3	02/25/21	54	NO	NO	1	0	1.26	NO	YES
140	F	07/02/52	Uterus	4	03/23/21	68	NS	NS	82	0	0.0	YES	NO
141	F	02/13/63	Ovary	2	09/03/21	58	NO	NS	81	35	8.85	NO	NO
142	M	04/20/57	Lung	2	04/20/21	64	YES	NO	2	64	12.64	NO	NO
144	F	01/30/52	Uterus	0	03/05/21	69	NO	YES	9	0	1.26	NO	NO
145	F	12/23/66	Lung	3	04/15/21	54	NS	YES	33	0	5.06	YES	NO
147	M	04/09/50	Pancreas	2	01/15/21	70	NS	YES	23	15	6.32	YES	NO
151	M	06/30/66	Lung	0	03/12/21	54	YES	YES	0	15	7.59	NO	NO
153	M	12/18/65	Lung	0	03/22/21	55	YES	NO	0	0	2.53	YES	NO
155	F	04/29/82	Breast	6	05/21/21	39	YES	NS	0	0	5.06	YES	YES
160	M	06/26/91	Head&Neck	2	04/15/21	29	NO	NS	16	0	1.26	NO	NO
161	F	09/07/77	Oesophagus	1	04/16/21	43	NO	NO	6	20	6.32	NO	NO
167	M	07/12/53	Lung	1	04/16/21	67	YES	NS	76	0	3.79	NO	YES
173	M	05/25/52	Other	1	11/25/21	69	NO	NS	46	0	1.26	YES	NO
178	F	06/30/66	Liver and biliary tract	3	04/29/21	54	NS	NS	0	15	10.12	NO	NO

183	M	07/20/49	Stomach	4	02/09/21	71	NO	NO	95	0	74.6	YES	YES
184	M	06/19/48	Pancreas	1	02/01/21	72	YES	NS	9	11	7.59	YES	YES
185	F	01/12/48	Oesophagus	1	01/12/21	73	YES	NO	17	0	0	NO	YES
186	F	04/21/58	Unknown	3	01/19/21	62	NO	NO	22	0	0.0	YES	YES
187	M	02/27/66	Unknown	3	01/13/21	54	NO	NO	27	0	3.79	YES	NO
189	M	11/15/49	Oesophagus	2	02/05/21	71	YES	YES	18	0	5.06	NO	NO
190	F	04/16/70	Uterus	2	01/26/21	50	NS	YES	7	14	56.9	YES	NO
191	M	01/19/54	Unknown	1	02/10/21	67	YES	NO	18	0	11.38	YES	NO
193	F	04/30/70	Other	2	01/25/21	50	NS	NO	37	17	8.85	NO	NO
195	M	11/09/74	Other	1	02/01/21	46	NO	NO	41	0	0.0	YES	YES
200	F	09/08/48	Liver and biliary tract	2	01/29/21	72	NS	YES	9	0	2.53	YES	NO
201	F	12/10/53	Ovary	2	02/25/21	67	NO	NS	24	0	1.26	YES	NO
202	F	06/21/81	Breast	6	01/21/21	39	NO	NO	3	16	6.32	YES	NO
203	F	09/30/66	Breast	4	01/27/21	54	NO	YES	25	0	5.06	NO	NO
206	M	09/08/65	Liver and biliary tract	4	01/20/21	55	NO	NO	22	15	53.1	YES	YES
213	M	09/27/69	Liver and biliary tract	2	01/26/21	51	YES	YES	2	0	2.53	YES	NO
214	F	02/19/58	Ovary	2	01/22/21	62	NO	NS	51	0	7.59	YES	NO
215	M	04/30/57	Unknown	2	03/11/21	63	YES	YES	3	0	5.06	NO	NO
216	M	12/29/57	Stomach	1	12/22/20	62	YES	NO	0	51	0.0	NO	NO
218	F	05/03/96	Unknown	2	03/29/21	24	NO	NS	1	0	2.53	NO	YES
219	F	02/12/72	Pancreas	3	01/26/21	48	NS	YES	8	22	3.79	NO	NO
220	F	06/19/72	Breast	1	02/23/21	48	NS	NS	24	0	3.79	NO	NO
221	M	08/04/66	Stomach	1	02/24/21	54	NS	YES	7	51	22.76	NO	NO
223	F	02/01/45	Breast	1	03/30/21	76	NO	NS	22	0	5.06	NO	NO
227	M	12/21/66	Unknown	0	02/10/21	54	NO	NS	1	0	5.06	NO	NO
228	F	04/15/61	Lung	3	01/26/21	59	NO	YES	10	0	7.59	YES	NO
229	M	04/17/66	Unknown	0	01/20/21	54	NO	YES	23	21	8.85	YES	NO
230	M	11/21/55	Unknown	2	02/16/21	65	YES	YES	13	0	1.26	NO	NO

232	M	01/13/66	Unknown	1	02/01/21	55	YES	NS	23	0	11.38	YES	YES
233	F	06/15/51	Ovary	6	02/05/21	69	YES	NO	65	0	3.79	YES	NO
236	M	10/17/59	Unknown	0	02/15/21	61	YES	YES	6	21	8.85	YES	NO
237	F	09/24/63	Breast	5	02/16/21	57	NO	NO	27	0	3.79	NO	NO
239	M	03/26/53	Head&Neck	2	02/08/21	67	YES	NO	4	0	18.97	NO	NO
240	M	03/10/58	Unknown	1	02/23/21	62	YES	NS	34	0	0.0	YES	NO
242	M	08/02/45	Colorectal	5	12/17/20	75	NO	NO	95	0	18.97	YES	YES
245	M	04/15/75	Lung	1	03/08/21	45	YES	NO	5	0	7.59	NO	NO
246	M	11/18/52	Stomach	4	01/28/21	68	YES	NO	26	11	16.44	NO	NO
247	M	02/22/64	Urothelial	3	03/31/21	57	YES	YES	6	13	11.38	NO	NO
249	M	11/01/49	Liver and biliary tract	2	01/28/21	71	YES	NS	7	31	2.53	NO	NO
253	M	10/26/64	Lung	0	01/21/21	56	YES	YES	10	0	3.79	NO	YES
254	M	04/06/54	Lung	0	02/11/21	66	YES	YES	1	0	1.26	YES	NO
256	F	06/05/48	Pancreas	0	02/02/21	72	NO	NS	5	0	6.32	YES	YES
257	M	12/26/94	Colorectal	2	01/20/21	26	NS	NS	8	0	1.26	NO	NO
259	M	03/11/71	Colorectal	0	01/20/21	49	NO	YES	8	0	1.26	YES	YES
262	F	09/22/48	Colorectal	3	02/04/21	72	NO	NS	25	25	2.53	YES	YES
264	F	08/21/62	Ovary	3	03/30/21	58	NO	NO	17	0	1.26	YES	YES
265	M	02/17/52	Pancreas	3	01/11/21	68	YES	YES	0	37	7.59	YES	NO
266	F	07/25/65	Oesophagus	2	01/20/21	55	YES	NS	10	25	11.38	YES	YES
269	F	10/30/65	Breast	2	02/03/21	55	NO	NO	7	0	0	NO	NO
271	F	07/15/72	Breast	0	02/02/21	48	NS	NO	4	0	2.53	NO	YES
272	M	08/01/43	Colorectal	2	01/14/21	77	NO	YES	78	0	7.59	NO	NO
273	M	10/16/54	Colorectal	4	02/01/21	66	YES	YES	29	72	15.17	NO	NO
276	F	01/02/62	Lung	3	02/17/21	59	NO	NO	3	0	3.79	YES	NO
280	F	07/09/70	Colorectal	3	01/28/21	50	YES	NS	21	0	1.26	YES	NO
283	M	09/17/65	Head&Neck	7	03/30/21	55	NO	YES	3	0	1.26	YES	NO
284	M	07/24/61	Colorectal	3	03/02/21	59	YES	NO	21	0	0.0	NO	NO

286	M	07/22/54	Lung	3	01/20/21	66	YES	NO	14	0	8.85	NO	NO
287	M	08/23/79	Lung	2	01/13/21	41	YES	YES	1	25	16.44	NO	NO
288	M	09/11/60	Colorectal	4	01/12/21	60	YES	YES	38	49	21.49	NO	NO
289	F	07/30/51	Uterus	4	01/26/21	69	YES	YES	33	15	6.32	YES	YES
292	M	06/09/55	Colorectal	4	01/28/21	65	NS	NS	37	0	1.26	NO	NO
293	M	01/01/39	Lung	0	02/19/21	82	YES	YES	0	0	6.32	NO	YES
294	M	10/29/71	Lung	0	03/10/21	49	YES	NS	1	0	0.0	NO	YES
295	M	05/26/69	Lung	2	02/11/21	51	YES	YES	28	22	69.54	YES	NO
296	F	07/07/69	Lung	3	02/05/21	51	YES	YES	2	21	24.02	YES	YES
298	F	10/02/71	Colorectal	1	01/29/21	49	NO	NS	22	0	0	NO	YES
299	F	03/25/79	Breast	0	01/15/21	41	NO	NO	0	72	3.79	NO	NO
300	M	04/26/49	Urothelial	2	03/19/21	71	YES	NS	2	0	3.79	NO	YES
302	F	01/01/73	Colorectal	5	01/21/21	48	NO	YES	15	61	45.52	NO	NO
303	F	01/16/62	Lung	0	02/05/21	59	YES	NS	0	42	6.32	NO	NO
304	M	07/27/42	Lung	0	02/09/21	78	YES	NS	0	0	6.32	YES	NO
305	F	05/18/63	Ovary	3	02/26/21	57	NO	YES	58	0	1.26	YES	NO
308	F	09/23/73	Breast	2	03/30/21	47	NO	NO	15	31	5.06	NO	NO
309	F	03/25/58	Colorectal	3	02/25/21	62	NS	NS	10	25	8.85	NO	NO
312	M	05/17/71	Stomach	3	02/12/21	49	YES	NO	34	18	5.06	NO	YES
314	F	09/14/41	Ovary	2	02/02/21	79	NS	NS	8	0	3.79	YES	NO
317	F	07/03/58	Uterus	2	01/22/21	62	NO	NO	18	29	3.79	NO	NO
319	M	06/15/70	Lung	0	02/17/21	50	YES	NS	0	0	30.35	NO	NO
320	M	04/03/60	Lung	2	01/18/21	60	YES	YES	1	0	5.06	YES	NO
322	M	02/18/91	Colorectal	2	03/11/21	30	NO	YES	26	0	26.55	YES	NO
324	F	06/22/36	Lung	0	02/12/21	84	NO	NS	3	0	2.53	YES	NO
327	M	09/29/39	Prostate	3	01/12/21	81	NO	NS	24	46	1.26	NO	NO
329	F	05/02/48	Liver and biliary tract	1	02/11/21	72	NO	NO	11	0	5.06	YES	NO
330	M	04/10/71	Lung	4	02/15/21	49	YES	NO	16	0	0	NO	YES

332	F	07/19/42	Unknown	4	03/12/21	78	NO	NS	10	62	11.38	YES	YES
333	M	10/03/75	Colorectal	5	03/04/21	45	NS	YES	19	57	22.76	YES	NO
334	M	10/03/59	Colorectal	5	01/12/21	61	YES	YES	35	21	5.06	NO	NO
336	M	01/21/47	Head&Neck	0	02/09/21	74	YES	NS	22	0	1.26	YES	NO
342	M	07/07/35	Head&Neck	0	03/04/21	85	YES	NS	2	0	0.0	YES	NO
346	M	04/29/64	Unknown	0	03/03/21	56	YES	YES	1	65	3.79	NO	YES
347	F	05/10/78	Colorectal	0	02/09/21	42	YES	NS	2	0	1.26	NO	NO
348	F	04/03/62	Thyroid	2	03/24/21	58	YES	YES	4	0	5.06	YES	NO
351	F	06/18/50	Breast	1	01/22/21	70	NO	YES	46	0	3.79	YES	NO
353	M	04/26/81	Colorectal	0	01/25/21	39	NO	YES	7	0	0.0	NO	NO
354	M	10/07/55	Pancreas	0	02/02/21	65	NS	NS	15	0	2.53	NO	YES
355	F	07/03/61	Unknown	3	01/28/21	59	NO	NO	14	0	7.59	YES	NO
358	F	06/25/51	Pancreas	3	03/09/21	69	NO	NO	92	0	0.0	YES	YES
359	F	07/25/65	Stomach	2	03/09/21	55	YES	NO	8	38	13.91	YES	NO
360	M	09/23/88	Other	8	03/04/21	32	NO	YES	7	15	115.06	YES	YES
362	F	06/13/50	Uterus	0	02/08/21	70	NS	NO	0	0	0.0	YES	YES
363	F	04/14/67	Urothelial	2	03/16/21	53	YES	YES	13	30	17.7	NO	NO
364	M	08/13/53	Urothelial	2	02/04/21	67	NS	YES	91	40	41.72	NO	NO
366	F	06/23/56	Thyroid	2	01/25/21	64	YES	NO	1	22	11.38	YES	YES
367	M	02/26/60	Lung	4	01/21/21	60	YES	YES	27	0	11.38	NO	NO
369	F	06/12/63	Colorectal	2	02/09/21	57	NS	NO	18	0	2.53	YES	NO
377	F	11/04/62	Pancreas	2	12/15/20	58	NO	NO	34	0	3.79	YES	NO
378	F	12/03/81	Colorectal	4	01/08/21	39	NO	YES	0	52	26.55	NO	NO
380	M	11/12/49	Other	6	12/23/20	71	NO	YES	1	0	6.32	YES	NO
381	F	05/08/49	Breast	0	12/17/20	71	YES	YES	116	32	12.64	YES	NO
383	M	03/23/50	Thyroid	6	12/22/20	70	NO	YES	38	0	269.31	YES	NO
388	M	11/10/64	Urothelial	2	01/29/21	56	NS	NO	0	0	5.06	NO	NO
398	M	08/02/48	Liver and biliary tract	2	01/07/21	72	NS	NS	19	0	1.26	YES	NO

401	M	11/21/65	Other	3	01/12/21	55	YES	YES	23	18	3.79	YES	YES
409	F	09/27/63	Lung	1	01/13/21	57	YES	NS	112	0	0	NO	YES
412	M	05/30/66	Lung	3	01/14/21	54	YES	NO	0	54	25.29	NO	NO
414	F	12/28/48	Lung	5	01/14/21	72	NO	YES	75	0	7.59	YES	NO
420	M	09/17/85	Colorectal	0	01/19/21	35	YES	YES	2	72	12.64	YES	NO
425	F	05/09/49	Urothelial	2	01/19/21	71	YES	YES	17	13	10.12	YES	NO
436	M	05/15/64	Urothelial	2	01/21/21	56	YES	NO	138	0	1.26	NO	NO
444	M	09/07/46	Prostate	2	01/25/21	74	NO	YES	2	21	127.7	YES	YES
446	M	11/21/64	Other	1	01/25/21	56	YES	NO	25	0	1.26	YES	NO
449	F	08/23/89	Lung	3	01/26/21	31	NO	YES	13	25	3.79	NO	NO
453	M	10/18/46	Urothelial	1	01/26/21	74	YES	NS	16	0	1.26	NO	YES
461	F	07/28/69	Urothelial	0	01/27/21	51	NO	NS	23	0	0.0	YES	YES
462	M	05/02/64	Colorectal	1	01/28/21	56	YES	YES	0	0	21.49	YES	NO
474	M	06/16/61	Pancreas	4	01/28/21	59	NS	NS	4	0	6.32	YES	NO
476	M	11/25/60	Urothelial	0	01/29/21	60	YES	YES	0	0	3.79	NO	YES
484	M	09/09/84	Colorectal	3	02/01/21	36	NS	YES	46	0	78.39	YES	YES
494	M	08/08/60	Lung	3	02/03/21	60	YES	YES	25	0	12.64	YES	NO
508	M	06/25/57	Prostate	4	02/04/21	63	YES	NS	6	0	2.53	NO	NO
518	M	06/11/58	Liver and biliary tract	0	02/05/21	62	NO	NO	0	0	0	NO	YES
526	F	06/22/88	Breast	5	02/05/21	32	NO	NO	2	0	0	YES	NO
527	F	05/30/66	Pancreas	3	02/08/21	54	YES	NS	6	0	0	NO	YES
536	F	02/09/71	Head&Neck	3	02/09/21	50	YES	NS	24	0	0.0	YES	YES
541	M	11/22/61	Lung	2	02/10/21	59	YES	NO	12	0	2.53	YES	YES
544	F	07/20/46	Breast	7	02/10/21	74	NS	NS	218	0	5.06	YES	NO
560	M	06/13/72	Liver and biliary tract	0	02/11/21	48	NO	YES	6	0	0	NO	YES
566	F	08/06/82	Other	3	02/15/21	38	NO	YES	0	20	3.79	YES	NO
568	F	10/25/63	Liver and biliary tract	1	02/16/21	57	NO	NO	26	0	1.26	NO	YES
577	F	01/01/53	Urothelial	1	02/17/21	68	YES	YES	33	0	10.12	NO	NO

580	M	12/29/57	Colorectal	8	02/17/21	63	NO	YES	42	17	7.59	NO	NO
582	M	12/30/61	Lung	2	02/18/21	59	YES	YES	12	0	2.53	YES	NO
583	M	05/18/61	Oesophagus	0	02/18/21	59	YES	NO	8	0	2.53	YES	NO
589	F	01/24/61	Uterus	3	02/18/21	60	NS	NS	59	0	2.53	YES	NO
601	M	12/09/43	Lung	0	02/22/21	77	NO	YES	0	0	6.32	NO	NO
609	F	05/06/91	Breast	3	02/23/21	29	NS	NS	13	0	1.26	NO	NO
610	F	04/10/58	Uterus	0	02/23/21	62	NS	NO	189	0	2.53	NO	NO
627	M	02/01/49	Prostate	5	02/24/21	72	YES	NO	45	0	5.06	YES	NO
629	M	12/15/47	Head&Neck	1	02/25/21	73	YES	NS	8	0	13.91	YES	NO
633	F	04/11/61	Lung	1	02/26/21	59	NO	NO	42	0	2.53	YES	NO
647	M	04/10/52	Prostate	1	03/02/21	68	YES	NO	38	0	3.79	YES	NO
649	M	11/14/51	Lung	1	03/02/21	69	YES	YES	6	50	18.97	NO	NO
650	F	01/01/69	Colorectal	2	03/02/21	52	NO	YES	3	0	0	NO	YES
658	M	10/05/43	Colorectal	2	03/03/21	77	NO	YES	11	47	10.12	NO	NO
671	F	05/26/81	Uterus	4	03/04/21	39	NS	NS	59	11	1.26	YES	NO
675	F	12/09/63	Other	0	03/04/21	57	NS	NS	8	0	1.26	NO	YES
687	M	06/06/64	Lung	1	03/08/21	56	YES	YES	9	45	20.23	YES	YES
688	M	04/22/41	Head&Neck	0	03/08/21	79	YES	NS	7	0	8.85	YES	NO
689	M	10/17/64	Head&Neck	3	03/09/21	56	YES	NO	33	46	8.85	NO	NO
690	F	06/28/77	Breast	2	03/09/21	43	NO	NS	40	0	0.0	YES	NO
692	M	02/22/58	Urothelial	2	03/09/21	63	YES	NS	22	0	10.12	YES	NO
715	F	02/12/54	Uterus	2	03/12/21	67	NS	NO	0	0	1.26	NO	NO
718	M	02/02/59	Liver and biliary tract	2	03/12/21	62	NO	NO	36	0	6.32	NO	YES
749	M	11/19/58	Urothelial	1	03/17/21	62	YES	YES	1	21	15.17	YES	NO
757	M	03/03/53	Prostate	4	03/17/21	68	YES	NS	26	14	2.53	YES	NO
758	M	04/28/64	Lung	0	03/18/21	56	YES	YES	0	32	10.12	YES	NO
759	F	06/21/63	Stomach	2	03/18/21	57	NS	NS	14	0	2.53	NO	NO
764	M	05/16/59	Pancreas	2	03/18/21	61	NS	NS	44	46	27.82	YES	YES

767	F	05/05/77	Breast	2	03/19/21	43	YES	NS	5	0	1.26	NO	NO
768	F	04/23/57	Head&Neck	3	03/19/21	63	NO	NS	48	0	2.53	YES	NO
789	M	02/13/84	Other	2	03/23/21	37	NS	NS	14	13	0	NO	YES
819	M	05/16/53	Colorectal	4	03/26/21	67	NS	NS	40	46	21.49	NO	NO
825	M	11/12/66	Pancreas	1	03/26/21	54	NS	NS	9	0	1.26	NO	YES
840	F	12/12/67	Uterus	1	03/30/21	53	NS	YES	20	0	3.79	YES	NO
845	M	07/13/55	Colorectal	0	03/31/21	65	NO	NS	15	0	1.26	YES	YES
861	F	09/10/80	Breast	4	04/20/21	40	NO	NS	9	53	2.53	NO	NO
863	F	10/04/58	Other	0	04/21/21	62	YES	YES	0	0	22.76	YES	YES
868	F	03/27/89	Breast	0	02/22/22	32	NS	NS	12	0	0.0	NO	NO
875	F	08/04/71	Unknown	0	11/03/21	50	NO	NS	18	0	0.0	YES	NO
876	F	11/16/47	Unknown	0	05/14/21	73	NO	NS	2	0	1.26	YES	NO
880	M	02/24/66	Colorectal	3	05/05/21	55	NS	YES	32	55	24.02	NO	NO
884	M	09/15/56	Thyroid	0	09/16/21	65	YES	NS	6	0	1.26	YES	NO
888	F	08/27/60	Breast	4	04/23/21	60	NO	NS	24	11	5.06	YES	NO
898	M	09/02/47	Urothelial	0	05/25/21	73	YES	YES	27	24	2.53	YES	NO
901	F	07/09/56	Lung	1	04/20/21	64	YES	YES	11	15	11.38	YES	NO
902	M	05/31/68	Other	1	04/21/21	52	YES	YES	20	18	3.79	YES	NO
908	M	04/10/61	Pancreas	1	04/23/21	60	YES	NS	0	0	0	NO	YES
912	F	05/17/60	Colorectal	0	04/21/21	60	NO	NS	19	0	2.53	YES	YES
917	M	04/25/58	Lung	3	05/05/21	63	NO	YES	7	69	2.53	YES	NO
918	F	05/25/60	Urothelial	3	10/21/21	61	NS	NS	59	11	3.79	YES	NO
922	M	10/18/73	Colorectal	1	05/06/21	47	NS	NO	7	0	0.0	NO	NO
927	F	04/12/48	Pancreas	1	05/06/21	73	YES	NS	0	0	0.0	NO	NO
929	M	07/09/51	Lung	2	04/20/21	69	YES	NS	0	59	48.05	YES	NO
938	F	08/10/73	Colorectal	2	04/28/21	47	NS	NO	19	10	12.64	NO	NO
940	M	08/01/57	Prostate	4	04/22/21	63	YES	NS	51	21	3.79	YES	NO
962	F	12/15/80	Breast	5	04/16/21	40	NO	NS	4	38	10.12	YES	YES

963	M	03/09/85	Urothelial	2	04/27/21	36	YES	YES	17	42	1.26	NO	NO
971	M	07/15/61	Lung	1	05/12/21	59	YES	YES	3	0	3.79	YES	YES
978	M	01/26/58	Head&Neck	1	04/28/21	63	YES	YES	11	28	7.59	YES	YES
979	F	12/01/51	Breast	3	10/18/21	69	YES	NO	70	0	8.85	NO	NO
983	F	06/12/50	Other	1	06/16/21	71	NO	NS	43	0	12.64	YES	YES
987	F	09/25/62	Colorectal	2	10/26/21	59	NO	NS	38	0	1.26	YES	NO
1004	M	08/19/60	Prostate	3	05/03/21	60	NO	NS	37	31	1.26	NO	NO
1009	M	09/13/42	Lung	0	04/29/21	78	YES	YES	0	0	2.53	YES	YES
1013	M	08/01/52	Unknown	0	06/01/21	68	YES	YES	0	0	2.53	YES	NO
1017	F	10/29/63	Colorectal	3	04/29/21	57	YES	YES	31	65	6.32	NO	NO
1021	M	10/31/56	Thyroid	2	04/29/21	64	YES	NS	35	0	5.06	YES	NO
1022	F	03/03/63	Pancreas	8	04/28/21	58	NO	NO	5	34	17.7	YES	YES
1029	M	12/31/70	Colorectal	1	05/05/21	50	NO	NS	16	0	1.26	NO	NO
1032	F	06/23/76	Lung	2	05/21/21	44	YES	YES	16	0	15.17	NO	NO
1034	F	04/24/56	Breast	1	04/29/21	65	NO	YES	74	0	5.06	YES	YES
1036	F	07/18/52	Colorectal	1	05/10/21	68	YES	YES	10	0	3.79	YES	NO
1045	M	08/13/57	Prostate	3	05/12/21	63	YES	NS	15	23	3.79	NO	NO
1056	M	05/03/71	Lung	0	05/12/21	50	NO	YES	0	0	1.26	YES	NO
1066	F	05/18/69	Breast	1	06/09/21	52	YES	NS	7	0	1.26	NO	NO
1072	F	04/07/47	Breast	0	06/03/21	74	NO	YES	0	0	0.0	YES	YES
1084	F	07/05/37	Lung	0	05/19/21	83	YES	YES	1	0	5.06	YES	NO
1086	F	05/12/77	Pancreas	2	05/14/21	44	YES	NO	7	35	8.85	NO	NO
1093	F	11/23/48	Uterus	0	06/08/21	72	YES	NS	0	29	0	NO	YES
1099	M	01/18/62	Liver and biliary tract	3	05/28/21	59	NS	NS	26	18	6.32	NO	NO
1108	F	07/23/55	Colorectal	7	09/23/21	66	NO	YES	30	10	3.79	YES	NO
1119	F	01/08/51	Ovary	2	08/23/21	70	NO	NS	45	14	5.06	YES	NO
1130	F	10/25/78	Stomach	1	06/03/21	42	NO	NS	15	0	0	NO	YES
1132	F	06/14/85	Stomach	1	06/03/21	35	NO	NS	11	0	2.53	NO	NO

1134	M	09/22/64	Pancreas	2	06/03/21	56	YES	NS	51	0	2.53	YES	NO
1135	M	07/21/88	Colorectal	2	07/02/21	32	NO	NO	29	0	0.0	NO	NO
1136	F	09/05/48	Lung	0	05/16/21	72	YES	YES	0	0	10.12	YES	NO
1148	F	05/06/58	Uterus	2	05/20/21	63	YES	NO	39	0	6.32	YES	YES
1149	F	02/25/62	Breast	4	05/18/21	59	NO	NO	15	0	3.79	YES	NO
1161	F	12/31/58	Lung	2	06/09/21	62	NO	NO	10	0	2.53	YES	NO
1164	F	01/06/62	Lung	0	06/02/22	60	YES	NO	14	40	5.06	YES	NO
1165	F	03/11/63	Lung	1	05/31/21	58	YES	YES	8	19	7.59	YES	NO
1173	F	02/07/53	Pancreas	1	06/10/21	68	NS	NS	3	0	1.26	YES	NO
1174	F	02/07/96	Colorectal	1	06/09/21	25	NO	YES	8	0	0	NO	YES
1179	F	08/08/70	Breast	1	05/11/21	50	YES	NS	32	0	7.59	YES	NO
1186	M	10/18/58	Stomach	3	05/28/21	62	NO	NO	29	0	6.32	NO	NO
1190	F	07/09/81	Breast	0	05/28/21	39	NS	NS	4	0	1.26	NO	YES
1193	F	04/01/48	Liver and biliary tract	1	05/23/21	73	NO	NO	11	45	7.59	NO	NO
1201	F	06/04/74	Liver and biliary tract	2	04/30/21	46	NS	NS	4	0	2.53	YES	NO
1203	M	10/07/39	Pancreas	1	05/17/21	81	YES	NS	5	0	3.79	YES	NO
1214	F	12/21/39	Ovary	2	06/25/21	81	NO	NS	173	0	0.0	YES	YES
1220	M	04/22/51	Urothelial	1	04/29/21	70	NO	NS	1	49	24.02	NO	NO
1223	F	03/27/63	Breast	1	06/03/21	58	NO	NO	25	27	31.61	NO	NO
1225	M	12/20/54	Colorectal	4	06/09/21	66	YES	YES	35	0	1.26	NO	NO
1229	F	04/22/42	Breast	1	04/27/21	79	NO	NS	30	0	3.79	YES	YES
1234	F	12/25/73	Pancreas	2	06/17/21	47	NS	YES	0	0	1.26	NO	NO
1237	F	05/01/82	Breast	0	06/15/21	39	NO	NS	0	0	2.53	NO	NO
1240	M	08/17/53	Stomach	0	06/08/21	67	YES	YES	0	0	2.53	YES	YES
1250	F	02/20/85	Thyroid	0	04/15/22	37	NO	NO	14	13	3.79	YES	YES
1260	M	09/14/78	Lung	1	09/29/21	43	NO	NO	13	11	1.26	NO	NO
1261	F	04/26/67	Head&Neck	1	06/22/21	54	NO	NS	10	0	3.79	YES	YES
1263	F	04/16/49	Breast	0	07/27/22	73	NO	NS	14	0	21.49	YES	NO

1270	F	12/13/83	Liver and biliary tract	1	06/01/21	37	NS	NS	0	0	2.53	NO	NO
1272	M	01/28/65	Prostate	3	10/05/21	56	NO	NO	19	0	1.26	YES	NO
1273	M	04/26/54	Oesophagus	1	06/25/21	67	NO	YES	7	0	10.12	NO	NO
1278	F	05/14/75	Breast	6	05/25/21	46	NO	YES	13	13	3.79	NO	NO
1287	M	11/16/52	Head&Neck	1	10/04/21	68	YES	YES	4	0	15.17	YES	YES
1292	F	02/21/50	Ovary	2	06/08/21	71	NO	YES	7	0	0.0	YES	NO
1298	M	09/29/55	Prostate	4	06/24/21	65	NS	YES	113	31	10.12	YES	NO
1313	F	12/28/90	Lung	1	06/11/21	30	NO	NO	9	0	0.0	NO	NO
1316	F	10/23/48	Colorectal	2	05/26/21	72	NS	YES	9	0	0.0	YES	NO
1329	F	07/27/63	Breast	0	09/23/21	58	YES	NS	2	0	3.79	NO	NO
1330	M	03/05/50	Lung	0	06/23/21	71	YES	NS	0	0	1.26	NO	YES
1338	M	03/20/55	Colorectal	1	06/18/21	66	YES	YES	0	0	5.06	NO	NO
1355	F	12/29/65	Lung	4	06/11/21	55	YES	NS	0	0	8.85	YES	NO
1356	F	12/29/66	Other	1	07/06/21	54	YES	YES	1	0	1.26	YES	NO
1363	F	04/07/42	Liver and biliary tract	2	07/09/21	79	NO	NS	9	0	2.53	YES	NO
1371	M	05/25/77	Other	5	06/11/21	44	NS	NS	95	0	0	NO	YES
1376	F	10/06/46	Other	6	05/19/21	74	NO	NS	96	0	5.06	YES	NO
1384	M	03/08/78	Lung	2	07/05/21	43	YES	YES	2	0	20.23	YES	YES
1390	M	10/15/47	Lung	0	06/30/21	73	YES	NS	24	0	12.64	NO	NO
1392	F	10/29/37	Colorectal	2	06/28/21	83	NO	NO	21	0	2.53	NO	NO
1396	F	12/01/56	Breast	1	07/06/21	64	NO	NS	4	0	2.53	NO	YES
1402	M	04/23/57	Oesophagus	1	06/22/21	64	YES	NO	25	41	11.38	NO	NO
1404	F	08/12/47	Liver and biliary tract	2	06/14/21	73	YES	NS	30	0	3.79	YES	NO
1407	F	03/08/64	Colorectal	4	07/15/21	57	NS	NS	53	46	20.23	YES	NO
1408	F	11/12/59	Colorectal	1	07/15/21	61	NO	NS	16	0	0.0	NO	YES
1410	F	12/31/60	Pancreas	1	07/16/21	60	NS	NS	19	0	1.26	YES	YES
1415	F	03/04/63	Ovary	4	11/30/21	58	NO	NS	41	0	3.79	YES	YES
1417	M	05/27/43	Liver and biliary tract	0	07/09/21	78	YES	YES	1	0	1.26	NO	NO

1418	F	06/20/75	Stomach	1	06/24/21	46	NS	NS	27	0	5.06	YES	NO
1421	F	08/22/51	Uterus	3	06/24/21	69	NO	NS	0	10	8.85	YES	NO
1422	M	05/21/52	Oesophagus	3	06/24/21	69	YES	NS	19	28	16.44	NO	NO
1432	M	07/14/77	Pancreas	1	07/22/21	44	YES	YES	2	0	1.26	NO	YES
1435	F	04/08/59	Colorectal	2	08/13/21	62	NO	NO	30	0	2.53	NO	NO
1436	M	10/20/43	Prostate	3	07/22/21	77	YES	NS	94	0	5.06	YES	YES
1455	M	03/10/53	Lung	1	07/13/21	68	YES	YES	11	0	0.0	NO	NO
1456	F	04/30/58	Lung	1	07/08/21	63	NO	NS	26	0	1.26	NO	YES
1457	F	05/10/81	Breast	3	07/09/21	40	YES	NS	0	0	0.0	NO	NO
1458	F	03/16/81	Breast	1	07/01/21	40	NO	NS	15	0	2.53	NO	NO
1461	F	05/05/46	Breast	1	09/29/21	75	NO	NS	34	0	1.26	YES	NO
1466	F	05/27/69	Other	0	07/20/21	52	NO	YES	16	10	5.06	NO	NO
1469	F	12/19/38	Other	0	08/02/21	82	NO	YES	0	11	5.06	YES	YES
1472	M	03/04/72	Pancreas	2	06/08/21	49	YES	NS	0	0	1.26	NO	YES
1475	F	11/20/58	Colorectal	0	06/21/21	62	YES	NO	0	0	1.26	NO	YES
1476	F	06/05/68	Liver and biliary tract	3	06/25/21	53	NS	NO	15	0	0.0	YES	NO
1477	F	07/27/51	Pancreas	0	06/21/21	69	YES	YES	0	0	2.53	YES	NO
1479	F	12/11/51	Lung	0	05/31/21	69	NO	YES	1	0	13.91	YES	YES
1480	M	05/23/49	Stomach	8	07/07/21	72	YES	YES	21	49	22.76	YES	NO
1482	F	06/09/61	Colorectal	2	07/08/21	60	NS	NO	15	25	8.85	NO	NO
1483	F	09/25/64	Colorectal	1	07/29/21	56	NS	NS	10	0	3.79	NO	NO
1485	F	08/04/78	Breast	2	06/29/21	42	NO	YES	18	0	1.26	NO	YES
1488	M	03/24/86	Urothelial	2	08/03/21	35	NO	YES	80	13	1.26	YES	NO
1494	F	09/30/65	Colorectal	3	07/29/21	55	NO	NO	30	68	22.76	NO	YES
1496	M	08/03/62	Colorectal	3	07/26/21	58	NS	NO	46	0	0	NO	YES
1501	M	05/28/66	Lung	0	07/13/21	55	YES	NO	16	0	2.53	NO	YES
1502	F	09/12/55	Lung	1	06/08/21	65	NO	NS	16	0	3.79	YES	NO
1509	F	12/12/72	Lung	2	07/13/21	48	YES	NS	40	11	7.59	NO	NO

1515	M	03/21/47	Prostate	3	07/13/21	74	YES	NO	35	0	6.32	YES	NO
1528	M	06/05/60	Oesophagus	0	06/30/21	61	NO	NS	25	0	2.53	NO	YES
1537	M	03/03/55	Prostate	1	07/28/21	66	NO	NS	32	25	3.79	NO	NO
1541	M	12/02/71	Lung	2	07/28/21	49	YES	YES	27	72	45.52	YES	YES
1547	F	04/06/45	Breast	0	08/05/21	76	YES	NS	52	0	1.26	YES	NO
1549	F	10/03/35	Uterus	1	07/29/21	85	NO	YES	22	0	30.35	YES	NO
1552	F	03/18/45	Uterus	1	07/02/21	76	NO	NO	28	0	16.44	YES	YES
1553	M	04/04/56	Colorectal	2	08/05/21	65	NS	NS	9	11	1.26	YES	YES
1558	M	05/18/57	Colorectal	3	08/05/21	64	NS	NS	18	0	7.59	YES	NO
1561	F	09/21/63	Pancreas	2	08/05/21	57	YES	NS	7	0	1.26	YES	NO
1566	F	01/20/68	Liver and biliary tract	1	06/04/21	53	NO	NO	19	0	2.53	NO	YES
1568	M	01/01/55	Stomach	0	07/08/21	66	YES	NS	1	0	2.53	NO	NO
1569	M	04/14/78	Pancreas	2	07/08/21	43	YES	NO	4	0	1.26	NO	NO
1572	M	02/07/65	Lung	1	12/30/21	56	YES	NS	14	35	17.7	NO	NO
1587	F	05/25/57	Lung	8	07/20/21	64	YES	NS	78	0	21.49	YES	NO
1588	M	04/15/50	Colorectal	1	08/16/21	71	YES	NS	25	0	3.79	YES	NO
1609	F	02/21/50	Colorectal	4	08/12/21	71	NS	NS	50	27	8.85	YES	NO
1619	F	03/12/50	Breast	11	10/01/21	71	NO	NS	48	0	3.79	YES	NO
1622	F	06/11/93	Breast	2	08/31/21	28	NS	NS	1	0	2.53	NO	NO
1627	F	01/11/95	Ovary	1	10/07/21	26	NO	NS	9	11	6.32	NO	NO
1629	F	03/25/50	Lung	2	08/03/21	71	NO	NO	6	0	3.79	YES	NO
1638	M	04/08/63	Prostate	2	08/05/21	58	NO	NO	6	0	2.53	YES	NO
1661	M	03/11/51	Liver and biliary tract	1	07/16/21	70	NS	YES	28	0	2.53	NO	NO
1666	M	02/06/51	Colorectal	0	09/02/21	70	NO	YES	8	0	1.26	YES	YES
1674	M	12/20/43	Lung	1	08/30/21	77	YES	YES	4	28	17.7	YES	NO
1675	M	05/17/59	Lung	4	09/02/21	62	YES	NS	12	0	3.79	NO	NO
1676	F	06/28/67	Lung	1	08/23/21	54	YES	NS	7	0	6.32	NO	NO
1679	M	09/03/64	Lung	0	08/04/21	56	YES	NO	0	0	2.53	NO	YES

1680	M	06/29/53	Other	2	03/15/22	68	NO	NS	8	0	1.26	YES	NO
1685	M	07/18/74	Lung	0	08/31/21	47	YES	YES	3	0	0.0	NO	NO
1694	M	09/11/47	Urothelial	3	06/30/21	73	YES	YES	18	18	1.26	YES	YES
1695	F	01/31/65	Head&Neck	1	06/08/21	56	NO	NS	16	0	1.26	YES	NO
1696	M	02/07/65	Head&Neck	1	09/14/21	56	YES	NS	13	0	10.12	NO	NO
1702	M	10/01/71	Colorectal	2	08/31/21	49	NO	YES	52	21	7.59	NO	NO
1706	M	02/22/37	Colorectal	4	08/26/21	84	NS	NO	18	28	16.44	NO	NO
1707	F	10/02/65	Liver and biliary tract	2	08/26/21	55	NS	NS	1	0	2.53	YES	NO
1716	F	05/31/57	Lung	4	08/24/21	64	YES	YES	31	0	8.85	NO	NO
1724	F	05/21/56	Thyroid	1	02/16/22	65	NO	NO	12	13	2.53	NO	YES
1730	M	07/10/52	Prostate	5	06/18/21	68	YES	NS	0	36	13.91	YES	NO
1739	F	10/05/68	Colorectal	2	07/30/21	52	NO	YES	75	0	3.79	YES	YES
1740	F	10/09/54	Liver and biliary tract	2	07/30/21	66	NO	YES	23	0	2.53	YES	NO
1742	M	09/22/54	Urothelial	3	08/19/21	66	YES	YES	19	0	13.91	YES	NO
1745	M	08/16/50	Prostate	2	08/19/21	71	YES	NS	119	51	2.53	NO	NO
1752	M	01/02/53	Oesophagus	1	08/23/21	68	NO	YES	13	0	0.0	NO	NO
1759	F	01/20/49	Colorectal	0	08/20/21	72	NO	NS	5	0	1.26	YES	YES
1763	M	04/29/64	Head&Neck	2	08/24/21	57	YES	YES	6	0	5.06	NO	NO
1777	M	08/22/77	Colorectal	1	08/26/21	44	YES	NS	55	34	8.85	NO	NO
1779	M	08/03/45	Colorectal	1	08/27/21	76	YES	NS	49	0	3.79	NO	YES
1786	F	11/05/63	Uterus	0	08/02/21	57	YES	YES	2	0	3.79	YES	NO
1787	M	07/21/74	Lung	0	09/13/21	47	YES	YES	0	21	3.79	NO	NO
1798	M	08/01/58	Prostate	2	08/19/21	63	YES	YES	32	0	26.55	YES	NO
1819	M	01/03/56	Prostate	1	09/17/21	65	YES	YES	1	34	2.53	YES	NO
1820	M	03/17/58	Head&Neck	4	08/20/21	63	NO	NS	39	0	1.26	YES	NO
1821	F	12/16/63	Pancreas	0	08/27/21	57	YES	NO	36	0	3.79	NO	NO
1822	M	03/20/73	Colorectal	1	09/01/21	48	NS	NS	4	32	3.79	NO	YES
1841	M	07/22/97	Colorectal	1	09/16/21	24	NO	NS	8	0	0	NO	YES

1842	M	09/28/68	Colorectal	2	07/08/21	52	NO	NO	18	0	6.32	NO	NO
1853	M	02/01/66	Pancreas	2	07/15/21	55	NS	NS	6	0	1.26	NO	YES
1859	F	12/05/60	Unknown	3	10/11/21	60	NS	NO	28	13	5.06	YES	NO
1863	F	08/15/76	Breast	2	09/14/21	45	NO	NO	15	12	2.53	YES	NO
1868	F	08/30/59	Liver and biliary tract	1	09/13/21	62	NS	NS	8	0	2.53	YES	YES
1871	M	08/05/51	Pancreas	1	09/14/21	70	NS	NS	5	0	5.06	NO	NO
1872	M	04/10/46	Pancreas	2	09/17/21	75	NO	NS	28	0	1.26	NO	YES
1883	F	01/26/62	Colorectal	0	09/13/21	59	NO	NO	0	0	1.26	YES	YES
1894	F	03/06/91	Stomach	2	09/16/21	30	NO	NS	14	0	0.0	NO	NO
1899	F	03/09/61	Colorectal	5	09/14/21	60	NO	NS	52	29	10.12	NO	NO
1901	F	03/31/52	Uterus	0	05/31/21	69	NO	YES	5	0	3.79	YES	YES
1910	M	10/08/50	Unknown	0	01/28/21	70	NS	NS	2	0	2.53	NO	YES
1932	F	02/26/75	Other	0	09/20/21	46	NO	NS	2	0	0.0	NO	YES
1936	F	04/23/66	Lung	0	09/22/21	55	YES	NS	0	0	1.26	YES	NO
1937	F	03/24/60	Lung	0	09/21/21	61	YES	NS	4	0	3.79	NO	YES
1942	M	12/25/66	Colorectal	0	09/22/21	54	NO	NO	15	0	1.26	NO	YES
1944	M	03/25/68	Colorectal	2	09/23/21	53	NO	YES	62	43	5.06	NO	NO
1947	M	09/29/58	Stomach	3	09/23/21	62	YES	YES	0	29	16.44	YES	NO
1952	M	02/16/53	Urothelial	1	12/20/21	68	YES	YES	5	11	10.12	YES	NO
1954	F	09/24/89	Colorectal	0	10/21/21	32	NO	YES	11	0	2.53	NO	NO
1956	M	02/17/84	Colorectal	1	09/30/21	37	NO	NS	58	0	7.59	NO	NO
1957	F	02/26/64	Colorectal	1	09/30/21	57	YES	NS	42	0	2.53	NO	NO
1958	M	03/20/66	Stomach	2	09/30/21	55	NO	NS	7	0	3.79	NO	NO
1960	M	09/22/49	Other	1	09/30/21	72	NS	NS	16	15	3.79	YES	NO
1961	F	11/26/62	Colorectal	2	09/30/21	58	NO	NS	34	0	2.53	YES	YES
1965	M	10/06/99	Pancreas	0	09/20/21	21	YES	YES	0	13	5.06	NO	NO
1979	F	11/30/88	Breast	0	10/07/21	32	NO	NS	7	0	5.06	NO	NO
1985	F	10/10/55	Breast	7	09/28/21	65	NS	NS	78	34	8.85	YES	NO

1991	F	02/01/78	Lung	1	09/30/21	43	YES	NO	87	0	0.0	YES	YES
1995	F	06/29/52	Colorectal	2	09/14/21	69	NO	NO	15	0	1.26	YES	NO
1997	M	05/25/55	Oesophagus	2	09/27/21	66	YES	YES	0	11	8.85	NO	YES
1998	F	06/27/72	Lung	1	10/05/21	49	YES	NO	6	30	16.44	NO	NO
2011	F	01/17/50	Uterus	1	09/15/21	71	NO	NS	23	0	2.53	YES	YES
2013	F	03/11/56	Liver and biliary tract	0	10/07/21	65	NS	NS	1	48	6.32	NO	NO
2014	M	12/19/83	Oesophagus	2	10/07/21	37	NS	NS	10	25	3.79	NO	NO
2018	F	07/06/62	Colorectal	0	10/11/21	59	NS	NS	1	0	0.0	NO	YES
2019	M	05/10/53	Colorectal	4	10/04/21	68	YES	NS	54	25	8.85	YES	NO
2020	F	07/31/72	Colorectal	3	10/04/21	49	NO	NS	4	0	5.06	YES	NO
2045	M	05/18/59	Head&Neck	1	09/07/21	62	YES	NO	0	0	2.53	YES	YES
2052	F	05/27/66	Colorectal	0	10/11/21	55	NS	NS	0	24	5.06	NO	NO
2065	M	09/27/60	Head&Neck	0	10/18/21	61	NO	NS	0	0	1.26	NO	YES
2069	M	06/01/40	Head&Neck	0	11/09/21	81	NS	NS	2	0	1.26	NO	YES
2071	F	03/01/50	Head&Neck	1	08/10/21	71	NO	YES	14	17	1.26	YES	NO
2091	F	02/14/57	Breast	9	09/28/21	64	NS	NS	83	0	2.53	YES	NO
2094	F	10/21/53	Uterus	2	09/29/21	67	YES	NS	34	0	5.06	YES	NO
2102	F	04/19/62	Pancreas	2	10/05/21	59	NS	NO	10	0	6.32	YES	NO
2111	F	02/15/57	Pancreas	2	10/07/21	64	NS	NS	34	0	1.26	YES	YES
2113	F	05/19/49	Pancreas	1	07/21/22	73	NO	NS	17	25	10.12	YES	NO
2120	F	12/23/47	Breast	6	09/24/21	73	NO	NS	5	11	8.85	NO	NO
2124	M	03/27/65	Colorectal	0	08/27/21	56	NO	YES	0	0	0.0	YES	YES
2131	M	09/02/66	Colorectal	2	10/22/21	55	YES	NO	22	0	0.0	NO	NO
2138	F	03/31/58	Breast	6	10/01/21	63	YES	NS	34	0	20.23	YES	NO
2151	F	02/13/51	Uterus	3	09/01/21	70	NO	NO	27	28	8.85	NO	NO
2162	M	05/23/57	Stomach	2	10/21/21	64	YES	NS	10	0	1.26	YES	YES
2166	F	11/11/55	Pancreas	1	08/18/21	65	NO	NS	5	0	0	NO	YES
2169	M	12/14/74	Stomach	1	09/02/21	46	NO	YES	11	33	15.17	YES	YES

2171	F	10/11/53	Colorectal	3	09/22/21	67	YES	YES	6	58	11.38	NO	NO
2181	F	04/25/68	Lung	0	08/25/21	53	YES	YES	0	0	16.44	YES	NO
2187	M	08/02/48	Lung	0	10/13/21	73	NO	NO	1	14	2.53	NO	NO
2199	F	11/19/82	Colorectal	3	12/31/21	39	NS	YES	3	22	10.12	NO	NO
2207	M	11/03/47	Prostate	4	10/27/21	73	YES	NO	17	12	8.85	NO	NO
2208	M	12/31/49	Prostate	0	12/02/21	71	NO	NO	2	0	1.26	NO	YES
2217	M	02/21/43	Prostate	0	11/03/21	78	YES	YES	2	19	1.26	NO	NO
2221	M	11/17/62	Lung	0	10/29/21	58	YES	YES	1	0	0.0	NO	NO
2227	F	04/05/69	Ovary	2	10/27/21	52	YES	NS	23	0	1.26	NO	NO
2231	F	09/22/81	Colorectal	3	11/04/21	40	NS	NS	16	0	6.32	NO	NO
2232	M	05/04/82	Other	0	10/05/21	39	NO	NS	2	0	0	NO	YES
2239	M	12/15/56	Colorectal	1	10/20/21	64	YES	NS	12	17	10.12	NO	NO
2241	F	01/18/56	Pancreas	0	11/23/21	65	NO	YES	11	0	1.26	YES	NO
2296	F	10/21/52	Colorectal	1	11/24/21	69	YES	YES	10	38	5.06	NO	NO
2303	M	05/14/55	Lung	2	10/25/21	66	YES	NS	7	42	3.79	NO	NO
2316	M	11/01/47	Colorectal	0	10/14/21	73	YES	YES	0	0	1.26	NO	YES
2347	F	08/22/50	Colorectal	1	10/26/21	71	NS	NO	5	0	3.79	NO	YES
2348	F	04/20/48	Breast	2	02/03/22	73	YES	NS	11	0	5.06	YES	NO
2352	M	02/13/63	Lung	5	10/26/21	58	NS	YES	44	0	10.12	YES	NO
2367	M	06/24/48	Colorectal	2	11/22/21	73	NO	YES	14	13	13.91	YES	NO
2372	M	11/27/62	Colorectal	3	11/18/21	58	NS	NS	70	39	18.97	NO	NO
2373	F	01/06/48	Colorectal	2	11/18/21	73	NS	YES	23	0	3.79	YES	NO
2374	F	09/18/66	Ovary	3	11/10/21	55	NO	NS	73	0	0.0	YES	YES
2398	F	08/27/54	Oesophagus	3	11/02/21	67	NS	NO	31	0	1.26	YES	NO
2416	M	09/26/49	Colorectal	1	11/10/21	72	NS	NS	15	18	1.26	NO	YES
2419	F	04/28/46	Colorectal	3	11/15/21	75	YES	YES	0	16	2.53	YES	NO
2424	F	03/26/73	Breast	3	11/17/21	48	NO	NO	69	0	1.26	YES	NO
2428	M	10/31/71	Oesophagus	1	11/12/21	50	NO	NO	11	35	7.59	YES	YES

2439	M	07/09/73	Lung	0	12/02/21	48	YES	YES	6	0	1.26	NO	YES
2451	M	06/02/37	Prostate	3	11/19/21	84	YES	NS	88	0	5.06	YES	NO
2455	M	03/28/53	Prostate	5	11/23/21	68	NS	NS	38	15	3.79	NO	NO
2458	M	12/30/47	Thyroid	1	12/13/21	73	NO	YES	8	0	3.79	YES	NO
2459	F	07/31/50	Colorectal	1	11/25/21	71	NS	NS	25	12	7.59	NO	NO
2461	M	12/15/47	Pancreas	2	11/25/21	73	YES	NS	7	0	6.32	NO	NO
2462	M	07/18/49	Liver and biliary tract	3	11/25/21	72	NS	NS	26	0	5.06	YES	YES
2468	M	09/29/74	Liver and biliary tract	3	11/23/21	47	NS	YES	0	0	1.26	NO	NO
2470	M	05/02/46	Colorectal	4	11/26/21	75	NO	NS	59	0	3.79	NO	YES
2477	M	12/23/50	Liver and biliary tract	0	12/07/21	70	YES	NS	1	20	10.12	YES	NO
2478	M	06/15/56	Pancreas	0	12/02/21	65	NO	NO	0	0	0.0	YES	YES
2485	F	09/29/71	Ovary	1	10/29/21	50	NO	YES	38	0	1.26	NO	NO
2499	M	12/15/49	Lung	0	11/25/21	71	YES	YES	1	25	3.79	YES	NO
2507	F	07/08/58	Stomach	1	12/14/21	63	NS	NS	86	0	7.59	NO	NO
2523	F	10/18/55	Colorectal	2	10/05/21	65	NS	NS	8	0	7.59	NO	NO
2529	M	07/21/48	Head&Neck	0	12/14/21	73	YES	NS	9	0	0.0	NO	YES
2575	M	10/09/61	Lung	4	12/01/21	60	YES	NO	35	0	2.53	YES	NO
2590	M	03/21/61	Head&Neck	1	01/17/22	60	YES	NO	7	0	3.79	YES	YES
2592	M	06/22/67	Lung	0	11/23/21	54	NO	NO	0	0	5.06	NO	NO
2607	F	11/14/69	Uterus	4	12/15/21	52	NS	NS	52	0	2.53	YES	NO
2610	F	12/13/63	Colorectal	2	12/16/21	58	NS	NS	18	0	3.79	NO	YES
2612	M	05/31/57	Pancreas	3	12/16/21	64	NS	NS	0	11	5.06	NO	NO
2617	M	01/20/71	Stomach	0	12/09/21	50	YES	NS	2	0	1.26	NO	NO
2631	F	01/03/37	Lung	0	12/28/21	84	YES	YES	0	0	13.91	NO	NO
2635	F	03/30/52	Lung	0	11/16/21	69	YES	NS	18	0	2.53	YES	YES
2651	M	02/14/50	Lung	3	12/24/21	71	YES	YES	20	0	5.06	YES	NO
2665	F	09/03/66	Pancreas	0	12/09/21	55	NO	NO	0	0	0.0	NO	YES
2667	F	12/01/53	Other	1	12/10/21	68	NS	NS	2	0	1.26	YES	YES

2670	F	10/14/91	Breast	2	12/24/21	30	YES	NS	17	0	0.0	YES	NO
2672	F	05/23/46	Breast	1	02/01/22	75	YES	NS	3	0	0.0	YES	YES
2689	M	02/10/55	Other	3	01/05/22	66	YES	NO	44	15	10.12	YES	NO
2691	F	10/02/45	Liver and biliary tract	3	01/25/22	76	NO	YES	41	11	3.79	YES	NO
2697	F	05/28/63	Head&Neck	1	12/27/21	58	NO	NO	29	0	0.0	NO	YES
2726	F	10/19/62	Ovary	0	12/02/21	59	NS	NS	0	0	6.32	NO	NO
2730	F	07/02/69	Breast	8	11/09/21	52	YES	NS	0	0	1.26	YES	YES
2757	M	05/08/60	Colorectal	1	11/19/21	61	YES	YES	0	0	0.0	NO	YES
2773	F	05/07/57	Ovary	2	01/05/22	64	YES	NO	25	0	5.06	YES	NO
2800	F	10/04/50	Colorectal	0	12/28/21	71	NS	NS	9	53	2.53	YES	NO
2802	F	01/21/58	Colorectal	0	12/31/21	63	NS	NS	11	15	1.26	YES	NO
2803	M	09/08/48	Stomach	3	12/17/21	73	NS	NS	5	0	3.79	NO	NO
2876	M	06/08/76	Other	1	06/07/22	45	YES	NO	16	0	2.53	NO	YES
2902	M	08/11/56	Lung	3	01/13/22	65	YES	NO	17	72	20.23	YES	NO
2955	F	09/10/68	Lung	0	01/14/22	53	YES	YES	1	0	15.17	NO	NO

Supplementary Table S2

Mutations characteristics

Liquid biopsy-only mutations											
ID	Collection date ? (#1)	Received date ? (#1)	**Gene ? (#1)**	**Alteration (#1)**	**Chromosome: position**	**Transcript ?**	**Strand ?**	**Percent read ?**	**Functional effect ?**	**Depth ?**	**CDS effect ?**
12	06/30/21	07/06/21	TP53	R282W	chr17:7577094	NM_000546	-	0.11	missense	7982	844C>T
28	04/07/21	04/09/21	TP53	N131I	chr17:7578538	NM_000546	-	0.25	missense	4484	392A>T
28	04/07/21	04/09/21	CHEK2	"splice site 319 1G>A"	chr22:29130390	NM_007194	-	0.8	splice	2367	319 1G>A
29	03/18/21	03/22/21	TP53	N131del	chr17:7578534	NM_000546	-	0.25	nonframeshift	5889	393_395delCA A
29	03/18/21	03/22/21	TP53	I195T	chr17:7578265	NM_000546	-	0.22	missense	5875	584T>C
29	03/18/21	03/22/21	BRCA2	R2888H	chr13:32950837	NM_000059	+	0.24	missense	6610	8663G>A
33	04/08/21	04/09/21	TP53	P177L	chr17:7578400	NM_000546	-	0.15	missense	6479	530C>T
36	04/20/21	04/23/21	TP53	Y208*	chr17:7576558	NM_001126117	-	3.05	nonsense	2036	624C>G
38	05/07/21	05/10/21	TP53	E336*	chr17:7574021	NM_000546	-	5.27	nonsense	5335	1006G>T
38	05/07/21	05/10/21	CHEK2	D296fs*8	chr22:29099515	NM_007194	-	2.2	frameshift	2365	885delA
39	04/02/21	04/08/21	TP53	"splice site 559 1G>T"	chr17:7578370	NM_000546	-	1.47	splice	3055	559 1G>T
41	04/02/21	04/08/21	TP53	R175H	chr17:7578406	NM_000546	-	0.38	missense	2126	524G>A
41	12/14/21	12/17/21	TP53	R175H	chr17:7578406	NM_000546	-	0.36	missense	1932	524G>A
57	04/01/21	04/05/21	TP53	R273C	chr17:7577121	NM_000546	-	60.83	missense	3347	817C>T
57	04/01/21	04/05/21	TP53	V173M	chr17:7578413	NM_000546	-	5.65	missense	3966	517G>A
57	04/01/21	04/05/21	PALB2	Q260*	chr16:23647089	NM_024675	-	0.13	nonsense	6189	778C>T
57	04/01/21	04/05/21	PALB2	R1086*	chr16:23619279	NM_024675	-	0.53	nonsense	5837	3256C>T
57	04/01/21	04/05/21	FANCG	A607T	chr9:35074155	NM_004629	-	0.39	missense	1275	1819G>A
57	04/01/21	04/05/21	ATR	I774fs*5	chr3:142274739	NM_001184	-	33.68	frameshift	3857	2320delA
60	04/01/21	04/05/21	TP53	R213P	chr17:7578211	NM_000546	-	0.32	missense	3792	638G>C
80	04/08/21	04/12/21	ATM	L2945fs*10	chr11:10822558 3	NM_000051	+	0.2	frameshift	5531	8833_8834delC T
86	04/08/21	04/12/21	BRCA2	W2830*	chr13:32945095	NM_000059	+	0.3	nonsense	3707	8490G>A
86	04/08/21	04/12/21	ATR	W2565*	chr3:142172036	NM_001184	-	0.23	nonsense	2598	7695G>A

87	04/08/21	04/12/21	TP53	"splice site 993 1G>T"	chr17:7576852	NM_000546	-	4.14	splice	6940	993 1G>T
87	04/08/21	04/12/21	ATM	G2891D	chr11:108224493	NM_000051	+	0.26	missense	4556	8672G>A
89	04/08/21	04/12/21	ATM	G2891D	chr11:108224493	NM_000051	+	0.72	missense	5152	8672G>A
90	04/08/21	04/12/21	TP53	Q144R	chr17:7578499	NM_000546	-	0.34	missense	2345	431A>G
90	04/08/21	04/12/21	TP53	M237I	chr17:7577570	NM_000546	-	1.23	missense	1793	711G>A
90	04/08/21	04/12/21	ATM	R3008C	chr11:108236086	NM_000051	+	0.4	missense	2250	9022C>T
90	04/08/21	04/12/21	ATM	L2445P	chr11:108200967	NM_000051	+	0.55	missense	2191	7334T>C
93	04/09/21	04/12/21	TP53	F113V	chr17:7579350	NM_000546	-	0.27	missense	4080	337T>G
93	04/09/21	04/12/21	TP53	E258D	chr17:7577507	NM_000546	-	0.46	missense	4359	774A>C
93	04/09/21	04/12/21	TP53	L289P	chr17:7577072	NM_000546	-	0.93	missense	6148	866T>C
99	04/12/21	04/15/21	TP53	R249G	chr17:7577536	NM_000546	-	0.12	missense	6070	745A>G
101	04/09/21	04/12/21	ATM	E2294fs*13	chr11:108196856	NM_000051	+	0.46	frameshift	2387	6880_6889delGAAGAAGCAC
114	04/14/21	04/16/21	TP53	N131S	chr17:7578538	NM_000546	-	0.16	missense	6064	392A>G
114	04/14/21	04/16/21	CHEK2	Y404C	chr22:29091746	NM_007194	-	0.19	missense	3600	1211A>G
114	04/14/21	04/16/21	CHEK2	E528fs*38	chr22:29083933	NM_007194	-	0.2	frameshift	3531	1583delA
118	04/15/21	04/16/21	TP53	G279E	chr17:7577102	NM_000546	-	0.24	missense	2965	836G>A
118	04/15/21	04/16/21	CHEK2	Q11*	chr22:29130679	NM_007194	-	15.96	nonsense	2224	31C>T
124	03/16/21	03/19/21	TP53	C275F	chr17:7577114	NM_000546	-	0.27	missense	2245	824G>T
127	02/09/21	02/12/21	TP53	R342*	chr17:7574003	NM_000546	-	6.7	nonsense	5541	1024C>T
135	03/26/21	03/31/21	TP53	Y205C	chr17:7578235	NM_000546	-	0.38	missense	3199	614A>G
135	07/26/21	07/29/21	TP53	Y205C	chr17:7578235	NM_000546	-	0.67	missense	3591	614A>G
135	01/13/22	01/14/22	TP53	Y205C	chr17:7578235	NM_000546	-	0.76	missense	3411	614A>G
140	03/23/21	03/26/21	TP53	A161T	chr17:7578449	NM_000546	-	0.48	missense	6413	481G>A
140	03/23/21	03/26/21	TP53	R280S	chr17:7577098	NM_000546	-	0.34	missense	7013	840A>T
145	04/15/21	04/19/21	TP53	H179D	chr17:7578395	NM_000546	-	1.49	missense	3614	535C>G
147	01/15/21	01/18/21	TP53	Y163C	chr17:7578442	NM_000546	-	2.46	missense	5043	488A>G
147	01/15/21	01/18/21	ATM	R337C	chr11:108117798	NM_000051	+	0.24	missense	2921	1009C>T
147	01/15/21	01/18/21	ATM	S2860del	chr11:108216628	NM_000051	+	0.48	nonframeshift	3145	8578_8580delTCT

147	01/15/21	01/18/21	ATM	N2985fs*21	chr11:108235912	NM_000051	+	0.19	frameshift	4763	8955delT
147	01/15/21	01/18/21	ATM	R1898*	chr11:108178641	NM_000051	+	4.7	nonsense	4217	5692C>T
153	03/22/21	03/24/21	TP53	T125M	chr17:7579313	NM_000546	-	0.27	missense	3385	374C>T
153	03/22/21	03/24/21	TP53	Y220C	chr17:7578190	NM_000546	-	0.19	missense	4209	659A>G
155	05/21/21	05/25/21	TP53	"splice site 1101-1G>A"	chr17:7573009	NM_000546	-	0.93	splice	3021	1101-1G>A
173	11/25/21	11/26/21	TP53	M237I	chr17:7577570	NM_000546	-	0.5	missense	2212	711G>A
183	02/09/21	02/12/21	TP53	"splice site 919 1G>A"	chr17:7577018	NM_000546	-	0.71	splice	6337	919 1G>A
183	02/09/21	02/12/21	BRCA2	N1784fs*7	chr13:32913842	NM_000059	+	0.26	frameshift	3850	5351delA
184	02/01/21	02/03/21	TP53	"splice site 375G>T"	chr17:7579312	NM_000546	-	2.5	splice	3556	375G>T
184	02/01/21	02/03/21	CHEK2	T383P	chr22:29091810	NM_007194	-	0.69	missense	3906	1147A>C
186	01/19/21	01/22/21	TP53	"splice site 783-1G>T"	chr17:7577156	NM_000546	-	0.25	splice	3584	783-1G>T
186	01/19/21	01/22/21	CHEK2	F447fs*22	chr22:29091149	NM_007194	-	0.27	frameshift	2250	1340delT
187	04/14/22	04/20/22	TP53	Y220C	chr17:7578190	NM_000546	-	0.32	missense	2476	659A>G
190	01/26/21	01/29/21	TP53	I195T	chr17:7578265	NM_000546	-	0.14	missense	5781	584T>C
190	01/26/21	01/29/21	NBN	D95fs*14	chr8:90993639	NM_002485	-	1.24	frameshift	404	283delG
190	01/26/21	01/29/21	MSH6	F1088fs*5	chr2:48030647	NM_000179	+	6.69	frameshift	972	3261_3262insC
190	01/26/21	01/29/21	BRCA1	K339fs*2	chr17:41246531	NM_007294	-	0.78	frameshift	7407	1016delA
191	02/10/21	02/13/21	TP53	R248Q	chr17:7577538	NM_000546	-	0.26	missense	3825	743G>A
191	02/10/21	02/13/21	CHEK2	"""splice site 1096-2A>T"""	chr22:29091863	NM_007194	-	0.3	splice	2000	1096-2A>T
195	02/01/21	02/04/21	TP53	"splice site 920-1G>A"	chr17:7576927	NM_000546	-	0.81	splice	2851	920-1G>A
200	11/22/21	11/24/21	TP53	V143A	chr17:7578502	NM_000546	-	0.1	missense	6062	428T>C
200	01/29/21	02/01/21	CHEK2	L333*	chr22:29095836	NM_007194	-	0.17	nonsense	5418	998T>A
200	11/22/21	11/24/21	CHEK2	L333*	chr22:29095836	NM_007194	-	0.18	nonsense	4472	998T>A
201	09/23/21	09/27/21	CHEK2	S210fs*3	chr22:29115432	NM_007194	-	0.3	frameshift	1336	629_633delCAGTT
201	02/25/21	03/01/21	CHEK2	S210fs*3	chr22:29115432	NM_007194	-	0.75	frameshift	1868	629_633delCAGTT
202	01/21/21	01/25/21	TP53	K132N	chr17:7578534	NM_000546	-	1.35	missense	6512	396G>C
206	01/20/21	01/22/21	TP53	"splice site 375G>A"	chr17:7579312	NM_000546	-	12.45	splice	2650	375G>A

206	01/20/21	01/22/21	ATR	I774fs*5	chr3:142274739	NM_001184	-	1.96	frameshift	3417	2320delA
213	01/26/21	01/28/21	ATR	"splice site 4852 2T>C"	chr3:142231100	NM_001184	-	0.19	splice	2054	4852 2T>C
214	10/04/21	10/05/21	TP53	E285K	chr17:7577085	NM_000546	-	1.21	missense	5209	853G>A
214	10/04/21	10/05/21	TP53	V173L	chr17:7578413	NM_000546	-	0.14	missense	4886	517G>T
214	01/22/21	01/25/21	TP53	E285K	chr17:7577085	NM_000546	-	0.24	missense	2106	853G>A
228	01/26/21	01/29/21	TP53	"splice site 994-1G>C"	chr17:7574034	NM_000546	-	4.18	splice	3471	994-1G>C
229	01/20/21	01/22/21	TP53	R175H	chr17:7578406	NM_000546	-	0.22	missense	3156	524G>A
232	02/01/21	02/04/21	TP53	"splice site 376-2A>T"	chr17:7578556	NM_000546	-	3.32	splice	2259	376-2A>T
233	02/05/21	02/08/21	TP53	I195N	chr17:7578265	NM_000546	-	7.9	missense	2001	584T>A
233	12/10/21	12/14/21	TP53	I195N	chr17:7578265	NM_000546	-	42.2	missense	5995	584T>A
233	02/05/21	02/08/21	CHEK2	Q330*	chr22:29095846	NM_007194	-	0.26	nonsense	1920	988C>T
233	12/10/21	12/14/21	BRCA1	splice site 4093_4096 10del14	chr17:41243441	NM_007294	-	0.64	splice	9458	4093_4096 10del14
236	02/15/21	02/17/21	CHEK2	W93fs*17	chr22:29130433	NM_007194	-	0.5	frameshift	4406	276delC
236	02/15/21	02/17/21	CHEK2	"splice site 1095 1G>A"	chr22:29092888	NM_007194	-	0.81	splice	3087	1095 1G>A
236	02/15/21	02/17/21	ATM	P1607fs*2	chr11:10816569 6	NM_000051	+	0.18	frameshift	4972	4820delC
240	02/23/21	02/25/21	TP53	F113V	chr17:7579350	NM_000546	-	1.62	missense	3522	337T>G
242	12/17/20	12/19/20	TP53	N131S	chr17:7578538	NM_000546	-	0.56	missense	4444	392A>G
242	12/17/20	12/19/20	ATR	F222fs*11	chr3:142281577	NM_001184	-	0.6	frameshift	4318	666delT
254	02/11/21	02/22/21	TP53	"splice site 97-2A>T"	chr17:7579592	NM_000546	-	0.11	splice	10172	97-2A>T
256	02/02/21	02/04/21	CHEK2	E107fs*3	chr22:29130390	NM_007194	-	0.23	frameshift	2626	319delG
259	01/20/21	01/22/21	TP53	Y220N	chr17:7578191	NM_000546	-	0.18	missense	4887	658T>A
262	02/04/21	02/08/21	TP53	M246T	chr17:7577544	NM_000546	-	26.59	missense	3705	737T>C
264	03/30/21	04/01/21	TP53	H214R	chr17:7578208	NM_000546	-	7.57	missense	2749	641A>G
265	09/24/21	09/29/21	ATR	"splice site 4267-2A>G"	chr3:142238628	NM_001184	-	0.2	splice	4452	4267-2A>G
266	01/20/21	01/22/21	CHEK2	I157T	chr22:29121087	NM_007194	-	0.19	missense	2105	470T>C
276	02/17/21	02/20/21	TP53	R306*	chr17:7577022	NM_000546	-	0.33	nonsense	2415	916C>T
276	02/17/21	02/20/21	TP53	L265P	chr17:7577144	NM_000546	-	0.44	missense	2291	794T>C

276	02/17/21	02/20/21	TP53	Y234S	chr17:7577576	NM_000546	-	0.37	missense	1900	701_705ACAA C>CCAAT
276	02/17/21	02/20/21	CHEK2	K373fs*22	chr22:29091840	NM_007194	-	0.28	frameshift	1423	1116_1117insC
276	02/17/21	02/20/21	CHEK2	Y445*	chr22:29091155	NM_007194	-	0.64	nonsense	1707	1335C>G
276	02/17/21	02/20/21	CHEK2	Y156*	chr22:29121089	NM_007194	-	0.34	nonsense	1773	468C>A
280	01/28/21	02/01/21	TP53	A159D	chr17:7578454	NM_000546	-	0.1	missense	6180	476C>A
283	03/30/21	04/01/21	TP53	Y236C	chr17:7577574	NM_000546	-	0.18	missense	3370	707A>G
283	03/30/21	04/01/21	TP53	"splice site 783-1G>A"	chr17:7577156	NM_000546	-	0.51	splice	3502	783-1G>A
289	01/26/21	01/28/21	TP53	"splice site 919 1G>A"	chr17:7577018	NM_000546	-	9.53	splice	3567	919 1G>A
289	01/26/21	01/28/21	CHEK2	"splice site 792 2T>C"	chr22:29107895	NM_007194	-	0.48	splice	2307	792 2T>C
295	02/11/21	02/15/21	TP53	N239D	chr17:7577566	NM_000546	-	0.71	missense	2672	715A>G
295	02/11/21	02/15/21	BRCA1	M1I	chr17:41276111	NM_007294	-	0.79	nonsense	2397	3G>T
295	02/11/21	02/15/21	ATM	L2445P	chr11:108200967	NM_000051	+	0.22	missense	4080	7334T>C
295	02/11/21	02/15/21	ATM	N1983fs*7	chr11:108183167	NM_000051	+	0.18	frameshift	3865	5948_5951ATA C>GTA
295	02/11/21	02/15/21	ATM	M660fs*24	chr11:108124619	NM_000051	+	0.11	frameshift	5441	1978_2035del58
296	02/05/21	02/09/21	BRIP1	"splice site 2906-1G>C"	chr17:59761502	NM_032043	-	3.98	splice	854	2906-1G>C
304	02/09/21	02/12/21	TP53	K382fs*40	chr17:7572962	NM_000546	-	0.59	frameshift	4760	1146delA
305	02/26/21	03/01/21	TP53	P190L	chr17:7578280	NM_000546	-	0.46	missense	4740	569C>T
305	02/26/21	03/01/21	TP53	I195T	chr17:7578265	NM_000546	-	1.46	missense	5126	584T>C
305	02/26/21	03/01/21	ATRX	Q2194*	chrX:76813041	NM_000489	-	1.79	nonsense	952	6580C>T
314	02/02/21	02/05/21	TP53	G154V	chr17:7578469	NM_000546	-	0.11	missense	8565	461G>T
314	02/02/21	02/05/21	TP53	V157F	chr17:7578461	NM_000546	-	0.23	missense	8605	469G>T
314	02/02/21	02/05/21	TP53	E349fs*21	chr17:7573979	NM_000546	-	0.37	frameshift	7108	1047delA
320	06/03/21	06/09/21	TP53	V157F	chr17:7578461	NM_000546	-	0.17	missense	7521	469G>T
320	06/03/21	06/09/21	TP53	N239S	chr17:7577565	NM_000546	-	0.24	missense	5454	716A>G
320	01/18/21	01/20/21	TP53	N239S	chr17:7577565	NM_000546	-	0.18	missense	3419	716A>G
320	06/03/21	06/09/21	ATM	G2891D	chr11:108224493	NM_000051	+	1.91	missense	4337	8672G>A
320	01/18/21	01/20/21	ATM	G2891D	chr11:108224493	NM_000051	+	1.63	missense	3731	8672G>A

322	03/11/21	03/15/21	CHEK1	T226fs*14	chr11:125505385	NM_001274	+	5.7	frameshift	825	676delA
322	03/11/21	03/15/21	ATR	I774fs*5	chr3:142274739	NM_001184	-	3.7	frameshift	2485	2320delA
324	02/12/21	02/19/21	TP53	R273H	chr17:7577120	NM_000546	-	0.29	missense	5205	818G>A
324	02/12/21	02/19/21	ATM	E73fs*26	chr11:108099935	NM_000051	+	0.3	frameshift	3725	217_218delGA
329	02/11/21	02/15/21	TP53	Y234C	chr17:7577580	NM_000546	-	0.14	missense	4387	701A>G
332	03/12/21	03/18/21	TP53	R248W	chr17:7577539	NM_000546	-	0.38	missense	2894	742C>T
332	03/12/21	03/18/21	TP53	"splice site 375 1G>C"	chr17:7579311	NM_000546	-	57.27	splice	2406	375 1G>C
333	03/04/21	03/08/21	TP53	Y234F	chr17:7577580	NM_000546	-	0.25	missense	4053	701A>T
336	02/09/21	02/13/21	TP53	H179R	chr17:7578394	NM_000546	-	0.31	missense	4238	536A>G
342	03/04/21	03/08/21	ATM	V2577fs*7	chr11:108202705	NM_000051	+	0.23	frameshift	2578	7730delT
348	03/24/21	03/25/21	CHEK2	R523fs*2	chr22:29083949	NM_007194	-	0.33	frameshift	2455	1567_1568insC
348	03/24/21	03/25/21	CHEK2	P522fs*3	chr22:29083953	NM_007194	-	1.76	frameshift	2442	1563_1564insG
351	01/22/21	01/25/21	BRCA2	F1787fs*4	chr13:32913853	NM_000059	+	0.19	frameshift	5156	5361_5363TTC>AA
355	08/23/21	08/25/21	TP53	E287*	chr17:7577079	NM_000546	-	0.16	nonsense	3174	859G>T
355	08/23/21	08/25/21	TP53	N131del	chr17:7578534	NM_000546	-	0.25	nonframeshift	2844	393_395delCAA
358	03/09/21	03/11/21	TP53	Y205H	chr17:7578236	NM_000546	-	4.32	missense	3011	613T>C
359	03/09/21	03/12/21	BRCA1	K325*	chr17:41246575	NM_007294	-	4.45	nonsense	9302	973A>T
360	03/04/21	03/06/21	PMS2	"splice site 164-1G>T"	chr7:6043690	NM_000535	-	4.96	splice	706	164-1G>T
360	03/04/21	03/06/21	MSH2	G204fs*10	chr2:47637476	NM_000251	+	0.84	frameshift	1305	611delG
360	03/04/21	03/06/21	MLH1	G101D	chr3:37042540	NM_000249	+	0.39	missense	1037	302G>A
360	03/04/21	03/06/21	ATR	W1800*	chr3:142217597	NM_001184	-	0.45	nonsense	2435	5400G>A
360	03/04/21	03/06/21	ATM	W1461*	chr11:108160475	NM_000051	+	0.31	nonsense	2226	4383G>A
362	02/08/21	02/10/21	TP53	"splice site 672 1G>A"	chr17:7578176	NM_000546	-	0.29	splice	2054	672 1G>A
362	02/08/21	02/10/21	CHEK2	Y156fs*1	chr22:29121089	NM_007194	-	1.24	frameshift	1937	467_468insA
366	01/25/21	01/28/21	TP53	"splice site 672G>A"	chr17:7578177	NM_000546	-	10.2	splice	3519	672G>A
366	01/25/21	01/28/21	TP53	D281V	chr17:7577096	NM_000546	-	0.24	missense	5399	842A>T
366	01/25/21	01/28/21	TP53	D281Y	chr17:7577097	NM_000546	-	0.26	missense	5349	841G>T

366	01/25/21	01/28/21	TP53	C275Y	chr17:7577114	NM_000546	-	0.24	missense	4555	824G>A
369	02/09/21	02/12/21	ATR	"splice site 2079-2A>G"	chr3:142274983	NM_001184	-	0.21	splice	4760	2079-2A>G
377	12/15/20	12/18/20	TP53	V143G	chr17:7578502	NM_000546	-	0.17	missense	4133	428T>G
377	12/15/20	12/18/20	FANCC	Q357fs*12	chr9:97879600	NM_000136	-	0.69	frameshift	1449	1068delT
380	12/23/20	12/24/20	ATM	G2891D	chr11:10822449 3	NM_000051	+	0.88	missense	3185	8672G>A
381	12/17/20	12/19/20	TP53	P34fs*2	chr17:7579562	NM_000546	-	26.91	frameshift	6619	100_124del25
383	12/22/20	12/24/20	TP53	N239S	chr17:7577565	NM_000546	-	0.36	missense	3352	716A>G
383	12/22/20	12/24/20	TP53	H193R	chr17:7578271	NM_000546	-	0.73	missense	3839	578A>G
383	12/22/20	12/24/20	TP53	E258K	chr17:7577509	NM_000546	-	0.49	missense	3290	772G>A
383	12/22/20	12/24/20	TP53	Y205C	chr17:7578235	NM_000546	-	0.38	missense	3401	614A>G
383	12/22/20	12/24/20	MLH1	T117M	chr3:37045935	NM_000249	+	0.67	missense	902	350C>T
383	12/22/20	12/24/20	CHEK1	W404*	chr11:12551451 7	NM_001274	+	0.64	nonsense	778	1212G>A
383	12/22/20	12/24/20	ATM	"splice site 5178-1G>A"	chr11:10817237 4	NM_000051	+	0.57	splice	2641	5178-1G>A
398	01/07/21	01/11/21	TP53	Q317*	chr17:7576897	NM_000546	-	0.2	nonsense	8091	949C>T
401	01/12/21	01/14/21	TP53	"splice site 559 1G>A"	chr17:7578370	NM_000546	-	25.87	splice	8507	559 1G>A
414	01/14/21	01/16/21	TP53	C242S	chr17:7577556	NM_000546	-	0.2	missense	3439	725G>C
414	01/14/21	01/16/21	ATM	E73fs*26	chr11:10809993 5	NM_000051	+	0.23	frameshift	3040	217_218delGA
420	01/19/21	01/22/21	TP53	"splice site 994-17_1003del27"	chr17:7576647	NM_001126114	-	0.16	splice	6783	994-17_1003del27
425	01/19/21	01/22/21	TP53	R273C	chr17:7577121	NM_000546	-	14.33	missense	4369	817C>T
444	01/25/21	01/28/21	TP53	"splice site 375G>A"	chr17:7579312	NM_000546	-	20.06	splice	2458	375G>A
444	01/25/21	01/28/21	ATM	R2849*	chr11:10821659 6	NM_000051	+	0.31	nonsense	3575	8545C>T
446	01/25/21	01/28/21	RAD51C	K84fs*8	chr17:56772395	NM_058216	+	1.03	frameshift	1360	249_250insGG ATATCTAAA GCAGAAGCC TTAGAAACTC TGCAAATTAT CAGAAGAGA ATGTCTCACA AATAAACCA AGATATGCT GGTACATCTG AGTCACACA AG

446	01/25/21	01/28/21	ATM	G2891D	chr11:108224493	NM_000051	+	0.14	missense	2912	8672G>A
461	01/27/21	01/30/21	MLH1	"splice site 454-2A>G"	chr3:37050303	NM_000249	+	47.68	splice	646	454-2A>G
462	01/28/21	02/01/21	BRCA2	N863fs*11	chr13:32911079	NM_000059	+	0.6	frameshift	5378	2588delA
474	01/28/21	02/01/21	CHEK2	T383A	chr22:29091810	NM_007194	-	0.2	missense	4046	1147A>G
484	02/01/21	02/04/21	TP53	V73fs*50	chr17:7579470	NM_000546	-	0.76	frameshift	9215	216delC
484	08/06/21	08/12/21	TP53	V73fs*50	chr17:7579470	NM_000546	-	0.86	frameshift	7183	216delC
484	08/06/21	08/12/21	PALB2	N280fs*8	chr16:23647027	NM_024675	-	0.17	frameshift	5384	839delA
484	02/01/21	02/04/21	NBN	R466fs*18	chr8:90967511	NM_002485	-	7.38	frameshift	569	1396delA
484	08/06/21	08/12/21	NBN	R466fs*18	chr8:90967511	NM_002485	-	8.2	frameshift	671	1396delA
484	02/01/21	02/04/21	CHEK2	N154fs*7	chr22:29121095	NM_007194	-	0.51	frameshift	3950	461delA
484	08/06/21	08/12/21	CHEK2	N154fs*7	chr22:29121095	NM_007194	-	1.52	frameshift	3300	461delA
484	02/01/21	02/04/21	BRCA2	Q1429fs*9	chr13:32912776	NM_000059	+	9.34	frameshift	5620	4284_4285insT
484	08/06/21	08/12/21	BRCA2	Q1429fs*9	chr13:32912776	NM_000059	+	9	frameshift	4879	4284_4285insT
494	02/03/21	02/05/21	TP53	C176R	chr17:7578404	NM_000546	-	0.09	missense	8138	526T>C
526	12/03/21	12/08/21	TP53	R158C	chr17:7578458	NM_000546	-	0.29	missense	5905	472C>T
526	12/03/21	12/08/21	BRCA1	E699_A1279del	chr17:41243710	NM_007294	-	0.66	nonframeshift	11885	2095_3837del1743
526	12/03/21	12/08/21	BRCA1	G949_L1216del	chr17:41243899	NM_007294	-	0.27	nonframeshift	11316	2845_3648del804
526	12/03/21	12/08/21	BRCA1	R1203W	chr17:41243939	NM_007294	-	12.16	missense	6379	3607_3609CGA>TGG
526	12/03/21	12/08/21	BRCA1	Q1200_A1206del	chr17:41243930	NM_007294	-	0.42	nonframeshift	7863	3597_3617del21
526	12/03/21	12/08/21	BRCA1	L1198_N1215>F	chr17:41243903	NM_007294	-	8.56	nonframeshift	8997	3594_3644del51
536	02/09/21	02/12/21	TP53	"splice site 376-1G>A"	chr17:7578555	NM_000546	-	0.17	splice	3514	376-1G>A
536	12/21/21	12/27/21	TP53	C238W	chr17:7577567	NM_000546	-	0.12	missense	4914	714T>G
536	02/09/21	02/12/21	CHEK2	I157T	chr22:29121087	NM_007194	-	0.5	missense	2189	470T>C
536	02/09/21	02/12/21	CHEK2	R137*	chr22:29121266	NM_007194	-	0.3	nonsense	1983	409C>T
536	02/09/21	02/12/21	CHEK2	M1I	chr22:29130707	NM_007194	-	0.22	nonsense	2784	3G>A
536	12/21/21	12/27/21	CHEK2	I157T	chr22:29121087	NM_007194	-	0.15	missense	3332	470T>C
536	12/21/21	12/27/21	CHEK2	R137*	chr22:29121266	NM_007194	-	0.33	nonsense	3042	409C>T
536	02/09/21	02/12/21	BRCA2	N2146fs*21	chr13:32914928	NM_000059	+	1.16	frameshift	3618	6437_6440delATCA

536	12/21/21	12/27/21	BRCA2	N2146fs*21	chr13:32914928	NM_000059		0.86	frameshift	5586	6437_6440delA TCA
541	02/10/21	02/13/21	TP53	R248Q	chr17:7577538	NM_000546	-	1.12	missense	4207	743G>A
541	02/10/21	02/13/21	TP53	Y220C	chr17:7578190	NM_000546	-	3.87	missense	3643	659A>G
544	02/10/21	02/13/21	TP53	A276P	chr17:7577112	NM_000546	-	1.51	missense	5160	826G>C
544	02/10/21	02/13/21	TP53	N239S	chr17:7577565	NM_000546	-	0.31	missense	4154	716A>G
544	02/10/21	02/13/21	TP53	S215R	chr17:7578204	NM_000546	-	1.62	missense	3950	645T>G
544	02/10/21	02/13/21	TP53	M246V	chr17:7577545	NM_000546	-	0.2	missense	4571	736A>G
544	02/10/21	02/13/21	TP53	I195T	chr17:7578265	NM_000546	-	0.48	missense	5017	584T>C
544	02/10/21	02/13/21	TP53	L265P	chr17:7577144	NM_000546	-	0.15	missense	4710	794T>C
544	02/10/21	02/13/21	CHEK2	Y404C	chr22:29091746	NM_007194	-	0.5	missense	2984	1211A>G
544	02/10/21	02/13/21	CHEK2	"splice site 909-1G>A"	chr22:29095926	NM_007194	-	0.43	splice	3700	909-1G>A
544	02/10/21	02/13/21	CHEK2	"splice site 1096-1G>A"	chr22:29091862	NM_007194	-	2.58	splice	2213	1096-1G>A
544	02/10/21	02/13/21	ATM	R337C	chr11:108117798	NM_000051	+	1.51	missense	3509	1009C>T
544	02/10/21	02/13/21	ATM	"splice site 662 1G>T"	chr11:108114846	NM_000051	+	1.88	splice	3827	662 1G>T
544	02/10/21	02/13/21	ATM	Y2371fs*1	chr11:108199770	NM_000051	+	0.27	frameshift	4101	7112_7113insA
566	02/15/21	02/18/21	TP53	R282W	chr17:7577094	NM_000546	-	0.62	missense	6899	844C>T
582	02/18/21	02/20/21	TP53	H193R	chr17:7578271	NM_000546	-	0.15	missense	3324	578A>G
583	02/18/21	02/23/21	TP53	R273H	chr17:7577120	NM_000546	-	0.39	missense	2811	818G>A
589	02/18/21	02/24/21	TP53	"splice site 376-1G>A"	chr17:7578555	NM_000546	-	0.3	splice	6668	376-1G>A
627	02/24/21	03/01/21	TP53	F134C	chr17:7578529	NM_000546	-	0.15	missense	5845	401T>G
627	02/24/21	03/01/21	TP53	R280G	chr17:7577100	NM_000546	-	0.11	missense	6380	838A>G
627	02/24/21	03/01/21	TP53	T155I	chr17:7578466	NM_000546	-	0.14	missense	5788	464C>T
627	02/24/21	03/01/21	TP53	I195T	chr17:7578265	NM_000546	-	1	missense	5788	584T>C
627	02/24/21	03/01/21	ATM	W1058*	chr11:108143469	NM_000051	+	0.56	nonsense	6663	3174G>A
629	02/25/21	03/01/21	ATM	Q2028*	chr11:108186625	NM_000051	+	0.3	nonsense	5761	6082C>T
633	02/26/21	03/01/21	TP53	L194R	chr17:7578268	NM_000546	-	0.29	missense	6232	581T>G
633	02/26/21	03/01/21	TP53	R273H	chr17:7577120	NM_000546	-	0.51	missense	6526	818G>A
647	03/02/21	03/05/21	TP53	G245D	chr17:7577547	NM_000546	-	1.7	missense	1887	734G>A

671	03/04/21	03/09/21	TP53	C238S	chr17:7577568	NM_000546	-	0.25	missense	4337	713G>C
671	03/04/21	03/09/21	TP53	G245S	chr17:7577548	NM_000546	-	0.3	missense	4604	733G>A
671	03/04/21	03/09/21	BRCA1	S1320fs*10	chr17:41243589	NM_007294	-	0.21	frameshift	6225	3958_3959insT
687	03/08/21	03/11/21	TP53	H179D	chr17:7578395	NM_000546	-	14.35	missense	5506	535C>G
687	03/08/21	03/11/21	TP53	E349*	chr17:7573982	NM_000546	-	47.31	nonsense	4394	1045G>T
687	03/08/21	03/11/21	TP53	L348F	chr17:7573983	NM_000546	-	47.31	missense	4394	1044G>T
687	03/08/21	03/11/21	FANCL	"splice site 904-2A>G"	chr2:58388775	NM_018062	-	4.52	splice	774	904-2A>G
688	03/08/21	03/11/21	TP53	V216M	chr17:7578203	NM_000546	-	0.23	missense	2622	646G>A
688	03/08/21	03/11/21	TP53	E198*	chr17:7578257	NM_000546	-	0.41	nonsense	3376	592G>T
690	03/09/21	03/10/21	TP53	R282W	chr17:7577094	NM_000546	-	0.28	missense	2816	844C>T
690	01/21/22	01/25/22	TP53	splice site 673-11_676del15	chr17:7577604	NM_000546	-	1.42	splice	4650	673-11_676del15
692	06/01/21	06/09/21	TP53	Y236C	chr17:7577574	NM_000546	-	0.22	missense	3583	707A>G
692	06/01/21	06/09/21	ATM	R337C	chr11:108117798	NM_000051	+	0.24	missense	3302	1009C>T
692	03/09/21	03/11/21	ATM	R337C	chr11:108117798	NM_000051	+	0.28	missense	3167	1009C>T
692	06/14/21	06/18/21	ATM	R337C	chr11:108117798	NM_000051	+	0.2	missense	3584	1009C>T
749	12/23/21	12/29/21	TP53	C176fs*12	chr17:7578404	NM_000546	-	0.12	frameshift	5883	525_526insCCGCCATGGCCATCTACAAGCAGTCACAGCACATGACGGAGGTTGTGAGGCGC
749	03/17/22	03/21/22	TP53	H214L	chr17:7578208	NM_000546	-	0.18	missense	5037	641A>T
749	03/17/22	03/21/22	TP53	C176fs*12	chr17:7578404	NM_000546	-	0.17	frameshift	9668	525_526insCCGCCATGGCCATCTACAAGCAGTCACAGCACATGACGGAGGTTGTGAGGCGC
749	07/01/22	07/07/22	TP53	C176fs*12	chr17:7578404	NM_000546	-	0.24	frameshift	9757	525_526insCCGCCATGGCCATCTACAAGCAGTCACAGCACATGACGGAGGTTGTGAGGCGC
749	07/01/22	07/07/22	TP53	H214L	chr17:7578208	NM_000546	-	0.21	missense	5334	641A>T

757	03/17/21	03/23/21	TP53	E339*	chr17:7574012	NM_000546	-	14.76	nonsense	5564	1015G>T
758	03/18/21	03/22/21	RAD54L	C391fs*1	chr1:46736380	NM_003579	+	30.88	frameshift	1804	1092_1093insC GAGACGCTG CTGCTAGTGA GGCAGACAG GCAGCTAGG AGAGGAGCG GCTGCGGGA GCTCACCAG CATTGTGAAT AGGTAATGA CCTTAAGC
764	03/18/21	03/23/21	TP53	"""splice site 375G>T"""	chr17:7579312	NM_000546	-	34.68	splice	2269	375G>T
764	03/18/21	03/23/21	POLE	E1424D	chr12:13322044 1	NM_006231	-	0.56	missense	1075	4272G>T
764	03/18/21	03/23/21	BRCA2	"""splice site 316 2T>G"""	chr13:32893464	NM_000059	+	48.47	splice	4339	"316 2T>G"
768	03/19/21	03/23/21	TP53	M237I	chr17:7577570	NM_000546	-	0.45	missense	3099	711G>A
768	03/19/21	03/23/21	TP53	C275Y	chr17:7577114	NM_000546	-	0.26	missense	3521	824G>A
768	03/19/21	03/23/21	TP53	R280T	chr17:7577099	NM_000546	-	0.14	missense	4259	839G>C
768	03/19/21	03/23/21	ATM	R23*	chr11:10809841 8	NM_000051	+	0.19	nonsense	3659	67C>T
840	03/30/21	04/01/21	CHEK2	S500fs*14	chr22:29085167	NM_007194	-	2.88	frameshift	208	1497_1498insA CAGAAGATC ATGAAGAGA AAGTTTCAA GATCTTCTG
845	03/31/21	04/05/21	TP53	S241C	chr17:7577559	NM_000546	-	1.05	missense	1716	722C>G
845	03/31/21	04/05/21	ATR	I774fs*3	chr3:142274739	NM_001184	-	4.38	frameshift	707	2320_2321insA
863	04/21/21	04/23/21	TP53	"splice site 994-1G>T"	chr17:7574034	NM_000546	-	5.85	splice	5161	994-1G>T
875	11/03/21	11/05/21	TP53	M237I	chr17:7577570	NM_000546	-	0.31	missense	6042	711G>A
876	05/14/21	05/19/21	TP53	R280G	chr17:7577100	NM_000546	-	0.86	missense	3037	838A>G
876	05/14/21	05/19/21	RAD54L	C391fs*1	chr1:46736380	NM_003579	+	30.05	frameshift	1524	1092_1093insC GAGACGCTG CTGCTAGTGA GGCAGACAG GCAGCTAGG AGAGGAGCG GCTGCGGGA GCTCACCAG CATTGTGAAT AGGTAATGA CCTTAAGC
884	09/16/21	09/20/21	TP53	C176Y	chr17:7578403	NM_000546	-	0.3	missense	3362	527G>A

884	09/16/21	09/20/21	TP53	V143M	chr17:7578503	NM_000546	-	0.24	missense	3283	427G>A
884	10/27/21	10/28/21	TP53	C176Y	chr17:7578403	NM_000546	-	0.22	missense	2778	527G>A
884	09/16/21	09/20/21	ATM	M2405L	chr11:108199871	NM_000051	+	0.16	missense	4479	7213A>C
884	10/27/21	10/28/21	ATM	E2039K	chr11:108186757	NM_000051	+	0.16	missense	3036	6115G>A
884	10/27/21	10/28/21	ATM	M2405L	chr11:108199871	NM_000051	+	0.28	missense	3594	7213A>C
888	04/23/21	04/26/21	CHEK2	Q487*	chr22:29090022	NM_007194	-	0.17	nonsense	4102	1459C>T
898	05/25/21	05/28/21	TP53	R248Q	chr17:7577538	NM_000546	-	0.39	missense	6586	743G>A
898	05/25/21	05/28/21	TP53	Y205D	chr17:7578236	NM_000546	-	0.17	missense	6432	613T>G
901	04/20/21	04/23/21	ATM	"splice site 8851-1G>T"	chr11:108235808	NM_000051	+	0.15	splice	4006	8851-1G>T
902	04/21/21	04/23/21	TP53	A276_C277del	chr17:7577106	NM_000546	-	0.14	nonframeshift	6310	826_831delGCCTGT
912	04/21/21	04/23/21	CHEK2	P90fs*20	chr22:29130440	NM_007194	-	0.7	frameshift	5752	269delC
917	05/05/21	05/08/21	CHEK2	S28fs*49	chr22:29130627	NM_007194	-	0.24	frameshift	2895	82_83insT
918	10/21/21	10/22/21	TP53	P151T	chr17:7578479	NM_000546	-	0.16	missense	7917	450_451AC>CA
929	04/20/21	04/23/21	ATM	"splice site 8152-1G>A"	chr11:108206571	NM_000051	+	0.09	splice	10707	8152-1G>A
940	04/22/21	04/26/21	TP53	Y205C	chr17:7578235	NM_000546	-	0.25	missense	4038	614A>G
962	04/16/21	04/20/21	TP53	G245A	chr17:7577547	NM_000546	-	0.21	missense	4274	734G>C
962	04/16/21	04/20/21	CHEK2	T387A	chr22:29091798	NM_007194	-	1.69	missense	3600	1159A>G
962	04/16/21	04/20/21	BRCA2	"splice site 9257-2A>G"	chr13:32968824	NM_000059	+	61.06	splice	3752	9257-2A>G
971	05/12/21	05/17/21	TP53	"splice site 782 1G>T"	chr17:7577498	NM_000546	-	1.44	splice	4945	782 1G>T
978	04/28/21	04/29/21	TP53	V272M	chr17:7577124	NM_000546	-	0.38	missense	4516	814G>A
978	04/28/21	04/29/21	TP53	"splice site 994-1G>A"	chr17:7574034	NM_000546	-	2.14	splice	3737	994-1G>A
978	04/28/21	04/29/21	ATM	G2891D	chr11:108224493	NM_000051	+	0.17	missense	4142	8672G>A
983	06/16/21	06/21/21	TP53	C277Y	chr17:7577108	NM_000546	-	0.19	missense	6677	830G>A
983	06/16/21	06/21/21	TP53	C275Y	chr17:7577114	NM_000546	-	0.34	missense	6441	824G>A
983	06/16/21	06/21/21	TP53	P152fs*18	chr17:7578474	NM_000546	-	2.02	frameshift	6789	455delC
983	06/16/21	06/21/21	TP53	Y220C	chr17:7578190	NM_000546	-	1.03	missense	4875	659A>G
983	06/16/21	06/21/21	TP53	R158H	chr17:7578457	NM_000546	-	1.16	missense	6736	473G>A

983	06/16/21	06/21/21	TP53	Y163D	chr17:7578443	NM_000546	-	0.36	missense	7690	487T>G
983	06/16/21	06/21/21	TP53	V216M	chr17:7578203	NM_000546	-	0.44	missense	4542	646G>A
983	06/16/21	06/21/21	TP53	R249G	chr17:7577536	NM_000546	-	1.71	missense	6215	745A>G
983	06/16/21	06/21/21	TP53	"splice site 559 1G>A"	chr17:7578370	NM_000546	-	0.19	splice	6414	559 1G>A
983	06/16/21	06/21/21	FANCA	S1093fs*24	chr16:89815135	NM_000135	-	1.68	frameshift	1373	3279delC
987	10/26/21	10/28/21	ATM	W2205*	chr11:108196079	NM_000051		0.17	nonsense	7028	6615G>A
1009	04/29/21	05/03/21	TP53	C242F	chr17:7577556	NM_000546	-	2.73	missense	4281	725G>T
1009	04/29/21	05/03/21	CHEK2	D368N	chr22:29091855	NM_007194	-	0.89	missense	2366	1102G>A
1009	04/29/21	05/03/21	BRCA2	A2526fs*2	chr13:32930704	NM_000059	+	1.01	frameshift	5570	7576delG
1013	06/01/21	06/09/21	TP53	"splice site 376-1G>A"	chr17:7578555	NM_000546	-	0.1	splice	5995	376-1G>A
1021	04/29/21	05/03/21	TP53	R273H	chr17:7577120	NM_000546	-	0.24	missense	5806	818G>A
1021	12/08/21	12/09/21	TP53	R273H	chr17:7577120	NM_000546	-	0.19	missense	5854	818G>A
1022	04/28/21	04/30/21	ATM	T1953A	chr11:108180981	NM_000051	+	0.22	missense	6289	5857A>G
1022	04/28/21	04/30/21	ATM	G2891D	chr11:108224493	NM_000051	+	0.13	missense	5995	8672G>A
1022	04/28/21	04/30/21	ATM	C11*	chr11:108098384	NM_000051	+	4.75	nonsense	6396	33C>A
1034	04/29/21	04/30/21	TP53	C242fs*5	chr17:7577557	NM_000546	-	2.65	frameshift	2450	723delC
1034	04/29/21	04/30/21	TP53	E258Q	chr17:7577509	NM_000546	-	0.23	missense	2576	772G>C
1034	04/29/21	04/30/21	TP53	C176S	chr17:7578404	NM_000546	-	0.42	missense	2844	526T>A
1034	04/29/21	04/30/21	TP53	H179Y	chr17:7578395	NM_000546	-	11.2	missense	2562	535C>T
1034	04/29/21	04/30/21	TP53	V216M	chr17:7578203	NM_000546	-	0.27	missense	2234	646G>A
1034	04/29/21	04/30/21	TP53	R282W	chr17:7577094	NM_000546	-	0.23	missense	2556	844C>T
1034	04/29/21	04/30/21	CHEK2	Q78*	chr22:29130478	NM_007194	-	0.88	nonsense	3062	232C>T
1034	04/29/21	04/30/21	CHEK2	L467fs*2	chr22:29090080	NM_007194	-	0.24	frameshift	2475	1400delT
1036	10/07/21	10/12/21	CHEK2	Q10*	chr22:29130682	NM_007194	-	0.24	nonsense	3756	28C>T
1036	05/10/21	05/13/21	ATR	L1182fs*34	chr3:142259782	NM_001184	-	0.14	frameshift	4923	3544delC
1036	10/07/21	10/12/21	ATR	L1182fs*34	chr3:142259782	NM_001184	-	0.23	frameshift	4685	3544delC
1056	10/27/21	11/02/21	ATM	R3008G	chr11:108236086	NM_000051	+	0.17	missense	7433	9022C>G
1072	06/03/21	06/09/21	TP53	C176G	chr17:7578404	NM_000546	-	0.45	missense	4201	526T>G

1072	06/03/21	06/09/21	TP53	"splice site 993 1G>A"	chr17:7576852	NM_000546	-	0.14	splice	4304	993 1G>A
1084	05/19/21	05/21/21	TP53	C275Y	chr17:7577114	NM_000546	-	0.18	missense	6192	824G>A
1108	09/23/21	09/25/21	TP53	W53*	chr17:7579529	NM_000546	-	0.22	nonsense	2695	158G>A
1119	08/23/21	08/25/21	TP53	I195T	chr17:7578265	NM_000546	-	2.67	missense	2211	584T>C
1119	08/23/21	08/25/21	TP53	P278A	chr17:7577106	NM_000546	-	0.25	missense	2438	832C>G
1119	08/23/21	08/25/21	FANCA	"splice site 1360-1G>A"	chr16:89851373	NM_000135	-	0.56	splice	891	1360-1G>A
1134	06/03/21	06/09/21	TP53	P190L	chr17:7578280	NM_000546	-	0.18	missense	5998	569C>T
1136	05/16/21	05/20/21	TP53	E271V	chr17:7577126	NM_000546	-	0.15	missense	6640	812A>T
1148	05/20/21	05/25/21	CHEK2	"splice site 444 1G>A"	chr22:29121230	NM_007194	-	48.93	splice	2532	444 1G>A
1149	05/18/21	05/21/21	TP53	P278L	chr17:7577105	NM_000546	-	0.15	missense	4789	833C>T
1161	06/09/21	06/16/21	TP53	E285K	chr17:7577085	NM_000546	-	0.22	missense	4027	853G>A
1161	12/08/21	12/09/21	TP53	E285K	chr17:7577085	NM_000546	-	0.43	missense	8182	853G>A
1161	01/17/22	01/20/22	TP53	E285K	chr17:7577085	NM_000546	-	0.22	missense	4525	853G>A
1161	06/09/21	06/16/21	ATM	N883fs*14	chr11:10813914 6	NM_000051	+	0.27	frameshift	3718	2649_2655delT CCTTTA
1164	06/02/22	06/07/22	CHEK2	splice site 847- 1G>C	chr22:29099555	NM_007194	-	0.18	splice	3321	847-1G>C
1164	06/02/22	06/07/22	ATR	E1992fs*5	chr3:142212075	NM_001184	-	0.2	frameshift	7045	5973_5976delT GAG
1165	07/23/21	07/30/21	TP53	L194R	chr17:7578268	NM_000546	-	0.25	missense	3605	581T>G
1173	06/10/21	06/21/21	TP53	C242W	chr17:7577555	NM_000546	-	0.21	missense	3861	726C>G
1173	09/16/21	09/20/21	TP53	C242W	chr17:7577555	NM_000546	-	0.26	missense	3102	726C>G
1179	05/11/21	05/14/21	TP53	R248Q	chr17:7577538	NM_000546	-	0.35	missense	5085	743G>A
1179	05/11/21	05/14/21	BRCA2	S1468F	chr13:32912895	NM_000059	+	0.12	missense	5975	4403C>T
1201	04/30/21	05/05/21	TP53	"splice site 993 1G>T"	chr17:7576852	NM_000546	-	2.84	splice	3169	993 1G>T
1201	10/14/21	10/15/21	TP53	splice site 993 1G>T	chr17:7576852	NM_000546	-	1.19	splice	3701	993 1G>T
1203	05/17/21	05/20/21	TP53	R280T	chr17:7577099	NM_000546	-	0.28	missense	2811	839G>C
1214	06/25/21	06/28/21	TP53	R273H	chr17:7577120	NM_000546	-	0.4	missense	2274	818G>A
1214	06/25/21	06/28/21	TP53	P278S	chr17:7577106	NM_000546	-	0.87	missense	2306	832C>T
1214	06/25/21	06/28/21	CHEK2	"splice site 444 1G>A"	chr22:29121230	NM_007194	-	1.16	splice	1717	444 1G>A

1214	06/25/21	06/28/21	ATM	R3008H	chr11:108236087	NM_000051	+	0.22	missense	2753	9023G>A
1229	04/27/21	04/28/21	TP53	A189T	chr17:7578284	NM_000546	-	0.53	missense	3757	565G>A
1240	06/08/21	06/16/21	FANCL	"splice site 472-1G>C"	chr2:58425798	NM_018062	-	49.07	splice	701	472-1G>C
1250	04/15/22	04/20/22	TP53	splice site 783-1G>T	chr17:7577156	NM_000546	-	5.93	splice	6403	783-1G>T
1261	06/22/21	06/24/21	TP53	"splice site 673-2A>G"	chr17:7577610	NM_000546	-	2.18	splice	3353	673-2A>G
1261	06/22/21	06/24/21	CHEK2	V300fs*5	chr22:29099503	NM_007194	-	0.33	frameshift	2426	897_898insTT
1263	07/27/22	07/29/22	CHEK2	I157fs*4	chr22:29121085	NM_007194	-	0.12	frameshift	4237	471delT
1272	10/05/21	10/06/21	TP53	R280G	chr17:7577100	NM_000546	-	6.85	missense	2976	838A>G
1272	06/22/22	06/23/22	TP53	R280G	chr17:7577100	NM_000546	-	2.02	missense	2375	838A>G
1287	10/04/21	10/05/21	TP53	E286G	chr17:7577081	NM_000546	-	0.11	missense	5448	857A>G
1287	10/04/21	10/05/21	ATR	"splice site 6552 1G>T"	chr3:142188178	NM_001184	-	1.16	splice	3099	6552 1G>T
1292	06/08/21	06/17/21	TP53	Q38*	chr17:7579575	NM_000546	-	0.48	nonsense	5188	112C>T
1292	06/08/21	06/17/21	TP53	Y163C	chr17:7578442	NM_000546	-	0.11	missense	6367	488A>G
1292	06/08/21	06/17/21	TP53	R181P	chr17:7578388	NM_000546	-	0.27	missense	6358	542G>C
1292	06/08/21	06/17/21	CHEK2	I157T	chr22:29121087	NM_007194	-	0.18	missense	3801	470T>C
1292	06/08/21	06/17/21	CHEK2	P182fs*2	chr22:29121011	NM_007194	-	0.17	frameshift	4063	545delC
1298	06/24/21	06/28/21	FANCC	"splice site 1154 1G>C"	chr9:97876910	NM_000136	-	0.34	splice	1748	1154 1G>C
1316	05/26/21	05/29/21	TP53	V143M	chr17:7578503	NM_000546	-	0.63	missense	4598	427G>A
1355	06/11/21	06/21/21	TP53	C176W	chr17:7578402	NM_000546	-	0.1	missense	4874	528C>G
1355	06/11/21	06/21/21	ATM	G2891D	chr11:108224493	NM_000051	+	0.28	missense	3872	8672G>A
1356	07/06/21	07/09/21	TP53	H179R	chr17:7578394	NM_000546	-	0.6	missense	5125	536A>G
1356	07/06/21	07/09/21	TP53	M246L	chr17:7577545	NM_000546	-	0.25	missense	4041	736A>T
1356	07/06/21	07/09/21	ATM	R3008H	chr11:108236087	NM_000051	+	0.2	missense	5453	9023G>A
1363	07/09/21	07/13/21	TP53	K132R	chr17:7578535	NM_000546	-	2.78	missense	5044	395A>G
1363	07/09/21	07/13/21	CHEK2	Q209*	chr22:29115441	NM_007194	-	0.47	nonsense	2542	625C>T
1376	05/19/21	05/26/21	TP53	H193R	chr17:7578271	NM_000546	-	0.91	missense	4264	578A>G
1384	07/05/21	07/08/21	ATRX	K562fs*11	chrX:76939051	NM_000489	-	5.9	frameshift	899	1686_1697ATTAAATATTTC>TATT

1404	06/14/21	06/25/21	ATRX	R418*	chrX:76939496	NM_000489	-	0.39	nonsense	1267	1252C>T
1407	07/15/21	07/19/21	PALB2	splice site 3351-187_3401del238	chr16:23614939	NM_024675	-	0.42	splice	4952	3351-187_3401del238
1410	07/16/21	07/19/21	TP53	"splice site 919 1G>C"	chr17:7577018	NM_000546	-	0.64	splice	7948	919 1G>C
1415	11/30/21	12/02/21	TP53	A159V	chr17:7578454	NM_000546	-	0.67	missense	3145	476C>T
1415	11/30/21	12/02/21	TP53	splice site 375 5G>T	chr17:7579307	NM_000546	-	0.94	splice	2243	375 5G>T
1418	06/24/21	06/28/21	TP53	M243L	chr17:7577554	NM_000546	-	0.36	missense	1938	727A>T
1421	06/24/21	06/28/21	TP53	"splice site 560-1G>A"	chr17:7578290	NM_000546	-	0.56	splice	4287	560-1G>A
1436	07/22/21	07/26/21	TP53	"splice site 375G>T"	chr17:7579312	NM_000546	-	0.29	splice	3491	375G>T
1461	09/29/21	10/04/21	ATRX	P2299fs*22	chrX:76777818	NM_000489	-	0.48	frameshift	1245	6896_6897delCT
1469	08/02/21	08/05/21	CHEK2	"splice site 1008 1G>A"	chr22:29095825	NM_007194	-	0.23	splice	3509	1008 1G>A
1476	06/25/21	06/30/21	CHEK2	L467fs*23	chr22:29090080	NM_007194	-	0.18	frameshift	3359	1400_1401insT
1477	06/21/21	06/30/21	TP53	"splice site 782 2T>G"	chr17:7577497	NM_000546	-	3.84	splice	4431	782 2T>G
1477	06/21/21	06/30/21	BRCA1	E902*	chr17:41244844	NM_007294	-	0.37	nonsense	6817	2704G>T
1479	05/31/21	06/09/21	MLH1	"splice site 1038 2T>G"	chr3:37061956	NM_000249	+	1.41	splice	1063	1038 2T>G
1480	07/07/21	07/09/21	FANCA	Q240*	chr16:89869741	NM_000135	-	0.79	nonsense	636	718C>T
1488	08/03/21	08/06/21	BRIP1	I983fs*19	chr17:59761459	NM_032043	-	0.33	frameshift	1216	2947_2948insA
1502	06/08/21	06/17/21	TP53	M237I	chr17:7577570	NM_000546	-	1	missense	3512	711G>C
1515	07/13/21	07/19/21	TP53	V218E	chr17:7578196	NM_000546	-	3.95	missense	2657	653T>A
1515	04/22/22	04/26/22	TP53	V218E	chr17:7578196	NM_000546	-	1.31	missense	4654	653T>A
1515	04/22/22	04/26/22	ATM	Y2019C	chr11:108186599	NM_000051		0.19	missense	6251	6056A>G
1541	07/28/21	07/29/21	TP53	"splice site 920-2A>G"	chr17:7576928	NM_000546	-	89.53	splice	3725	920-2A>G
1547	08/05/21	08/09/21	CHEK2	"splice site 444 1G>T"	chr22:29121230	NM_007194	-	0.32	splice	2463	444 1G>T
1549	07/29/21	08/02/21	ATM	E2039K	chr11:108186757	NM_000051	+	0.14	missense	6317	6115G>A
1552	07/02/21	07/06/21	TP53	Y163H	chr17:7578443	NM_000546	-	0.23	missense	3078	487T>C
1552	07/02/21	07/06/21	PALB2	M296fs*7	chr16:23646980	NM_024675	-	0.31	frameshift	2883	886_887insA
1552	07/02/21	07/06/21	ATR	Q1783*	chr3:142218502	NM_001184	-	0.88	nonsense	2150	5347C>T
1552	07/02/21	07/06/21	ATM	G2891D	chr11:108224493	NM_000051	+	1.19	missense	2443	8672G>A

1552	07/02/21	07/06/21	ATM	F505fs*7	chr11:108121706	NM_000051	+	0.27	frameshift	3329	1514_1515insATTACCTTTTGTGGTATAAGTTCTGAGCAAATACAAGCTGAAAACTT
1552	08/02/21	08/04/21	ATM	G2891D	chr11:108224493	NM_000051	+	1.05	missense	2482	8672G>A
1553	08/05/21	08/09/21	TP53	"splice site 375 5G>T"	chr17:7579307	NM_000546	-	7.54	splice	4376	375 5G>T
1553	08/05/21	08/09/21	TP53	G187V	chr17:7578289	NM_000546	-	1.19	missense	8512	560G>T
1553	08/05/21	08/09/21	BRCA2	E612fs*2	chr13:32907447	NM_000059	+	0.32	frameshift	6830	1833delA
1553	08/05/21	08/09/21	ATM	G2891D	chr11:108224493	NM_000051	+	0.12	missense	7781	8672G>A
1558	08/05/21	08/09/21	CHEK2	F427fs*10	chr22:29091209	NM_007194	-	0.28	frameshift	3170	1280delT
1561	08/05/21	08/10/21	TP53	R248P	chr17:7577538	NM_000546	-	0.12	missense	4276	743G>C
1587	07/20/21	07/23/21	TP53	L265fs*80	chr17:7577144	NM_000546	-	0.5	frameshift	4619	793delC
1587	07/20/21	07/23/21	TP53	M237I	chr17:7577570	NM_000546	-	0.45	missense	3301	711G>A
1587	07/20/21	07/23/21	TP53	I195T	chr17:7578265	NM_000546	-	0.43	missense	4389	584T>C
1587	07/20/21	07/23/21	TP53	R273C	chr17:7577121	NM_000546	-	0.68	missense	4000	817C>T
1587	07/20/21	07/23/21	PALB2	P1097fs*25	chr16:23619243	NM_024675	-	0.17	frameshift	4588	3290_3291delCT
1587	07/20/21	07/23/21	FANCA	E380fs*35	chr16:89858421	NM_000135	-	1.02	frameshift	881	1138delG
1587	07/20/21	07/23/21	BRIP1	"splice site 2576-2_2581delAGGACTTT"	chr17:59763520	NM_032043	-	0.65	splice	1082	2576-2_2581delAGGACTTT
1587	07/20/21	07/23/21	ATRX	L1189fs*5	chrX:76937181	NM_000489	-	0.44	frameshift	1369	3565_3566delCT
1588	08/16/21	08/19/21	ATM	I1022fs*7	chr11:108142121	NM_000051	+	0.29	frameshift	2720	3066delT
1609	08/12/21	08/16/21	FANCA	"splice site 2852 2T>A"	chr16:89828355	NM_000135	-	0.66	splice	760	2852 2T>A
1619	10/01/21	10/05/21	TP53	L194R	chr17:7578268	NM_000546	-	2.29	missense	1789	581T>G
1629	08/03/21	08/06/21	ATM	P2648fs*12	chr11:108204627	NM_000051	+	0.18	frameshift	4931	7943delC
1629	01/07/22	01/11/22	ATM	P2648fs*12	chr11:108204627	NM_000051	+	0.25	frameshift	6016	7943delC
1638	08/05/21	08/09/21	ATM	R3008C	chr11:108236086	NM_000051	+	3.54	missense	6249	9022C>T
1666	09/02/21	09/08/21	TP53	M237I	chr17:7577570	NM_000546	-	2.13	missense	3525	711G>T
1666	09/02/21	09/08/21	TP53	R249S	chr17:7577534	NM_000546	-	0.33	missense	3920	747G>T

1674	08/30/21	09/02/21	TP53	R158L	chr17:7578457	NM_000546	-	0.48	missense	7050	473G>T
1680	03/15/22	03/16/22	TP53	V173G	chr17:7578412	NM_000546	-	1.66	missense	5669	518T>G
1680	03/15/22	03/16/22	TP53	G279E	chr17:7577102	NM_000546	-	0.16	missense	5646	836G>A
1680	03/15/22	03/16/22	TP53	P177H	chr17:7578400	NM_000546	-	0.74	missense	5561	530C>A
1680	03/15/22	03/16/22	TP53	H168P	chr17:7578427	NM_000546	-	0.16	missense	5744	503A>C
1680	03/15/22	03/16/22	TP53	M246V	chr17:7577545	NM_000546	-	0.44	missense	4801	736A>G
1680	03/15/22	03/16/22	TP53	R283P	chr17:7577090	NM_000546	-	0.1	missense	5978	848G>C
1680	03/15/22	03/16/22	TP53	Q52*	chr17:7579533	NM_000546	-	0.2	nonsense	3938	154C>T
1680	03/15/22	03/16/22	FANCC	V351fs*23	chr9:97879617	NM_000136	-	0.44	frameshift	1839	1051_1052insG
1680	03/15/22	03/16/22	CHEK2	G165fs*6	chr22:29121062	NM_007194	-	0.39	frameshift	3305	494delG
1680	03/15/22	03/16/22	ATM	splice site 5763-<br>1G>C	chr11:10818088<br>6	NM_000051	+	0.23	splice	3423	5763-1G>C
1694	06/30/21	07/02/21	CHEK2	"splice site 1375<br>1G>A"	chr22:29091114	NM_007194	-	0.6	splice	1336	1375 1G>A
1695	06/08/21	06/16/21	TP53	R273C	chr17:7577121	NM_000546	-	0.77	missense	2582	817C>T
1695	06/08/21	06/16/21	CHEK2	N112I	chr22:29121340	NM_007194	-	0.31	missense	2292	335A>T
1707	08/26/21	08/30/21	TP53	D281N	chr17:7577097	NM_000546	-	0.35	missense	7179	841G>A
1730	06/18/21	06/22/21	TP53	G245D	chr17:7577547	NM_000546	-	0.18	missense	4991	734G>A
1739	07/30/21	08/02/21	BRCA2	"splice site<br>8754G>A"	chr13:32950928	NM_000059	+	0.47	splice	2790	8754G>A
1740	07/30/21	08/02/21	TP53	A161T	chr17:7578449	NM_000546	-	0.17	missense	7781	481G>A
1740	07/30/21	08/02/21	MUTYH	R83*	chr1:45799144	NM_001048171	-	0.34	nonsense	1470	247C>T
1740	07/30/21	08/02/21	ATM	R337C	chr11:10811779<br>8	NM_000051	+	0.36	missense	4406	1009C>T
1742	08/19/21	08/23/21	ATM	R2993*	chr11:10823593<br>5	NM_000051	+	0.12	nonsense	5954	8977C>T
1759	08/20/21	08/23/21	CHEK2	"splice site 444<br>1G>A"	chr22:29121230	NM_007194	-	0.59	splice	3221	444 1G>A
1786	08/02/21	08/05/21	ATM	D479fs*3	chr11:10812162<br>5	NM_000051	+	0.21	frameshift	2862	1434delA
1798	08/19/21	08/23/21	FANCA	W911fs*31	chr16:89831344	NM_000135	-	1.63	frameshift	1351	2730_2731delC<br>T
1798	08/19/21	08/23/21	ATR	F1091fs*28	chr3:142266650	NM_001184	-	0.36	frameshift	4768	3273delT
1819	09/17/21	09/20/21	ATM	V1729fs*20	chr11:10817238<br>1	NM_000051	+	0.25	frameshift	2847	5184_5185insA
1820	08/20/21	08/23/21	TP53	E286V	chr17:7577081	NM_000546	-	0.85	missense	3310	857A>T
1859	10/11/21	10/12/21	TP53	T155I	chr17:7578466	NM_000546	-	0.12	missense	6401	464C>T

1863	09/14/21	09/17/21	TP53	"splice site 783-1G>A"	chr17:7577156	NM_000546	-	0.63	splice	3474	783-1G>A
1868	09/13/21	09/16/21	TP53	S241Y	chr17:7577559	NM_000546	-	0.28	missense	3879	722C>A
1883	09/13/21	09/16/21	TP53	P190S	chr17:7578281	NM_000546	-	0.21	missense	5181	568C>T
1901	05/31/21	06/09/21	TP53	C277Y	chr17:7577108	NM_000546	-	0.26	missense	6050	830G>A
1936	09/22/21	09/27/21	ATM	L1283P	chr11:108155055	NM_000051	+	0.14	missense	6414	3848T>C
1947	09/23/21	09/27/21	TP53	"splice site 783-1G>A"	chr17:7577156	NM_000546	-	22.03	splice	3509	783-1G>A
1952	12/20/21	12/22/21	TP53	M237I	chr17:7577570	NM_000546	-	0.27	missense	2953	711G>A
1952	12/20/21	12/22/21	TP53	R280I	chr17:7577099	NM_000546	-	0.47	missense	4883	839G>T
1952	12/20/21	12/22/21	CHEK2	L467fs*2	chr22:29090080	NM_007194	-	0.92	frameshift	3590	1400delT
1960	09/30/21	10/05/21	TP53	I195T	chr17:7578265	NM_000546	-	0.15	missense	5392	584T>C
1961	09/30/21	10/04/21	ATM	R2034*	chr11:108186742	NM_000051	+	0.23	nonsense	6824	6100C>T
1985	09/28/21	10/01/21	ATM	L2452P	chr11:108200988	NM_000051	+	0.17	missense	4093	7355T>C
1991	09/30/21	10/04/21	TP53	N131I	chr17:7578538	NM_000546	-	0.57	missense	2283	392A>T
1995	09/14/21	09/17/21	CHEK2	"splice site 1096-2A>G"	chr22:29091863	NM_007194	-	5.02	splice	1974	1096-2A>G
2011	09/15/21	09/17/21	TP53	R213P	chr17:7578211	NM_000546	-	0.35	missense	4600	638G>C
2019	10/04/21	10/05/21	ATM	R2939*	chr11:108225566	NM_000051	+	0.14	nonsense	4963	8815A>T
2020	10/04/21	10/05/21	CHEK2	"splice site 1461 1G>A"	chr22:29090019	NM_007194	-	1.36	splice	2570	1461 1G>A
2045	09/07/21	09/13/21	TP53	G245S	chr17:7577548	NM_000546	-	0.16	missense	3110	733G>A
2071	08/10/21	08/13/21	ATM	D2016Y	chr11:108186589	NM_000051	+	0.78	missense	3188	6046G>T
2071	08/10/21	08/13/21	ATM	S2394L	chr11:108199839	NM_000051	+	0.95	missense	3375	7181C>T
2091	09/28/21	10/01/21	TP53	L257Q	chr17:7577511	NM_000546	-	1.61	missense	3284	770T>A
2091	09/28/21	10/01/21	TP53	L194R	chr17:7578268	NM_000546	-	0.24	missense	3367	581T>G
2094	09/29/21	10/04/21	TP53	R213*	chr17:7578212	NM_000546	-	0.25	nonsense	3201	637C>T
2094	01/12/22	01/14/22	TP53	R213*	chr17:7578212	NM_000546	-	0.26	nonsense	3406	637C>T
2094	01/12/22	01/14/22	TP53	R213G	chr17:7578212	NM_000546	-	0.2	missense	3575	637C>G
2094	09/29/21	10/04/21	ATM	R3008C	chr11:108236086	NM_000051	+	0.13	missense	4651	9022C>T
2102	10/05/21	10/07/21	TP53	V143M	chr17:7578503	NM_000546	-	0.1	missense	5740	427G>A

2111	10/07/21	10/08/21	CHEK2	E107*	chr22:29130391	NM_007194	-	0.34	nonsense	1782	319G>T
2113	07/21/22	07/22/22	ATM	K102fs*12	chr11:108100023	NM_000051		1.46	frameshift	4917	305_306delAA
2124	08/27/21	09/01/21	TP53	Y220C	chr17:7578190	NM_000546	-	1.1	missense	3455	659A>G
2138	10/01/21	10/05/21	TP53	N131S	chr17:7578538	NM_000546	-	6.2	missense	4693	392A>G
2138	10/01/21	10/05/21	TP53	R273G	chr17:7577121	NM_000546	-	8.52	missense	4437	817C>G
2138	10/01/21	10/05/21	CHEK2	L63*	chr22:29130522	NM_007194	-	0.73	nonsense	5765	188T>G
2138	10/01/21	10/05/21	ATM	G2891D	chr11:108224493	NM_000051	+	0.66	missense	3637	8672G>A
2162	10/21/21	10/26/21	TP53	Y220C	chr17:7578190	NM_000546	-	0.37	missense	3479	659A>G
2162	10/21/21	10/26/21	TP53	G325*	chr17:7576873	NM_000546	-	0.34	nonsense	5899	973G>T
2162	10/21/21	10/26/21	ATM	E1622fs*11	chr11:108165740	NM_000051		0.21	frameshift	4859	4864delG
2169	09/02/21	09/07/21	TP53	"splice site 97-5_101delTACAGTCCCC"	chr17:7579585	NM_000546	-	28.94	splice	3818	97-5_101delTACAGTCCCC
2181	08/25/21	08/30/21	ATM	"splice site 6453-1_6457delGAGTAA"	chr11:108192026	NM_000051	+	50.2	splice	2958	6453-1_6457delGAGTAA
2241	11/23/21	11/24/21	ATM	R2486*	chr11:108201089	NM_000051		0.21	nonsense	5330	7456C>T
2241	11/23/21	11/24/21	ATM	G2891D	chr11:108224493	NM_000051		0.23	missense	5965	8672G>A
2348	02/03/22	02/04/22	TP53	N239D	chr17:7577566	NM_000546	-	0.14	missense	4147	715A>G
2348	02/03/22	02/04/22	MLH1	K453fs*38	chr3:37067446	NM_000249		1.34	frameshift	1342	1358delA
2348	02/03/22	02/04/22	ATM	S1993fs*23	chr11:108183197	NM_000051		0.26	frameshift	3469	5979_5983delTAAAG
2352	10/26/21	10/28/21	TP53	R196P	chr17:7578262	NM_000546	-	1.38	missense	2672	587G>C
2367	11/22/21	11/24/21	ATM	R3008C	chr11:108236086	NM_000051	+	0.59	missense	5053	9022C>T
2373	11/18/21	11/22/21	NBN	N71fs*21	chr8:90993708	NM_002485	-	2.16	frameshift	510	212_215ATTC>GAA
2374	11/10/21	11/16/21	TP53	C135fs*35	chr17:7578526	NM_000546	-	0.16	frameshift	3171	403delT
2398	11/02/21	11/05/21	ATM	L2427R	chr11:108199938	NM_000051	+	0.2	missense	5076	7280T>G
2419	11/15/21	11/17/21	ATM	S2408L	chr11:108199881	NM_000051		0.38	missense	3901	7223C>T
2424	11/17/21	11/18/21	MUTYH	Y165C	chr1:45798475	NM_001048171	-	49.74	missense	1908	494A>G
2428	11/12/21	11/15/21	TP53	splice site 375 5G>T	chr17:7579307	NM_000546	-	26.14	splice	3714	375 5G>T
2428	11/12/21	11/15/21	CHEK2	R523fs*43	chr22:29083953	NM_007194	-	0.24	frameshift	4252	1563delG

2451	11/19/21	11/22/21	RAD51	T88>H*	chr15:40998410	NM_002875	+	1.3	nonsense	923	261_262insCATTAGTTCCAATGGAACCAATTAGTTCCAATGGGTTTCACC
2458	12/13/21	12/15/21	TP53	R283P	chr17:7577090	NM_000546	-	0.16	missense	8606	848G>C
2458	12/13/21	12/15/21	TP53	V272G	chr17:7577123	NM_000546	-	0.29	missense	7597	815T>G
2458	12/13/21	12/15/21	TP53	V216M	chr17:7578203	NM_000546	-	0.13	missense	4596	646G>A
2462	11/25/21	11/30/21	TP53	C135Y	chr17:7578526	NM_000546	-	0.53	missense	2823	404G>A
2462	11/25/21	11/30/21	TP53	K132E	chr17:7578536	NM_000546	-	0.68	missense	2954	394A>G
2462	11/25/21	11/30/21	CHEK2	splice site 909-1G>A	chr22:29095926	NM_007194	-	0.21	splice	2384	909-1G>A
2477	12/07/21	12/08/21	TP53	M237I	chr17:7577570	NM_000546	-	1.23	missense	4140	711G>C
2478	12/02/21	12/06/21	ATM	R3047*	chr11:108236203	NM_000051		1.31	nonsense	4360	9139C>T
2478	12/02/21	12/06/21	ATM	L345fs*1	chr11:108117822	NM_000051		0.16	frameshift	4325	1034delT
2499	11/25/21	11/26/21	TP53	Y234C	chr17:7577580	NM_000546	-	6.5	missense	3951	701A>G
2499	11/25/21	11/26/21	RAD51B	splice site 315+1G>A	chr14:68301914	NM_133509	+	57.02	splice	1261	315+1G>A
2575	12/01/21	12/06/21	TP53	G244D	chr17:7577550	NM_000546	-	0.16	missense	3132	731G>A
2590	01/17/22	01/24/22	TP53	splice site 991_9935delCAGGTACT	chr17:7576847	NM_000546	-	2.26	splice	6446	991_9935delCAGGTACT
2607	12/15/21	12/20/21	TP53	N239D	chr17:7577566	NM_000546	-	0.85	missense	3187	715A>G
2635	11/16/21	11/18/21	MSH3	L711fs*1	chr5:80064700	NM_002439		0.62	frameshift	650	2132_2136delTAATA
2635	11/16/21	11/18/21	ATM	G2891D	chr11:108224493	NM_000051		0.38	missense	1825	8672G>A
2651	12/24/21	12/28/21	BRCA2	Q154*	chr13:32900272	NM_000059	+	3.26	nonsense	3951	460C>T
2667	12/10/21	12/14/21	CHEK2	splice site 684-1G>A	chr22:29108006	NM_007194	-	0.89	splice	2912	684-1G>A
2667	12/10/21	12/14/21	ATM	R337C	chr11:108117798	NM_000051	+	0.74	missense	3391	1009C>T
2667	12/10/21	12/14/21	ATM	G2891D	chr11:108224493	NM_000051	+	0.16	missense	4379	8672G>A
2670	12/24/21	12/28/21	RAD54L	C391fs*1	chr1:46736380	NM_003579		26.31	frameshift	1205	1092_1093insCGAGACGCTGCTGCTAGTGAGGCAGACAGGCAGCTAGGAGAGGAGCGGCTGCGGGAGCTCACCAG

											CATTGTGAATAGGTAATGACCTTAAGC
2672	02/01/22	02/04/22	TP53	C141Y	chr17:7578508	NM_000546	-	1.37	missense	7747	422G>A
2689	01/05/22	01/07/22	ATM	splice site 72+2_72+5delTAGT	chr11:108098424	NM_000051	+	0.39	splice	3854	72+2_72+5delTAGT
2689	01/05/22	01/07/22	ATM	splice site 8786+1G>A	chr11:108224608	NM_000051	+	4.22	splice	4450	8786+1G>A
2689	01/05/22	01/07/22	ATM	R2763fs*43	chr11:108213967	NM_000051	+	0.62	frameshift	4548	8288delG
2689	01/05/22	01/07/22	ATM	R23*	chr11:108098418	NM_000051	+	0.54	nonsense	3500	67C>T
2689	01/05/22	01/07/22	ATM	splice site 2921+1G>A	chr11:108141874	NM_000051	+	1.23	splice	3415	2921+1G>A
2689	01/05/22	01/07/22	ATM	K2749*	chr11:108206665	NM_000051	+	0.17	nonsense	4211	8245A>T
2689	01/05/22	01/07/22	ATM	I2629fs*25	chr11:108203585	NM_000051	+	0.61	frameshift	3767	7886_7890delTATTA
2691	01/25/22	01/27/22	TP53	G245S	chr17:7577548	NM_000546	-	24.51	missense	6075	733G>A
2691	01/25/22	01/27/22	PMS2	K301N	chr7:6035165	NM_000535	-	47.03	missense	572	903G>T
2730	11/09/21	11/11/21	ATM	splice site 2921 1G>A	chr11:108141874	NM_000051		47.97	splice	3982	2921 1G>A
2773	01/05/22	01/07/22	TP53	R175H	chr17:7578406	NM_000546	-	4.29	missense	3637	524G>A
2773	01/05/22	01/07/22	TP53	L252P	chr17:7577526	NM_000546	-	0.86	missense	3585	755T>C
2773	01/05/22	01/07/22	ATM	R2486G	chr11:108201089	NM_000051		0.39	missense	3312	7456C>G
2773	01/05/22	01/07/22	ATM	D2507fs*10	chr11:108202175	NM_000051		1.46	frameshift	2952	7520_7521insGAGA
2773	01/05/22	01/07/22	ATM	W2091*	chr11:108188174	NM_000051		0.16	nonsense	3654	6273G>A
2800	12/28/21	12/29/21	CHEK2	splice site 684-2A>G	chr22:29108007	NM_007194	-	0.3	splice	3027	684-2A>G
2800	12/28/21	12/29/21	CHEK2	E8fs*53	chr22:29130688	NM_007194	-	0.33	frameshift	3055	21delT
2802	12/31/21	01/07/22	TP53	L194R	chr17:7578268	NM_000546	-	0.15	missense	4592	581T>G
2902	01/13/22	01/19/22	TP53	E286G	chr17:7577081	NM_000546	-	86.74	missense	8664	857A>G

**Concordant mutations**											
**ID**	**Collection date ? (#1)**	**Received date ? (#1)**	**Gene ? (#1)**	**Alteration (#1)**	**Chromosome: position**	**Transcript ?**	**Strand ?**	**Percent read ?**	**Functional effect ?**	**Depth ?**	**CDS effect ?**
14	02/25/21	03/01/21	TP53	R248W	chr17:7577539	NM_000546	-	17.93	missense	5958	742C>T
17	03/04/21	03/08/21	TP53	R175H	chr17:7578406	NM_000546	-	3.1	missense	4618	524G>A

22	04/02/21	04/08/21	TP53	V73fs*51	chr17:7579470	NM_000546	-	20.06	frameshift	4751	216_217insCC
23	03/16/21	03/19/21	MUTYH	G382D	chr1:45797228	NM_001048171	-	49.42	missense	1125	1145G>A
28	04/07/21	04/09/21	ATRX	R418*	chrX:76939496	NM_000489	-	2.06	nonsense	1360	1252C>T
29	03/18/21	03/22/21	TP53	K132Q	chr17:7578536	NM_000546	-	2.31	missense	5937	394A>C
30	03/31/21	04/05/21	TP53	V73fs*76	chr17:7579471	NM_000546	-	0.97	frameshift	4451	215_216insG
32	03/18/22	03/22/22	TP53	D208I	chr17:7578226	NM_000546	-	1.52	missense	5082	622_623GA>AT
50	02/09/21	02/12/21	TP53	R213*	chr17:7578212	NM_000546	-	0.26	nonsense	2661	637C>T
57	04/01/21	04/05/21	MSH6	F1088fs*5	chr2:48030647	NM_000179	+	45.96	frameshift	570	3261_3262insC
60	04/01/21	04/05/21	TP53	K132Q	chr17:7578536	NM_000546	-	3.25	missense	4649	394A>C
79	04/15/21	04/19/21	TP53	K132R	chr17:7578535	NM_000546	-	0.3	missense	2361	395A>G
89	04/08/21	04/12/21	TP53	R175H	chr17:7578406	NM_000546	-	0.46	missense	5382	524G>A
89	04/08/21	04/12/21	TP53	R175H	chr17:7578406	NM_000546	-	0.46	missense	5382	524G>A
93	04/09/21	04/12/21	MUTYH	G382D	chr1:45797228	NM_001048171	-	48.92	missense	1386	1145G>A
96	04/09/21	04/13/21	TP53	R213L	chr17:7578211	NM_000546	-	11.92	missense	5703	638G>T
120	04/14/21	04/16/21	TP53	Q317*	chr17:7576897	NM_000546	-	2.14	nonsense	6312	949C>T
141	09/03/21	09/08/21	TP53	G245A	chr17:7577547	NM_000546	-	21.67	missense	2870	734G>C
142	04/20/21	04/23/21	TP53	V157F	chr17:7578461	NM_000546	-	55.38	missense	4749	469G>T
142	06/24/21	06/28/21	TP53	V157F	chr17:7578461	NM_000546	-	42.34	missense	5291	469G>T
144	03/05/21	03/09/21	TP53	F212fs*3	chr17:7578212	NM_000546	-	0.4	frameshift	4030	635_636delTT
144	03/05/21	03/09/21	TP53	G245D	chr17:7577547	NM_000546	-	0.31	missense	4223	734G>A
147	01/15/21	01/18/21	TP53	M237I	chr17:7577570	NM_000546	-	9.09	missense	3157	711G>A
147	01/15/21	01/18/21	MUTYH	G382D	chr1:45797228	NM_001048171	-	44.57	missense	1050	1145G>A
151	03/12/21	03/17/21	TP53	R273H	chr17:7577120	NM_000546	-	10.43	missense	4804	818G>A
160	04/15/21	04/19/21	TP53	W53*	chr17:7579529	NM_000546	-	0.66	nonsense	3008	158G>A
161	04/16/21	04/19/21	TP53	P190fs*57	chr17:7578279	NM_000546	-	16.17	frameshift	4825	569delC
178	07/19/21	07/21/21	ATM	L1238fs*6	chr11:108153571	NM_000051	+	55.9	frameshift	4467	3712_3716delTTATT
178	04/29/21	05/03/21	ATM	L1238fs*6	chr11:108153571	NM_000051	+	49.54	frameshift	4392	3712_3716delTTATT
183	02/09/21	02/12/21	BRCA2	I605fs*2	chr13:32907428	NM_000059	+	0.64	frameshift	4227	1813_1817ATACC>TACT

183	02/09/21	02/12/21	ATR	S2317fs*8	chr3:142184030	NM_001184	-	0.87	frameshift	2983	6949delT
184	02/01/21	02/03/21	MLH1	I565fs*3	chr3:37083784	NM_000249	+	1.75	frameshift	513	1693_1694insA
189	02/05/21	02/08/21	TP53	K132N	chr17:7578534	NM_000546	-	1.37	missense	4729	396G>T
190	01/26/21	01/29/21	MSH3	K383fs*32	chr5:79970921	NM_002439	+	12.08	frameshift	480	1148delA
190	01/26/21	01/29/21	MLH1	S269*	chr3:37059012	NM_000249	+	12.38	nonsense	824	806C>G
190	01/26/21	01/29/21	BARD1	K208fs*4	chr2:215645974	NM_000465	-	10.26	frameshift	1364	623delA
190	01/26/21	01/29/21	ATM	R3047*	chr11:108236203	NM_000051	+	9.61	nonsense	5848	9139C>T
191	02/10/21	02/13/21	TP53	M1K	chr17:7578532	NM_001126117	-	1.25	nonsense	4156	2T>A
193	01/25/21	01/28/21	TP53	H193Y	chr17:7578272	NM_000546	-	10.34	missense	3036	577C>T
193	01/25/21	01/28/21	MUTYH	G382D	chr1:45797228	NM_001048171	-	46.21	missense	1028	1145G>A
200	03/12/21	03/15/21	TP53	G262V	chr17:7577153	NM_000546	-	0.21	missense	5609	785G>T
200	11/22/21	11/24/21	TP53	G262V	chr17:7577153	NM_000546	-	15.62	missense	5562	785G>T
201	02/25/21	03/01/21	TP53	R248Q	chr17:7577538	NM_000546	-	2.88	missense	2293	743G>A
202	01/21/21	01/25/21	TP53	I232F	chr17:7577587	NM_000546	-	6.13	missense	5514	693_694CA>AT
203	01/27/21	02/01/21	TP53	R209fs*6	chr17:7578221	NM_000546	-	1.72	frameshift	3482	626_627delGA
206	01/20/21	01/22/21	NBN	A32fs*4	chr8:90995026	NM_002485	-	47.73	frameshift	528	93_94delTG
213	01/26/21	01/28/21	TP53	R282W	chr17:7577094	NM_000546	-	2.93	missense	3139	844C>T
215	03/11/21	03/15/21	TP53	E62*	chr17:7579503	NM_000546	-	2.65	nonsense	7064	184G>T
216	12/22/20	12/24/20	TP53	C238S	chr17:7577569	NM_000546	-	49.84	missense	3192	712T>A
219	01/26/21	01/28/21	TP53	R175H	chr17:7578406	NM_000546	-	20.03	missense	5502	524G>A
220	02/23/21	02/25/21	BRCA2	V1486fs*5	chr13:32912947	NM_000059	+	52.52	frameshift	5027	4456_4459delGTTA
221	02/24/21	02/25/21	TP53	R175H	chr17:7578406	NM_000546	-	57.43	missense	5405	524G>A
223	03/30/21	04/01/21	MUTYH	G382D	chr1:45797228	NM_001048171	-	51.06	missense	942	1145G>A
227	02/10/21	02/13/21	TP53	Y234C	chr17:7577580	NM_000546	-	1.95	missense	6363	701A>G
228	03/29/22	03/31/22	TP53	splice site 994-1G>C	chr17:7574034	NM_000546	-	3.97	splice	4864	994-1G>C
228	02/01/22	02/04/22	TP53	splice site 994-1G>C	chr17:7574034	NM_000546	-	1.39	splice	4887	994-1G>C
230	02/16/21	03/01/21	TP53	L137Q	chr17:7578520	NM_000546	-	9.14	missense	4080	410T>A
233	12/10/21	12/14/21	TP53	R175H	chr17:7578406	NM_000546	-	0.58	missense	6042	524G>A

233	02/05/21	02/08/21	BRCA1	E1161fs*3	chr17:41244056	NM_007294	-	50.2	frameshift	2743	3481_3491delGAAGATACTAG
233	12/10/21	12/14/21	BRCA1	E1161fs*3	chr17:41244056	NM_007294	-	48.89	frameshift	11669	3481_3491delGAAGATACTAG
237	06/10/21	06/21/21	TP53	R280T	chr17:7577099	NM_000546	-	0.2	missense	2010	839G>C
237	06/10/21	06/21/21	TP53	E346*	chr17:7573991	NM_000546	-	0.43	nonsense	1868	1036G>T
237	02/16/21	02/22/21	TP53	R280T	chr17:7577099	NM_000546	-	0.15	missense	7131	839G>C
237	02/16/21	02/22/21	TP53	E346*	chr17:7573991	NM_000546	-	0.12	nonsense	6048	1036G>T
239	02/08/21	02/10/21	TP53	Y220C	chr17:7578190	NM_000546	-	5.54	missense	1805	659A>G
239	08/12/21	08/16/21	TP53	Y220C	chr17:7578190	NM_000546	-	13.58	missense	1863	659A>G
239	02/08/21	02/10/21	CHEK1	K267fs*37	chr11:125507425	NM_001274	+	1.11	frameshift	628	801delA
239	08/12/21	08/16/21	CHEK1	K267fs*37	chr11:125507425	NM_001274	+	4.63	frameshift	842	801delA
245	03/08/21	03/12/21	TP53	R248Q	chr17:7577538	NM_000546	-	0.91	missense	6130	743G>A
246	01/28/21	01/30/21	TP53	R248W	chr17:7577539	NM_000546	-	5.18	missense	5754	742C>T
247	03/31/21	04/02/21	TP53	R213*	chr17:7578212	NM_000546	-	13.31	nonsense	5904	637C>T
247	03/31/21	04/02/21	BRCA2	V220fs*4	chr13:32903605	NM_000059	+	52.58	frameshift	5980	658_659delGT
249	01/28/21	02/01/21	TP53	H168L	chr17:7578427	NM_000546	-	29.71	missense	6470	503A>T
249	01/28/21	02/01/21	MUTYH	G382D	chr1:45797228	NM_001048171	-	64.54	missense	1190	1145G>A
254	06/10/22	06/14/22	TP53	splice site 97-2A>T	chr17:7579592	NM_000546	-	1.03	splice	3493	97-2A>T
257	01/20/21	01/22/21	TP53	W146*	chr17:7578492	NM_000546	-	0.22	nonsense	5096	438G>A
265	01/11/21	01/12/21	TP53	C176S	chr17:7578404	NM_000546	-	26.54	missense	6492	526T>A
265	09/24/21	09/29/21	TP53	C176S	chr17:7578404	NM_000546	-	79.21	missense	6018	526T>A
266	01/20/21	01/22/21	TP53	H178fs*69	chr17:7578397	NM_000546	-	26.99	frameshift	4584	532_533CA>T
269	09/14/21	09/16/21	TP53	E258fs*2	chr17:7577509	NM_000546	-	39.6	frameshift	5098	772G>CACTGA
269	12/09/21	12/13/21	TP53	E258fs*2	chr17:7577509	NM_000546	-	84.98	frameshift	4240	772G>CACTGA
272	01/14/21	01/18/21	TP53	S127fs*44	chr17:7578551	NM_000546	-	2.49	frameshift	5413	378_379insAC
273	02/01/21	02/04/21	TP53	H178fs*3	chr17:7578397	NM_000546	-	80.68	frameshift	6801	532_533insC
284	03/02/21	03/05/21	TP53	S241T	chr17:7577560	NM_000546	-	0.34	missense	6156	721T>A
284	02/17/22	02/21/22	TP53	S241T	chr17:7577560	NM_000546	-	16.41	missense	4462	721T>A
286	01/20/21	01/25/21	TP53	V157F	chr17:7578461	NM_000546	-	0.61	missense	2958	469G>T

286	01/20/21	01/25/21	TP53	G199E	chr17:7578253	NM_000546	-	0.22	missense	2261	596G>A
286	01/24/22	01/26/22	TP53	G199E	chr17:7578253	NM_000546	-	8.91	missense	5555	596G>A
286	01/24/22	01/26/22	TP53	V157F	chr17:7578461	NM_000546	-	25.4	missense	8020	469G>T
286	01/20/21	01/25/21	MUTYH	G382D	chr1:45797228	NM_001048171	-	49.57	missense	1039	1145G>A
286	01/24/22	01/26/22	MUTYH	G382D	chr1:45797228	NM_001048171	-	47.81	missense	1577	1145G>A
287	01/13/21	01/16/21	TP53	G245C	chr17:7577548	NM_000546	-	22.59	missense	4396	733G>T
288	01/12/21	01/14/21	TP53	R213*	chr17:7578212	NM_000546	-	45.18	nonsense	4781	637C>T
289	01/26/21	01/28/21	TP53	Y234C	chr17:7577580	NM_000546	-	10.84	missense	2971	701A>G
292	01/28/21	02/01/21	TP53	G245S	chr17:7577548	NM_000546	-	0.15	missense	5375	733G>A
293	02/19/21	02/23/21	TP53	E286*	chr17:7577082	NM_000546	-	3.45	nonsense	6405	856G>T
294	03/10/21	03/12/21	TP53	V272L	chr17:7577124	NM_000546	-	0.2	missense	2968	814G>T
295	02/11/21	02/15/21	TP53	H179R	chr17:7578394	NM_000546	-	14.37	missense	3556	536A>G
296	02/05/21	02/09/21	TP53	L137Q	chr17:7578520	NM_000546	-	22.9	missense	5795	410T>A
299	01/15/21	01/18/21	TP53	R175H	chr17:7578406	NM_000546	-	87.62	missense	5453	524G>A
302	01/21/21	01/25/21	TP53	R273H	chr17:7577120	NM_000546	-	72.66	missense	4279	818G>A
303	02/05/21	02/10/21	TP53	E349*	chr17:7573982	NM_000546	-	38.16	nonsense	5624	1045G>T
303	06/22/22	06/23/22	TP53	E349*	chr17:7573982	NM_000546	-	82.1	nonsense	5183	1045G>T
304	02/09/21	02/12/21	BARD1	Q615*	chr2:215609851	NM_000465	-	5.29	nonsense	813	1843C>T
308	03/30/21	04/01/21	TP53	R175H	chr17:7578406	NM_000546	-	31.21	missense	1974	524G>A
309	02/25/21	03/01/21	TP53	R175H	chr17:7578406	NM_000546	-	20.09	missense	5699	524G>A
312	02/12/21	02/25/21	TP53	R248Q	chr17:7577538	NM_000546	-	13.79	missense	3277	743G>A
314	02/02/21	02/05/21	TP53	H168P	chr17:7578427	NM_000546	-	0.9	missense	8375	503A>C
317	01/22/21	01/25/21	TP53	C277F	chr17:7577108	NM_000546	-	30.1	missense	7046	830G>T
319	02/17/21	02/20/21	TP53	G245C	chr17:7577548	NM_000546	-	1.87	missense	4750	733G>T
319	02/17/21	02/20/21	TP53	G154A	chr17:7578469	NM_000546	-	0.79	missense	5475	461G>C
327	01/12/21	01/14/21	TP53	I195T	chr17:7578265	NM_000546	-	43.05	missense	4843	584T>C
334	01/12/21	01/14/21	TP53	R213*	chr17:7578212	NM_000546	-	16.53	nonsense	3733	637C>T
346	03/03/21	03/05/21	TP53	R248W	chr17:7577539	NM_000546	-	37.77	missense	5047	742C>T

346	03/03/21	03/05/21	TP53	N29fs*14	chr17:7579709	NM_000546	-	37.57	frameshift	5733	86_87insA
347	02/09/21	02/12/21	TP53	I195T	chr17:7578265	NM_000546	-	2.2	missense	3404	584T>C
348	03/24/21	03/25/21	MUTYH	R231H	chr1:45798117	NM_001048171	-	49.07	missense	1618	692G>A
353	01/25/21	01/28/21	TP53	R282W	chr17:7577094	NM_000546	-	0.52	missense	7153	844C>T
354	02/02/21	02/05/21	NBN	K219fs*16	chr8:90983441	NM_002485	-	48.24	frameshift	483	657_661delACAA A
354	12/23/21	12/29/21	NBN	K219fs*16	chr8:90983441	NM_002485	-	51.85	frameshift	567	657_661delACAA A
358	03/09/21	03/11/21	BRCA1	C61G	chr17:41258504	NM_007294	-	49.86	missense	2782	181T>G
359	03/09/21	03/12/21	TP53	C176R	chr17:7578404	NM_000546	-	28.48	missense	4498	526T>C
360	03/04/21	03/06/21	BRIP1	R798Q	chr17:59793411	NM_032043	-	48.44	missense	609	2393G>A
363	03/16/21	03/18/21	TP53	R175H	chr17:7578406	NM_000546	-	40.99	missense	8160	524G>A
364	02/04/21	02/08/21	TP53	E271K	chr17:7577127	NM_000546	-	44.1	missense	5538	811G>A
366	01/25/21	01/28/21	TP53	G266V	chr17:7577141	NM_000546	-	9.51	missense	4489	797G>T
367	01/21/21	01/25/21	TP53	C275Y	chr17:7577114	NM_000546	-	1.11	missense	3165	824G>A
369	02/09/21	02/12/21	TP53	R248Q	chr17:7577538	NM_000546	-	4.32	missense	4907	743G>A
378	01/08/21	01/13/21	TP53	E285K	chr17:7577085	NM_000546	-	52.35	missense	6136	853G>A
388	01/29/21	02/03/21	CHEK2	T367fs*15	chr22:29091856	NM_007194	-	47.02	frameshift	2386	1100delC
388	01/29/21	02/03/21	CHEK2	V198fs*7	chr22:29120965	NM_007194	-	47.76	frameshift	2919	591delA
388	01/29/21	02/03/21	ATM	P292L	chr11:10811572 7	NM_000051	+	50.35	missense	4199	875C>T
412	06/09/21	06/16/21	TP53	E294fs*51	chr17:7577057	NM_000546	-	33.99	frameshift	8470	880delG
412	06/09/21	06/16/21	TP53	P152L	chr17:7578475	NM_000546	-	30.49	missense	6503	455C>T
412	01/14/21	01/16/21	TP53	P152L	chr17:7578475	NM_000546	-	19.44	missense	8221	455C>T
412	01/14/21	01/16/21	TP53	E294fs*51	chr17:7577057	NM_000546	-	22.26	frameshift	10361	880delG
414	01/14/21	01/16/21	TP53	S241Y	chr17:7577559	NM_000546	-	2.25	missense	3376	722C>A
420	01/19/21	01/22/21	TP53	V218G	chr17:7578196	NM_000546	-	88.7	missense	6134	653T>G
436	01/21/21	01/25/21	BRIP1	E458*	chr17:59871059	NM_032043	-	46.3	nonsense	920	1372G>T
444	01/25/21	01/28/21	RAD54L	R609*	chr1:46740345	NM_003579	+	3.53	nonsense	1161	1825C>T
444	01/25/21	01/28/21	MSH6	N1307fs*12	chr2:48033709	NM_000179	+	59.9	frameshift	2050	3920_3921insA
444	01/25/21	01/28/21	BRIP1	G615fs*23	chr17:59857711	NM_032043	-	22.23	frameshift	2830	1844_1845delGT

449	01/26/21	01/28/21	TP53	P278R	chr17:7577105	NM_000546	-	27.31	missense	7819	833C>G
462	01/28/21	02/01/21	TP53	R175H	chr17:7578406	NM_000546	-	2.54	missense	6586	524G>A
484	02/01/21	02/04/21	MSH6	F1088fs*2	chr2:48030646	NM_000179	+	9.4	frameshift	1021	3261delC
484	08/06/21	08/12/21	MSH6	F1088fs*2	chr2:48030646	NM_000179	+	8.22	frameshift	1205	3261delC
484	02/01/21	02/04/21	MLH1	R226Q	chr3:37053590	NM_000249	+	7.42	missense	836	677G>A
484	02/01/21	02/04/21	MLH1	T117M	chr3:37045935	NM_000249	+	50.91	missense	1047	350C>T
484	08/06/21	08/12/21	MLH1	T117M	chr3:37045935	NM_000249	+	49.78	missense	1147	350C>T
484	08/06/21	08/12/21	MLH1	R226Q	chr3:37053590	NM_000249	+	7.74	missense	1008	677G>A
494	02/03/21	02/05/21	TP53	E298*	chr17:7577046	NM_000546	-	1.9	nonsense	9041	892G>T
494	02/03/21	02/05/21	TP53	E298*	chr17:7577046	NM_000546	-	1.9	nonsense	9041	892G>T
508	02/04/21	02/08/21	ATM	W579*	chr11:108122693	NM_000051	+	50.14	nonsense	2579	1737G>A
526	12/03/21	12/08/21	TP53	C124fs*1	chr17:7579314	NM_000546	-	37.13	frameshift	3973	372delC
526	02/05/21	02/10/21	BRCA1	R1203*	chr17:41243941	NM_007294	-	48.88	nonsense	3742	3607C>T
526	12/03/21	12/08/21	BRCA1	R1203*	chr17:41243941	NM_007294	-	44.46	nonsense	6358	3607C>T
536	02/09/21	02/12/21	BRCA2	S1882*	chr13:32914137	NM_000059	+	49.03	nonsense	3979	5645C>A
536	12/21/21	12/27/21	BRCA2	S1882*	chr13:32914137	NM_000059	-	51.26	nonsense	6213	5645C>A
566	02/15/21	02/18/21	TP53	R273C	chr17:7577121	NM_000546	-	16.97	missense	5187	817C>T
566	02/15/21	02/18/21	ATM	S646*	chr11:108124579	NM_000051	+	18.08	nonsense	7509	1937C>A
577	02/17/21	02/22/21	TP53	V216M	chr17:7578203	NM_000546	-	0.78	missense	4882	646G>A
577	02/17/21	02/22/21	TP53	E285K	chr17:7577085	NM_000546	-	3.86	missense	8138	853G>A
580	02/17/21	02/20/21	TP53	R273H	chr17:7577120	NM_000546	-	11.38	missense	4920	818G>A
583	02/18/21	02/23/21	TP53	A276D	chr17:7577111	NM_000546	-	2.06	missense	2955	827C>A
601	02/22/21	02/24/21	TP53	R158fs*21	chr17:7578452	NM_000546	-	2.33	frameshift	5321	473_477delGCGCC
609	02/23/21	02/25/21	TP53	P278A	chr17:7577106	NM_000546	-	0.25	missense	7133	832C>G
610	02/23/21	02/26/21	MUTYH	G382D	chr1:45797228	NM_001048171	-	52.18	missense	941	1145G>A
629	02/25/21	03/01/21	TP53	P250L	chr17:7577531	NM_000546	-	0.73	missense	4934	749_750CC>TT
629	02/25/21	03/01/21	ATM	W1805*	chr11:108173674	NM_000051	+	1.13	nonsense	4497	5414G>A
629	02/25/21	03/01/21	ATM	Q675*	chr11:108124665	NM_000051	+	1.11	nonsense	5231	2023C>T

647	03/02/21	03/05/21	TP53	N235_Y236del	chr17:7577571	NM_000546	-	3.02	nonframeshift	1820	704_709delACTACA
649	03/02/21	03/05/21	TP53	R280S	chr17:7577098	NM_000546	-	47.77	missense	986	840A>C
658	03/03/21	03/08/21	TP53	C141*	chr17:7578507	NM_000546	-	33.68	nonsense	2586	423C>A
687	03/08/21	03/11/21	ATM	D2720N	chr11:108206578	NM_000051	+	33.05	missense	6935	8158G>A
688	03/08/21	03/11/21	TP53	R337L	chr17:7574017	NM_000546	-	2.61	missense	3336	1010G>T
689	03/09/21	03/11/21	TP53	S99fs*23	chr17:7579389	NM_000546	-	36.23	frameshift	6996	294_297delTTCC
715	03/12/21	03/15/21	ATR	E833fs*10	chr3:142272701	NM_001184	-	51.72	frameshift	3890	2497delG
718	03/12/21	03/17/21	MUTYH	M269V	chr1:45797924	NM_001048171	-	50.03	missense	1663	805A>G
749	03/17/21	03/22/21	TP53	S215G	chr17:7578206	NM_000546	-	15.18	missense	3868	643A>G
749	12/23/21	12/29/21	TP53	S215G	chr17:7578206	NM_000546	-	5.22	missense	3426	643A>G
749	03/17/22	03/21/22	TP53	S215G	chr17:7578206	NM_000546	-	0.16	missense	5148	643A>G
749	07/01/22	07/07/22	TP53	S215G	chr17:7578206	NM_000546	-	3.52	missense	5427	643A>G
758	03/18/21	03/22/21	TP53	R248Q	chr17:7577538	NM_000546	-	47.32	missense	4592	743G>A
759	03/18/21	03/23/21	TP53	E286K	chr17:7577082	NM_000546	-	1.47	missense	6674	856G>A
767	03/19/21	03/24/21	TP53	R273C	chr17:7577121	NM_000546	-	35.14	missense	4587	817C>T
768	03/19/21	03/23/21	TP53	W146*	chr17:7578493	NM_000546	-	0.19	nonsense	3742	437G>A
819	03/26/21	03/29/21	TP53	R342*	chr17:7574003	NM_000546	-	15.51	nonsense	5482	1024C>T
819	03/26/21	03/29/21	TP53	E68*	chr17:7579485	NM_000546	-	32.91	nonsense	8948	202G>T
861	04/20/21	04/22/21	TP53	E286K	chr17:7577082	NM_000546	-	38.16	missense	4256	856G>A
868	02/22/22	02/24/22	BRCA2	S455*	chr13:32906979	NM_000059		1.56	nonsense	7353	1364_1365CA>AG
868	02/22/22	02/24/22	BRCA2	R2659G	chr13:32936829	NM_000059		46.55	missense	5104	7975A>G
876	05/14/21	05/19/21	TP53	R175H	chr17:7578406	NM_000546	-	0.43	missense	2550	524G>A
880	05/05/21	05/08/21	TP53	Y103*	chr17:7579378	NM_000546	-	48.65	nonsense	4187	309C>G
884	09/16/21	09/20/21	ATRX	K700fs*10	chrX:76938650	NM_000489	-	1.46	frameshift	755	2097_2098insTT
901	04/20/21	04/23/21	TP53	V274F	chr17:7577118	NM_000546	-	7.73	missense	4747	820G>T
917	05/05/21	05/08/21	TP53	V173L	chr17:7578413	NM_000546	-	74.98	missense	5588	517G>T
922	05/06/21	05/10/21	TP53	Y234C	chr17:7577580	NM_000546	-	0.55	missense	6307	701A>G
922	05/06/21	05/10/21	ATM	I1581fs*5	chr11:108164169	NM_000051	+	0.33	frameshift	5512	4741_4742insA

927	05/06/21	05/10/21	TP53	Y163*	chr17:7578441	NM_000546	-	2.72	nonsense	5265	489C>A
929	04/20/21	04/23/21	TP53	R249M	chr17:7577535	NM_000546	-	55.19	missense	5568	746G>T
938	04/28/21	04/29/21	TP53	R175H	chr17:7578406	NM_000546	-	5.59	missense	5492	524G>A
938	04/28/21	04/29/21	MUTYH	Y165C	chr1:45798475	NM_001048171	-	51.4	missense	2537	494A>G
940	04/22/21	04/26/21	TP53	R282W	chr17:7577094	NM_000546	-	16.16	missense	5831	844C>T
962	04/16/21	04/20/21	TP53	C242Y	chr17:7577556	NM_000546	-	34.5	missense	3768	725G>A
963	04/27/21	04/29/21	TP53	L252_I254del	chr17:7577518	NM_000546	-	64.93	nonframeshift	5846	754_762delCTCAC CATC
971	05/12/21	05/17/21	TP53	R273L	chr17:7577120	NM_000546	-	1.18	missense	5671	818G>T
978	04/28/21	04/29/21	TP53	V157F	chr17:7578461	NM_000546	-	26.69	missense	5312	469G>T
979	10/18/21	10/19/21	TP53	R175H	chr17:7578406	NM_000546	-	2.02	missense	3024	524G>A
983	06/16/21	06/21/21	TP53	R181C	chr17:7578389	NM_000546	-	1.15	missense	6435	541C>T
987	10/26/21	10/28/21	TP53	R342*	chr17:7574003	NM_000546	-	0.16	nonsense	5513	1024C>T
1004	05/03/21	05/06/21	MUTYH	Y165C	chr1:45798475	NM_001048171	-	50.15	missense	2026	494A>G
1017	04/29/21	04/30/21	TP53	Y236C	chr17:7577574	NM_000546	-	62.22	missense	3708	707A>G
1021	12/08/21	12/09/21	MRE11A	R605*	chr11:94170372	NM_005590	-	1.36	nonsense	589	1813C>T
1029	05/05/21	05/08/21	ATM	V2923fs*6	chr11:10822458 7	NM_000051	+	50.23	frameshift	4475	8767_8777delGTT GAAGGTGT
1032	05/21/21	05/24/21	TP53	P152L	chr17:7578475	NM_000546	-	5.72	missense	3639	455C>T
1034	04/29/21	04/30/21	FANCL	M1T	chr2:58468447	NM_018062	-	48.28	nonsense	1077	2T>C
1034	04/29/21	04/30/21	BRCA1	A1708E	chr17:41215920	NM_007294	-	51.88	missense	3184	5123C>A
1036	05/10/21	05/13/21	TP53	Q165*	chr17:7578437	NM_000546	-	1.18	nonsense	6863	493C>T
1036	05/10/21	05/13/21	RAD51B	R47*	chr14:68292235	NM_133509	+	50.17	nonsense	897	139C>T
1036	10/07/21	10/12/21	RAD51B	R47*	chr14:68292235	NM_133509		48.32	nonsense	1310	139C>T
1045	05/12/21	05/17/21	TP53	R213*	chr17:7578212	NM_000546	-	31.68	nonsense	3479	637C>T
1066	06/09/21	06/17/21	MUTYH	Y165C	chr1:45798475	NM_001048171	-	51.15	missense	2792	494A>G
1084	05/19/21	05/21/21	TP53	S240R	chr17:7577563	NM_000546	-	0.12	missense	5090	718A>C
1086	05/14/21	05/17/21	TP53	R273H	chr17:7577120	NM_000546	-	26.45	missense	5402	818G>A
1099	05/28/21	06/02/21	TP53	R175H	chr17:7578406	NM_000546	-	18.3	missense	6066	524G>A
1119	08/23/21	08/25/21	TP53	R248W	chr17:7577539	NM_000546	-	5.33	missense	2008	742C>T

1132	06/03/21	06/09/21	TP53	C242F	chr17:7577556	NM_000546	-	1.23	missense	4554	725G>T
1134	06/03/21	06/09/21	TP53	E204*	chr17:7578239	NM_000546	-	4.57	nonsense	5884	610G>T
1135	07/02/21	07/06/21	TP53	R306*	chr17:7577022	NM_000546	-	3.57	nonsense	6546	916C>T
1136	05/16/21	05/20/21	TP53	V173M	chr17:7578413	NM_000546	-	2.26	missense	5921	517G>A
1136	05/16/21	05/20/21	TP53	Q136fs*35	chr17:7578524	NM_000546	-	1.3	frameshift	6010	405_406insGC
1149	05/18/21	05/21/21	TP53	R273H	chr17:7577120	NM_000546	-	3.53	missense	4678	818G>A
1161	06/09/21	06/16/21	TP53	I195T	chr17:7578265	NM_000546	-	3.4	missense	3613	584T>C
1161	12/08/21	12/09/21	TP53	I195T	chr17:7578265	NM_000546	-	9.19	missense	7018	584T>C
1161	01/17/22	01/20/22	TP53	I195T	chr17:7578265	NM_000546	-	11.32	missense	3930	584T>C
1161	06/09/21	06/16/21	ATM	R337H	chr11:108117799	NM_000051	+	3.17	missense	3498	1010G>A
1161	12/08/21	12/09/21	ATM	R337H	chr11:108117799	NM_000051	+	8.15	missense	6110	1010G>A
1161	01/17/22	01/20/22	ATM	R337H	chr11:108117799	NM_000051		11.0	missense	3699	1010G>A
1164	06/02/22	06/07/22	TP53	Y236C	chr17:7577574	NM_000546	-	39.31	missense	2989	707A>G
1173	09/16/21	09/20/21	TP53	Y220C	chr17:7578190	NM_000546	-	2.95	missense	2748	659A>G
1186	05/28/21	06/02/21	TP53	R248W	chr17:7577539	NM_000546	-	2.38	missense	3278	742C>T
1193	05/23/21	06/01/21	TP53	A88fs*32	chr17:7579416	NM_000546	-	37.35	frameshift	5465	261_270delAGCCCCCTCC
1220	04/29/21	04/30/21	TP53	E285K	chr17:7577085	NM_000546	-	43.81	missense	7056	853G>A
1220	04/29/21	04/30/21	BRCA1	S713*	chr17:41245410	NM_007294	-	30.07	nonsense	9509	2138C>G
1223	06/03/21	06/09/21	TP53	L145P	chr17:7578496	NM_000546	-	18.18	missense	6737	434T>C
1225	06/09/21	06/16/21	TP53	R273H	chr17:7577120	NM_000546	-	2.3	missense	6045	818G>A
1234	06/17/21	06/22/21	TP53	R158F	chr17:7578457	NM_000546	-	0.43	missense	7286	472_473CG>TT
1237	06/15/21	06/18/21	TP53	V225fs*22	chr17:7578176	NM_000546	-	1.06	frameshift	1979	672delG
1237	06/15/21	06/18/21	BRCA1	T1677fs*2	chr17:41219665	NM_007294	-	48.97	frameshift	2336	5030_5033delCTAA
1240	06/08/21	06/16/21	TP53	R273H	chr17:7577120	NM_000546	-	9.1	missense	4834	818G>A
1250	04/15/22	04/20/22	BRIP1	A551V	chr17:59858343	NM_032043	-	52.39	missense	794	1652C>T
1260	09/29/21	10/04/21	TP53	A79fs*68	chr17:7579448	NM_000546	-	6.65	frameshift	7788	234_239AGCTCC>T
1261	06/22/21	06/24/21	MUTYH	G382D	chr1:45797228	NM_001048171	-	50.23	missense	1324	1145G>A
1263	07/27/22	07/29/22	TP53	R213fs*34	chr17:7578212	NM_000546	-	2.78	frameshift	5327	636delT

1270	06/01/21	06/09/21	TP53	R306*	chr17:7577022	NM_000546	-	2.22	nonsense	2210	916C>T
1270	06/01/21	06/09/21	FANCA	A958fs*31	chr16:89825093	NM_000135	-	49.36	frameshift	940	2872delG
1272	06/22/22	06/23/22	TP53	Q144*	chr17:7578500	NM_000546	-	0.81	nonsense	2236	430C>T
1273	07/23/21	07/29/21	TP53	R158L	chr17:7578457	NM_000546	-	2.3	missense	7515	473G>T
1273	07/23/21	07/29/21	TP53	R248G	chr17:7577539	NM_000546	-	2.03	missense	6446	742C>G
1273	06/25/21	06/28/21	TP53	R248G	chr17:7577539	NM_000546	-	0.94	missense	8157	742C>G
1273	06/25/21	06/28/21	TP53	R158L	chr17:7578457	NM_000546	-	1.19	missense	9359	473G>T
1278	05/25/21	05/27/21	TP53	R282W	chr17:7577094	NM_000546	-	6.94	missense	4998	844C>T
1292	06/08/21	06/17/21	TP53	C176G	chr17:7578404	NM_000546	-	0.18	missense	5960	526T>G
1298	06/24/21	06/28/21	BRCA2	S37*	chr13:32893256	NM_000059	+	66.11	nonsense	3948	110C>G
1313	06/11/21	06/21/21	TP53	R158P	chr17:7578457	NM_000546	-	0.38	missense	6656	473G>C
1316	05/26/21	05/29/21	TP53	R175G	chr17:7578407	NM_000546	-	0.27	missense	5469	523C>G
1329	09/23/21	09/27/21	TP53	E204*	chr17:7578239	NM_000546	-	1.05	nonsense	5352	610G>T
1338	06/18/21	06/22/21	TP53	R248W	chr17:7577539	NM_000546	-	2.85	missense	3192	742C>T
1363	07/09/21	07/13/21	TP53	R175H	chr17:7578406	NM_000546	-	0.91	missense	4941	524G>A
1384	07/05/21	07/08/21	TP53	R273L	chr17:7577120	NM_000546	-	5.52	missense	3822	818G>T
1390	06/30/21	07/02/21	TP53	splice site 673-2A>T	chr17:7577610	NM_000546	-	0.63	splice	4736	673-2A>T
1392	06/28/21	06/30/21	TP53	R175H	chr17:7578406	NM_000546	-	0.24	missense	4541	524G>A
1402	06/22/21	06/24/21	TP53	G279E	chr17:7577102	NM_000546	-	9.11	missense	5674	836G>A
1402	12/31/21	01/05/22	TP53	G279E	chr17:7577102	NM_000546	-	4.45	missense	3191	836G>A
1407	07/15/21	07/19/21	TP53	R273C	chr17:7577121	NM_000546	-	50.13	missense	3417	817C>T
1417	07/09/21	07/13/21	MUTYH	G382D	chr1:45797228	NM_001048171	-	49.53	missense	1490	1145G>A
1418	06/24/21	06/28/21	TP53	R248Q	chr17:7577538	NM_000546	-	2.39	missense	1886	743G>A
1421	06/24/21	06/28/21	TP53	R248Q	chr17:7577538	NM_000546	-	7.85	missense	4219	743G>A
1421	06/24/21	06/28/21	TP53	G266R	chr17:7577142	NM_000546	-	12.2	missense	5024	795_796GG>TC
1422	06/24/21	06/28/21	TP53	R342*	chr17:7574003	NM_000546	-	21.31	nonsense	3256	1024C>T
1435	08/13/21	08/16/21	TP53	G245S	chr17:7577548	NM_000546	-	0.85	missense	4823	733G>A
1455	07/13/21	07/15/21	TP53	A86fs*55	chr17:7579407	NM_000546	-	0.29	frameshift	5472	257_279del23

1455	07/13/21	07/15/21	BRCA1	R1699Q	chr17:41215947	NM_007294	-	49.68	missense	3967	5096G>A
1457	07/09/21	07/13/21	TP53	W53*	chr17:7579528	NM_000546	-	0.32	nonsense	6201	159G>A
1458	07/01/21	07/06/21	ATM	R2832C	chr11:108216545	NM_000051	+	50.63	missense	4456	8494C>T
1466	07/20/21	07/22/21	TP53	R175H	chr17:7578406	NM_000546	-	6.17	missense	3306	524G>A
1479	05/31/21	06/09/21	TP53	R273C	chr17:7577121	NM_000546	-	0.8	missense	4774	817C>T
1482	07/08/21	07/13/21	RAD51C	A354fs*34	chr17:56811510	NM_058216	+	48.71	frameshift	967	1059_1072delTGCATGTTCATTGC
1482	02/23/22	02/25/22	RAD51C	A354fs*34	chr17:56811510	NM_058216	+	48.05	frameshift	718	1059_1072delTGCATGTTCATTGC
1483	07/29/21	08/02/21	TP53	R290C	chr17:7577070	NM_000546	-	50.65	missense	4713	868C>T
1483	07/29/21	08/02/21	TP53	Y236fs*11	chr17:7577573	NM_000546	-	0.92	frameshift	3584	707delA
1483	07/01/22	07/07/22	TP53	R290C	chr17:7577070	NM_000546	-	61.27	missense	7092	868C>T
1483	07/01/22	07/07/22	TP53	Y236fs*11	chr17:7577573	NM_000546	-	21.95	frameshift	4860	707delA
1494	07/29/21	08/02/21	TP53	R248W	chr17:7577539	NM_000546	-	71.39	missense	3184	742C>T
1509	07/13/21	07/19/21	TP53	R248L	chr17:7577538	NM_000546	-	11.73	missense	3308	743G>T
1509	07/13/21	07/19/21	TP53	R248L	chr17:7577538	NM_000546	-	11.73	missense	3308	743G>T
1528	06/30/21	07/02/21	TP53	R273H	chr17:7577120	NM_000546	-	1.53	missense	4196	818G>A
1537	07/28/21	07/29/21	TP53	C238W	chr17:7577567	NM_000546	-	23.71	missense	2906	714T>G
1549	07/29/21	08/02/21	ATR	E254*	chr3:142281484	NM_001184	-	7.49	nonsense	6797	760G>T
1553	08/05/21	08/09/21	TP53	F113L	chr17:7579348	NM_000546	-	7.69	missense	6162	339C>A
1558	08/05/21	08/09/21	TP53	P278L	chr17:7577105	NM_000546	-	2.41	missense	8223	833C>T
1561	08/05/21	08/10/21	TP53	R213*	chr17:7578212	NM_000546	-	0.17	nonsense	3497	637C>T
1568	07/08/21	07/10/21	TP53	E271K	chr17:7577127	NM_000546	-	0.21	missense	2430	811G>A
1569	07/08/21	07/13/21	TP53	R306*	chr17:7577022	NM_000546	-	4.92	nonsense	3109	916C>T
1572	12/30/21	01/04/22	TP53	R273L	chr17:7577120	NM_000546	-	36.94	missense	5788	818G>T
1587	07/20/21	07/23/21	TP53	R283P	chr17:7577090	NM_000546	-	1.83	missense	5075	848G>C
1609	08/12/21	08/16/21	MSH2	Q348*	chr2:47643534	NM_000251	+	10.28	nonsense	1304	1042C>T
1622	08/31/21	09/07/21	TP53	W146*	chr17:7578492	NM_000546	-	0.74	nonsense	6218	438G>A
1627	10/07/21	10/08/21	TP53	P278R	chr17:7577105	NM_000546	-	8.87	missense	6809	833C>G
1629	01/07/22	01/11/22	TP53	K139fs*31	chr17:7578515	NM_000546	-	4.66	frameshift	7302	414delC

1629	03/08/22	03/11/22	TP53	K139fs*31	chr17:7578515	NM_000546	-	24.24	frameshift	2500	414delC
1629	01/18/22	01/20/22	TP53	K139fs*31	chr17:7578515	NM_000546	-	6.37	frameshift	5321	414delC
1638	08/05/21	08/09/21	TP53	Y234N	chr17:7577581	NM_000546	-	1.71	missense	4394	700T>A
1661	07/16/21	07/21/21	TP53	R273C	chr17:7577121	NM_000546	-	1.06	missense	5292	817C>T
1674	08/30/21	09/02/21	TP53	V173M	chr17:7578413	NM_000546	-	17.15	missense	6375	517G>A
1674	08/30/21	09/02/21	FANCA	V372fs*42	chr16:89858441	NM_000135	-	41.9	frameshift	914	1115_1118delTTG G
1675	09/02/21	09/07/21	TP53	I195T	chr17:7578265	NM_000546	-	4.81	missense	5762	584T>C
1676	08/23/21	08/25/21	TP53	A161D	chr17:7578448	NM_000546	-	2.14	missense	2989	482C>A
1685	08/31/21	09/02/21	FANCG	K618fs*3	chr9:35074120	NM_004629	-	48.54	frameshift	1304	1852_1853delAA
1694	06/30/21	07/02/21	TP53	C176F	chr17:7578403	NM_000546	-	22.09	missense	4337	527G>T
1694	06/30/21	07/02/21	BARD1	G17fs*37	chr2:215674231	NM_000465	-	43.53	frameshift	765	50_62delGGAACG AGCCTCG
1696	09/14/21	09/17/21	TP53	E198*	chr17:7578257	NM_000546	-	6.12	nonsense	4036	592G>T
1702	08/31/21	09/02/21	TP53	R248Q	chr17:7577538	NM_000546	-	11.43	missense	4138	743G>A
1702	03/15/22	03/16/22	TP53	R248Q	chr17:7577538	NM_000546	-	38.58	missense	3525	743G>A
1702	05/02/22	05/03/22	TP53	R248Q	chr17:7577538	NM_000546	-	51.73	missense	2915	743G>A
1706	08/26/21	08/30/21	TP53	R249M	chr17:7577535	NM_000546	-	11.32	missense	2527	746G>T
1706	08/26/21	08/30/21	TP53	P72fs*77	chr17:7579470	NM_000546	-	11.4	frameshift	3202	215_217CCG>GC GT
1716	08/24/21	08/26/21	TP53	R248Q	chr17:7577538	NM_000546	-	1.02	missense	6593	743G>A
1724	02/16/22	02/18/22	BRCA1	Q1756fs*74	chr17:41209079	NM_007294	-	49.58	frameshift	5653	5266_5267insC
1730	06/18/21	06/22/21	TP53	R342*	chr17:7574003	NM_000546	-	18.12	nonsense	4703	1024C>T
1730	06/18/21	06/22/21	TP53	S127P	chr17:7578551	NM_000546	-	24.6	missense	5273	379T>C
1742	08/19/21	08/23/21	TP53	V157F	chr17:7578461	NM_000546	-	6.06	missense	5972	469G>T
1745	08/19/21	08/23/21	TP53	H193Y	chr17:7578272	NM_000546	-	59.93	missense	3891	577C>T
1752	08/23/21	08/26/21	TP53	V217G	chr17:7578199	NM_000546	-	0.81	missense	2348	650T>G
1763	08/24/21	08/27/21	TP53	Q317*	chr17:7576897	NM_000546	-	5.59	nonsense	6905	949C>T
1777	08/26/21	08/30/21	TP53	C135F	chr17:7578526	NM_000546	-	30.61	missense	5159	404G>T
1787	09/13/21	09/16/21	TP53	P152T	chr17:7578476	NM_000546	-	12.89	missense	6859	454C>A
1821	08/27/21	08/31/21	ATM	H2195fs*5	chr11:10819604 8	NM_000051	+	49.14	frameshift	1736	6585_6586delTA

1822	09/01/21	09/07/21	TP53	M40fs*4	chr17:7579568	NM_000546	-	35.65	frameshift	3338	118delA
1842	07/08/21	07/13/21	TP53	V157fs*1	chr17:7578462	NM_000546	-	5.93	frameshift	6140	467_468insTTGATTCCACACCCCCGCCCGGCACCCG
1863	09/14/21	09/17/21	TP53	R175H	chr17:7578406	NM_000546	-	5.01	missense	3874	524G>A
1871	09/14/21	09/17/21	TP53	P27fs*20	chr17:7579717	NM_000546	-	0.8	frameshift	2240	79C>GTTTTCAGA
1894	09/16/21	09/21/21	TP53	H179R	chr17:7578394	NM_000546	-	0.53	missense	2462	536A>G
1899	09/14/21	09/17/21	TP53	G245D	chr17:7577547	NM_000546	-	25.18	missense	3372	734G>A
1899	10/11/21	10/12/21	TP53	G245D	chr17:7577547	NM_000546	-	9.19	missense	3503	734G>A
1899	09/14/21	09/17/21	MUTYH	G382D	chr1:45797228	NM_001048171	-	100	missense	1152	1145G>A
1899	10/11/21	10/12/21	MUTYH	G382D	chr1:45797228	NM_001048171	-	100.0	missense	1310	1145G>A
1932	09/20/21	09/24/21	TP53	C238Y	chr17:7577568	NM_000546	-	30.25	missense	2238	713G>A
1936	09/22/21	09/27/21	TP53	G266V	chr17:7577141	NM_000546	-	0.13	missense	5389	797G>T
1944	09/23/21	09/27/21	TP53	Y126C	chr17:7578553	NM_000546	-	34.37	missense	3878	377A>G
1947	04/07/22	04/12/22	TP53	splice site 783-1G>A	chr17:7577156	NM_000546	-	47.9	splice	3493	783-1G>A
1954	01/31/22	02/02/22	TP53	R175H	chr17:7578406	NM_000546	-	1.35	missense	5259	524G>A
1954	10/21/21	10/22/21	TP53	R175H	chr17:7578406	NM_000546	-	0.33	missense	7327	524G>A
1956	09/30/21	10/04/21	TP53	R282W	chr17:7577094	NM_000546	-	4.25	missense	4474	844C>T
1957	09/30/21	10/04/21	TP53	C242F	chr17:7577556	NM_000546	-	0.63	missense	4266	725G>T
1957	06/24/22	06/28/22	TP53	C242F	chr17:7577556	NM_000546	-	36.79	missense	4808	725G>T
1958	09/30/21	10/04/21	TP53	N239fs*25	chr17:7577564	NM_000546	-	4.64	frameshift	3792	716_717insA
1958	09/30/21	10/04/21	TP53	W53fs*70	chr17:7579527	NM_000546	-	0.18	frameshift	4404	159delG
1960	09/30/21	10/05/21	TP53	S127F	chr17:7578550	NM_000546	-	9.26	missense	4514	380C>T
1961	09/30/21	10/04/21	MUTYH	Y165C	chr1:45798475	NM_001048171	-	52.97	missense	2343	494A>G
1961	09/30/21	10/04/21	ATM	S864fs*1	chr11:108138020	NM_000051	+	0.18	frameshift	7268	2589_2590insT
1965	09/20/21	09/24/21	BRCA2	A938fs*21	chr13:32911299	NM_000059	+	47.72	frameshift	5568	2808_2811delACAA
1979	10/07/21	10/08/21	TP53	Y220C	chr17:7578190	NM_000546	-	1.99	missense	4023	659A>G
1995	09/14/21	09/17/21	TP53	A276fs*29	chr17:7577111	NM_000546	-	0.47	frameshift	5518	825_826delTG
1997	09/27/21	09/30/21	TP53	S215I	chr17:7578205	NM_000546	-	10.18	missense	1925	644G>T

1998	10/05/21	10/06/21	TP53	F134L	chr17:7578528	NM_000546	-	28.59	missense	3603	402T>G
2013	10/07/21	10/12/21	TP53	Y220C	chr17:7578190	NM_000546	-	39.48	missense	3541	659A>G
2014	10/07/21	10/12/21	TP53	L330P	chr17:7576857	NM_000546	-	16.85	missense	6973	989T>C
2019	10/04/21	10/05/21	TP53	I195T	chr17:7578265	NM_000546	-	23.44	missense	5743	584T>C
2020	10/04/21	10/05/21	TP53	I255F	chr17:7577518	NM_000546	-	2.44	missense	3806	763A>T
2052	10/11/21	10/12/21	TP53	C238Y	chr17:7577568	NM_000546	-	18.62	missense	3819	713G>A
2065	10/18/21	10/19/21	MUTYH	G382D	chr1:45797228	NM_001048171	-	48.96	missense	1250	1145G>A
2113	07/21/22	07/22/22	TP53	R267fs*5	chr17:7577138	NM_000546	-	26.79	frameshift	4644	799_800insC
2120	09/24/21	09/27/21	TP53	Q375*	chr17:7572986	NM_000546	-	6.55	nonsense	4335	1123C>T
2131	06/21/22	06/24/22	TP53	H193Y	chr17:7578272	NM_000546	-	3.82	missense	2591	577C>T
2138	10/01/21	10/05/21	BRCA2	K437fs*22	chr13:32906924	NM_000059	+	50.59	frameshift	4418	1310_1313delAAGA
2151	09/01/21	09/07/21	TP53	C124fs*46	chr17:7579316	NM_000546	-	22.39	frameshift	2831	370delT
2151	09/01/21	09/07/21	TP53	C124fs*46	chr17:7579316	NM_000546	-	22.39	frameshift	2831	370delT
2162	10/21/21	10/26/21	ATM	I326fs*3	chr11:108117765	NM_000051		50.13	frameshift	3026	977_978delTA
2171	09/22/21	09/27/21	TP53	Y220C	chr17:7578190	NM_000546	-	61.28	missense	4615	659A>G
2181	06/15/22	06/17/22	ATM	splice site 6453-1_6457delGAGTAA	chr11:108192026	NM_000051		52.08	splice	2959	6453-1_6457delGAGTAA
2187	10/13/21	10/15/21	TP53	splice site 375G>A	chr17:7579312	NM_000546	-	0.64	splice	3569	375G>A
2199	12/31/21	01/05/22	TP53	R213*	chr17:7578212	NM_000546	-	19.03	nonsense	3510	637C>T
2207	10/27/21	11/02/21	MLH1	Y690*	chr3:37090475	NM_000249		5.58	nonsense	1576	2070C>G
2207	10/27/21	11/02/21	BRCA2	V1283fs*2	chr13:32912338	NM_000059		54.71	frameshift	3007	3847_3848delGT
2217	11/03/21	11/05/21	TP53	splice site 375G>A	chr17:7579312	NM_000546	-	19.14	splice	2487	375G>A
2221	10/29/21	11/04/21	TP53	V272L	chr17:7577124	NM_000546	-	0.68	missense	5698	814G>C
2221	10/29/21	11/04/21	TP53	H179R	chr17:7578394	NM_000546	-	0.67	missense	7317	536A>G
2227	10/27/21	11/02/21	TP53	R333fs*12	chr17:7574029	NM_000546	-	3.0	frameshift	2000	997delC
2231	11/04/21	11/09/21	TP53	P152L	chr17:7578475	NM_000546	-	1.32	missense	5092	455C>T
2239	10/20/21	10/21/21	TP53	R248Q	chr17:7577538	NM_000546	-	12.43	missense	3378	743G>A
2296	11/24/21	11/26/21	TP53	R273C	chr17:7577121	NM_000546	-	42.55	missense	2571	817C>T

2303	10/25/21	10/26/21	TP53	I232S	chr17:7577586	NM_000546	-	30.17	missense	3947	695T>G
2303	10/25/21	10/26/21	ATR	A669fs*25	chr3:142275297	NM_001184	-	10.29	frameshift	5005	2005delG
2303	10/25/21	10/26/21	ATM	R2263*	chr11:10819625 1	NM_000051		8.97	nonsense	4313	6787A>T
2316	10/14/21	10/19/21	ATR	R2407H	chr3:142178198	NM_001184	-	48.87	missense	5834	7220G>A
2348	02/03/22	02/04/22	TP53	Y236C	chr17:7577574	NM_000546	-	2.15	missense	4004	707A>G
2372	11/18/21	11/22/21	TP53	G244S	chr17:7577551	NM_000546	-	35.93	missense	2786	730G>A
2416	11/10/21	11/16/21	TP53	R273C	chr17:7577121	NM_000546	-	10.62	missense	4936	817C>T
2419	11/15/21	11/17/21	TP53	L194R	chr17:7578268	NM_000546	-	8.41	missense	3176	581T>G
2419	11/15/21	11/17/21	ATRX	A386fs*29	chrX:76939591	NM_000489	-	12.33	frameshift	1363	1156_1157insTG
2455	11/23/21	11/25/21	TP53	S240R	chr17:7577561	NM_000546	-	12.96	missense	3720	720T>G
2458	12/13/21	12/15/21	TP53	splice site 920-1G>T	chr17:7576927	NM_000546	-	0.94	splice	7467	920-1G>T
2459	11/25/21	11/29/21	TP53	Q331fs*14	chr17:7576855	NM_000546	-	16.55	frameshift	6216	990delT
2461	11/25/21	11/29/21	TP53	splice site 673-9_676delATCTCCTAGGTTG	chr17:7577604	NM_000546	-	0.67	splice	3854	673-9_676delATCTCCTAGGTTG
2462	11/25/21	11/30/21	TP53	I254S	chr17:7577520	NM_000546	-	2.1	missense	2859	761T>G
2468	11/23/21	11/25/21	TP53	I254S	chr17:7577520	NM_000546	-	0.16	missense	3680	761T>G
2477	12/07/21	12/08/21	TP53	R213L	chr17:7578211	NM_000546	-	18.01	missense	4119	638G>T
2485	10/29/21	11/04/21	PARP3	R47C	chr3:51977510	NM_005485	+	49.77	missense	1278	139C>T
2507	12/14/21	12/15/21	FANCC	P282fs*30	chr9:97897627	NM_000136	-	45.68	frameshift	950	840_843delGCTG
2523	10/05/21	10/07/21	TP53	R273H	chr17:7577120	NM_000546	-	0.96	missense	6962	818G>A
2592	11/23/21	11/24/21	MUTYH	G382D	chr1:45797228	NM_001048171	-	51.73	missense	1475	1145G>A
2610	12/16/21	12/20/21	TP53	C141Y	chr17:7578508	NM_000546	-	2.4	missense	4203	422G>A
2612	12/16/21	12/20/21	TP53	R273L	chr17:7577120	NM_000546	-	6.95	missense	5566	818G>T
2617	12/09/21	12/13/21	TP53	R175H	chr17:7578406	NM_000546	-	0.23	missense	6123	524G>A
2631	12/28/21	12/29/21	TP53	V157F	chr17:7578461	NM_000546	-	1.03	missense	5545	469G>T
2651	12/24/21	12/28/21	ATM	D2721N	chr11:10820658 1	NM_000051	+	5.77	missense	4953	8161G>A
2670	12/24/21	12/28/21	TP53	G279E	chr17:7577102	NM_000546	-	50.31	missense	2542	836G>A
2689	01/05/22	01/07/22	ATM	E2039K	chr11:10818675 7	NM_000051	+	69.42	missense	4330	6115G>A

2726	12/02/21	12/06/21	TP53	Q167fs*3	chr17:7578428	NM_000546	-	7.12	frameshift	8973	501delG
2726	12/02/21	12/06/21	BRCA1	splice site 302-1G>A	chr17:41256279	NM_007294	-	3.64	splice	4509	302-1G>A
2800	12/28/21	12/29/21	TP53	R248W	chr17:7577539	NM_000546	-	47.72	missense	2504	742C>T
2802	12/31/21	01/07/22	TP53	P250L	chr17:7577532	NM_000546	-	10.83	missense	3960	749C>T
2803	12/17/21	12/20/21	MUTYH	G382D	chr1:45797228	NM_001048171	-	51.03	missense	1260	1145G>A
2955	01/14/22	01/17/22	TP53	E336*	chr17:7574021	NM_000546	-	1.1	nonsense	4526	1006G>T
2955	01/14/22	01/17/22	BRCA1	G424fs*6	chr17:41246276	NM_007294	-	1.91	frameshift	7242	1271delG

**Tissue biopsy-only mutations**			
ID	Collection date ?	Gene ?	Alteration
16	02/09/21	TP53	V172F
35	03/02/21	TP53	A138V
36	04/20/21	TP53	splice site 375+1G>A
36	04/20/21	FANCA	Q1307*
39	03/15/21	TP53	splice site 559+1G>T
50	02/09/21	FANCA	E198*
57	05/30/16	TP53	P47fs*76
57	05/30/16	TP53	R342*
57	05/30/16	MSH6	F1088fs*2
87	04/30/21	TP53	splice site 993+1G>T
90	12/05/19	TP53	Y163N
97	09/27/17	TP53	Y220C
101	11/27/20	TP53	Q331*
118	06/16/20	TP53	R280G
124	06/02/22	TP53	R158H
138	02/25/21	TP53	R283P
155	05/21/21	TP53	splice site 1101-1G>A

167	12/04/14	TP53	E204*
167	12/04/14	RAD51D	L75fs*26
167	12/04/14	ATM	splice site 6976-2A>G
183	02/09/21	NBN	R466fs*18
184	02/01/21	TP53	splice site 375G>T
185	01/12/21	TP53	V157F
186	01/19/21	TP53	splice site 783-1G>T
195	02/01/21	TP53	E286* - subclonal
206	01/20/21	TP53	splice site 375G>A
218	03/29/21	TP53	Q38*
232	02/01/21	TP53	E171*
232	02/01/21	MSH3	K383fs*32
232	02/01/21	MLH1	S698*
242	12/17/20	TP53	R158S
242	12/17/20	PALB2	F606fs*10
253	01/21/21	PMS2	E5*
256	02/02/21	TP53	S215G
259	01/20/21	TP53	G105V
262	02/04/21	TP53	R248Q
264	03/30/21	TP53	R175H
266	03/04/21	TP53	R248Q
266	03/04/21	TP53	G105D
271	02/02/21	TP53	R213*
289	01/26/21	TP53	splice site 919+1G>A
293	02/19/21	MSH6	S602*
294	03/10/21	MLH1	P654L
294	03/10/21	ATM	splice site 3747-1G>T
296	02/05/21	BRIP1	splice site 2906-1G>C

298	01/29/21	MSH6	F1088fs*2
298	01/29/21	MSH3	K383fs*32
298	01/29/21	BRCA2	N1822fs*18
298	01/29/21	ATM	G204*
300	03/19/21	TP53	R248P
312	03/16/21	TP53	Y220C
330	02/15/21	TP53	E258A
332	03/12/21	TP53	splice site 375+1G>C
346	03/03/21	ATR	R2547*
354	12/23/21	TP53	R181P
358	03/09/21	TP53	C135Y
360	03/04/21	TP53	P250L
360	03/04/21	TP53	R248W
360	03/04/21	TP53	S241F
360	03/04/21	TP53	W91*
360	03/04/21	RAD51D	splice site 323+1G>A
360	03/04/21	MRE11A	R400fs*3
360	03/04/21	ATM	Q1276*
362	02/08/21	FANCC	R433fs*14
362	02/08/21	ATM	K2811fs*46
366	01/25/21	TP53	splice site 672G>A
401	01/12/21	TP53	splice site 559+1G>A
409	08/26/11	TP53	C135fs*35
444	04/09/21	TP53	splice site 375G>A
453	09/12/19	TP53	E286K
461	01/30/19	TP53	P191del
461	01/30/19	MSH3	K383fs*32
461	01/30/19	MLH1	splice site 454-2A>G

461	01/30/19	BARD1	D172fs*40
461	01/30/19	ATM	K2811fs*46
476	01/25/21	TP53	E285K
484	08/06/21	ATR	I774fs*5
518	01/08/21	TP53	R306*
527	07/28/20	TP53	R273C
536	01/18/22	TP53	H193Y
541	01/24/20	TP53	R249T
560	08/03/20	TP53	R280K
568	11/23/18	TP53	F134L
650	11/24/20	TP53	H193Y
675	06/30/20	TP53	G266E
687	05/12/20	TP53	L348_E349>F*
718	02/15/18	TP53	V274F
764	06/30/17	TP53	splice site 375G>T
764	06/30/17	BRCA2	splice site 316+2T>G
789	01/02/20	TP53	V203L
789	01/02/20	NBN	splice site 1915-1G>A
789	01/02/20	MLH1	splice site 453+1G>A
789	01/02/20	ATRX	K1485fs*1
825	06/10/20	TP53	Y234C
845	12/27/19	TP53	R213W
863	04/14/21	TP53	splice site 994-1G>T
908	04/27/21	TP53	R273H
912	04/21/21	TP53	R342*
962	12/09/20	BRCA2	splice site 9257-2A>G
971	01/14/21	TP53	splice site 782+1G>T
978	04/28/21	TP53	splice site 994-1G>A

983	06/16/21	TP53	P152L
983	06/16/21	MSH6	F1088fs*5
1009	05/25/21	TP53	K321fs*24
1009	05/25/21	TP53	M246R
1009	05/25/21	ATM	K2302fs*69
1022	04/28/21	MSH2	splice site 645+1G>A
1034	02/12/15	TP53	R175H
1072	05/06/21	MUTYH	W395*
1093	05/12/21	TP53	R273C
1130	02/07/20	TP53	R175H
1148	05/20/21	CHEK2	splice site 444+1G>A
1174	06/09/21	TP53	I255T
1190	01/27/21	TP53	R175H
1214	06/25/21	TP53	C135Y
1229	10/01/18	TP53	C275F
1240	05/17/21	FANCL	splice site 472-1G>C
1250	04/15/22	TP53	E171*
1261	06/22/21	FANCG	Y551fs*7
1287	10/04/21	ATR	splice site 6552+1G>T
1330	05/26/21	TP53	R65*
1371	06/23/21	TP53	R175H
1371	06/23/21	MSH6	P982fs*13
1384	04/19/21	ATRX	S566fs*9
1396	02/19/21	TP53	splice site 376-1G>A
1408	02/25/20	TP53	P191del
1410	12/03/19	TP53	splice site 919+1G>C
1415	06/12/18	TP53	splice site 375+5G>T
1432	10/12/21	TP53	Y234C

1432	10/12/21	RAD51C	R237*
1436	09/17/13	TP53	splice site 375G>T
1456	04/26/19	TP53	R273C
1469	07/08/21	ATR	splice site 7656-1G>C
1472	06/08/21	TP53	Y126C
1475	06/21/21	TP53	Q317*
1479	04/30/21	MLH1	splice site 1038+2T>G
1485	06/29/21	TP53	G199A
1485	06/29/21	TP53	R248W
1494	12/14/21	TP53	Y220C
1496	09/08/17	TP53	R282W
1501	03/10/20	TP53	R249M
1501	03/10/20	TP53	R282P
1528	05/15/19	TP53	P151H
1541	04/02/19	TP53	R273L
1541	04/02/19	TP53	E180fs*23
1552	07/02/21	TP53	R248Q
1552	07/02/21	ATRX	G1368fs*7
1553	11/04/20	TP53	splice site 375+5G>T
1566	06/04/21	FANCC	R433fs*14
1666	09/03/21	TP53	R282W
1679	07/05/21	TP53	splice site 783-2A>G
1694	06/30/21	TP53	E285K
1724	02/16/22	POLE	D1955N
1739	04/20/15	BRCA2	splice site 8754G>A
1759	02/22/21	TP53	P152L
1779	07/13/17	TP53	R248W
1822	04/20/21	ATR	M2058fs*6

1841	01/11/21	TP53	C176W
1853	01/12/21	TP53	splice site 994-2A>C
1868	12/21/20	TP53	R175H
1872	04/24/19	TP53	V73fs*76
1883	09/14/21	TP53	A138P
1901	05/31/21	TP53	R110fs*15
1910	01/28/21	TP53	Y163C
1932	07/01/21	ATRX	S523fs*39
1937	05/17/21	TP53	splice site 375G>T
1942	11/30/21	TP53	R273H
1961	11/02/18	ATM	S2812fs*3
1991	09/30/21	TP53	R248Q
1997	09/14/21	BRCA1	Q210*
2011	10/11/19	TP53	R249S
2018	08/18/21	TP53	H193Y
2045	08/10/21	TP53	G266R
2065	10/13/21	FANCA	FANCA-FANCA truncation
2069	08/16/21	RAD51B	loss
2111	11/20/18	TP53	G245S
2124	08/28/21	TP53	C238Y
2124	08/28/21	TP53	R273L
2162	11/25/20	TP53	G279R
2166	03/11/21	TP53	P151H
2169	09/02/21	TP53	splice site 97-5_101delTACAGTCCCC
2208	09/15/21	TP53	P128fs*42
2232	07/09/21	TP53	A159fs*8
2232	07/09/21	TP53	R342*

2232	07/09/21	TP53	V157L
2232	07/09/21	TP53	R248W
2232	07/09/21	TP53	R158G
2316	10/15/21	TP53	R342*
2347	03/03/22	ATM	E1514*
2374	09/23/15	TP53	Q192*
2374	09/23/15	ERCC4	R799W
2416	07/13/20	BRCA1	BRCA1 truncation
2428	11/20/20	TP53	splice site 375+5G>T
2439	12/02/21	TP53	splice site 97-2A>G
2462	09/11/19	RAD51B	loss
2470	12/06/16	TP53	Y220H
2478	12/06/21	ATM	R2034*
2529	12/14/21	TP53	splice site 920-2A>G
2590	02/09/22	TP53	splice site 991_993+5delC AGGTACT
2610	06/02/20	ATM	R337H
2635	05/13/20	TP53	V272_R273insL
2635	05/13/20	TP53	P278L
2665	11/10/21	ATM	loss
2667	10/06/21	TP53	R175H
2672	02/01/22	TP53	Q331*
2672	02/01/22	TP53	K132N
2697	07/18/19	TP53	loss
2730	11/09/21	ATM	splice site 2921+1G>A
2757	08/18/22	TP53	R273H
2876	06/07/22	CHEK2	loss

Supplementary Table S3

Characteristics of confirmed mutations in whole blood

ID	Gene	p.	c.	VAF Liquid biopsy (%)	dp whole blood (x)	dp alt whole blood (x)	VAF whole blood (%)	Panel	Mean VAF background noise (%)	Sd background noise	VAF threshold m+2sd (%)	Confirmed in whole blood
29	BRCA2	R2888H	8663G>A	0.24	3266	4	0.12	Panel 2	0.05	0.11	0.28	NO
29	TP53	N131del	393_395delCAA	0.25	1758	6	0.30	Panel 2	0.00	0.00	0.00	YES
29	TP53	I195T	584T>C	0.22	4149	11	0.27	Panel 2	0.03	0.03	0.09	YES
38	TP53	E336*	1006G>T	5.3	2074	1	0.05	Panel 2	0.02	0.10	0.23	NO
57	PALB2	Q260*	778C>T	0.13	747	1	0.13	Panel 1	0.03	0.25	0.54	NO
57	TP53	V173M	517G>A	5.65	750	0	0.00	Panel 1	0.00	0.00	0.00	NO
57	TP53	R273C	817C>T	60.83	854	0	0.00	Panel 1	0.00	0.00	0.00	NO
57	PALB2	R1086*	3256C>T	0.53	445	2	0.45	Panel 1	0.02	0.20	0.41	YES
87	ATM	G2891D	8672G>A	0.26	1013	4	0.40	Panel 2	0.02	0.12	0.26	YES
89	ATM	G2891D	8672G>A	0.72	2476	23	0.93	Panel 2	0.02	0.12	0.26	YES
93	TP53	F113V	337T>G	0.27	2247	0	0.00	Panel 2	0.00	0.00	0.00	NO
93	TP53	L289P	866T>C	0.93	4285	1	0.02	Panel 2	0.14	0.12	0.37	NO
93	TP53	E258D	774A>C	0.46	2113	2	0.09	Panel 2	0.01	0.03	0.06	YES
147	ATM	R337C	1009C>T	0.24	1206	1	0.08	Panel 2	0.00	0.04	0.08	YES
147	ATM	R1898*	5692C>T	4.7	1031	46	4.46	Panel 2	0.05	0.17	0.38	YES
147	ATM	S2860del	8578_8580delTCT	0.48	1037	6	0.58	Panel 2	0.00	0.00	0.00	YES
147	ATM	N2985fs*21	8955delT	0.19	1384	0	0.00	Panel 2	0.02	0.13	0.27	NO
147	TP53	Y163C	488A>G	2.5	787	18	2.29	Panel 2	0.14	0.35	0.83	YES
184	CHEK2	T383P	1147A>C	0.69	1261	4	0.32	Panel 2	0.01	0.01	0.03	YES
190	BRCA1	K339fs*2	1016delA	0.78	1118	1	0.09	Panel 1	0.13	0.13	0.38	NO
190	MSH6	F1088fs*5	3261_3262insC	6.69	950	0	0.00	Panel 1	0.00	0.00	0.00	NO
190	TP53	I195T	584T>C	0.14	932	2	0.21	Panel 1	0.00	0.00	0.00	YES
201	CHEK2	S210fs*3	629_633delCAGTT	0.3	399	2	0.50	Panel 1	0.00	0.00	0.00	YES
242	TP53	N131S	392A>G	0.56	598	6	1.00	Panel 1	0.03	0.18	0.38	YES

264	TP53	H214R	641A>G	7.57	653	18	2.80	Panel 1	0.02	0.15	0.32	YES
320	ATM	G2891D	8672G>A	1.6	1325	33	2.49	Panel 2	0.02	0.12	0.26	YES
320	TP53	N239S	716A>G	0.18	776	2	0.26	Panel 2	0.10	0.05	0.20	YES
332	TP53	R248W	742C>T	0.38	661	0	0.00	Panel 1	0.03	0.24	0.52	NO
333	TP53	Y234F	701A>T	0.25	2973	1	0.03	Panel 2	0.02	0.02	0.07	NO
348	CHEK2	P522fs*3	1563_1564ins G	1.76	601	2	0.33	Panel 1	0.00	0.00	0.00	YES
366	TP53	C275Y	824G>A	0.24	6717	11	0.16	Panel 2	0.04	0.16	0.37	NO
366	TP53	D281Y	841G>T	0.26	6985	44	0.63	Panel 2	0.00	0.00	0.00	YES
366	TP53	D281V	842A>T	0.24	6990	32	0.46	Panel 2	0.00	0.00	0.00	YES
381	TP53	P34fs*2	100_124del25	26.91	4347	0	0.00	Panel 1	0.00	0.00	0.00	NO
383	ATM	?	splice site 5178-1G>A	0.57	3341	0	0.00	Panel 2	0.03	0.03	0.10	NO
383	CHEK1	W404*	1212G>A	0.64	3172	0	0.00	Panel 2	0.01	0.03	0.07	NO
383	MLH1	T117M	350C>T	0.67	2686	0	0.00	Panel 2	0.08	0.05	0.19	NO
383	TP53	E258K	772G>A	0.49	1137	0	0.00	Panel 2	0.57	1.66	3.89	NO
383	TP53	H193R	578A>G	0.73	2051	13	0.63	Panel 2	0.06	0.06	0.19	YES
383	TP53	Y205C	614A>G	0.38	3771	14	0.37	Panel 2	0.03	0.04	0.11	YES
383	TP53	N239S	716A>G	0.36	2425	8	0.33	Panel 2	0.10	0.05	0.20	YES
414	ATM	E73fs*26	217_218delGA	0.23	2246	0	0.00	Panel 2	0.00	0.00	0.00	NO
414	TP53	C242S	725G>C	0.2	4922	2	0.04	Panel 2	0.01	0.02	0.05	NO
425	TP53	R273C	817C>T	14.3	4993	5	0.10	Panel 2	0.05	0.04	0.13	NO
544	ATM	R337C	1009C>T	1.51	521	4	0.77	Panel 1	0.03	0.09	0.20	YES
544	ATM	Y2371fs*1	7112_7113ins A	0.27	531	6	1.13	Panel 1	0.00	0.00	0.00	YES
544	ATM	?	splice site 662+1G>T	1.88	531	15	2.82	Panel 1	0.07	0.12	0.32	YES
544	CHEK2	?	splice site 1096-1G>A	2.58	383	6	1.57	Panel 1	0.00	0.00	0.00	YES
544	CHEK2	?	splice site 909-1G>A	0.43	576	3	0.52	Panel 1	0.00	0.00	0.00	YES
544	CHEK2	Y404C	1211A>G	0.5	416	0	0.00	Panel 1	0.00	0.00	0.00	NO
544	TP53	S215R	645T>G	1.62	643	6	0.93	Panel 1	0.61	0.39	1.40	NO
544	TP53	N239S	716A>G	0.31	686	0	0.00	Panel 1	0.00	0.00	0.00	NO

544	TP53	M246V	736A>G	0.2	805	1	0.12	Panel 1	0.02	0.08	0.18	NO
544	TP53	I195T	584T>C	0.48	629	1	0.16	Panel 1	0.00	0.00	0.00	YES
544	TP53	A276P	826G>C	1.51	749	16	2.14	Panel 1	0.00	0.00	0.00	YES
583	TP53	R273H	818G>A	0.39	5060	33	0.65	Panel 2	0.04	0.02	0.09	YES
633	TP53	L194R	581T>G	0.29	6323	11	0.17	Panel 2	0.02	0.02	0.05	YES
633	TP53	R273H	818G>A	0.51	4323	20	0.46	Panel 2	0.04	0.02	0.09	YES
898	TP53	Y205D	613T>G	0.17	768	3	0.39	Panel 1	0.12	0.15	0.42	NO
898	TP53	R248Q	743G>A	0.39	1069	0	0.00	Panel 1	0.00	0.00	0.00	NO
1119	FANCA	?	splice site 1360-1G>A	0.56	1057	15	1.42	Panel 2	0.02	0.02	0.07	YES
1119	TP53	I195T	584T>C	2.7	2196	60	2.73	Panel 2	0.03	0.03	0.09	YES
1119	TP53	P278A	832C>G	0.25	2382	10	0.42	Panel 2	0.03	0.02	0.07	YES
1134	TP53	P190L	569C>T	0.18	1862	11	0.59	Panel 2	0.20	0.08	0.36	YES
1179	TP53	R248Q	743G>A	0.35	848	2	0.24	Panel 1	0.00	0.00	0.00	YES
1272	TP53	R280G	838A>G	6.85	783	0	0.00	Panel 1	0.03	0.30	0.63	NO
1292	TP53	Q38*	112C>T	0.48	556	0	0.00	Panel 1	0.02	0.26	0.55	NO
1421	TP53	?	splice site 560-1G>A	0.56	595	4	0.67	Panel 1	0.02	0.05	0.13	YES
1629	ATM	P2648fs*12	7943delC	0.18	2434	10	0.41	Panel 2	0.00	0.00	0.00	YES
1638	ATM	R3008C	9022C>T	3.54	665	27	4.06	Panel 1	0.03	0.06	0.15	YES
1740	ATM	R337C	1009C>T	0.36	554	1	0.18	Panel 1	0.03	0.09	0.20	NO
1819	ATM	V1729fs*20	5184_5185insA	0.25	507	3	0.59	Panel 1	0.00	0.00	0.00	YES
1991	TP53	N131I	392A>T	0.57	1071	1	0.09	Panel 2	0.06	0.05	0.17	NO

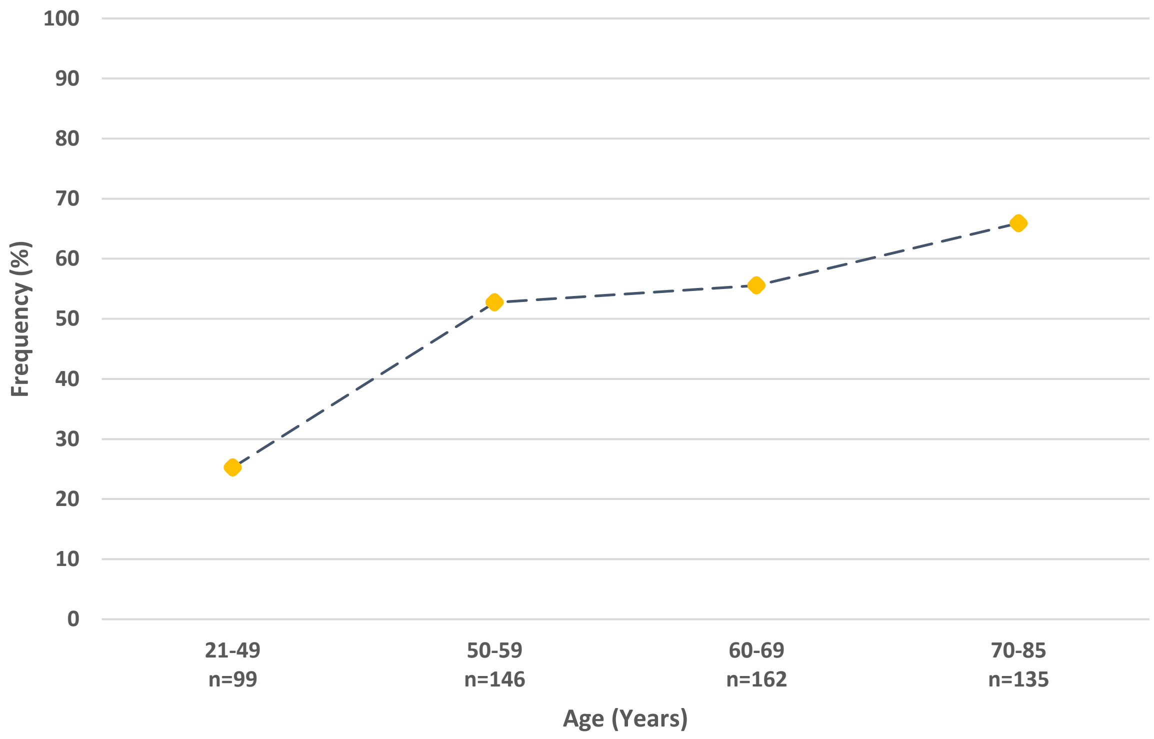

Supplementary Figure S1

Percentage of patients with at least one *liquid biopsy-only mutations* by age

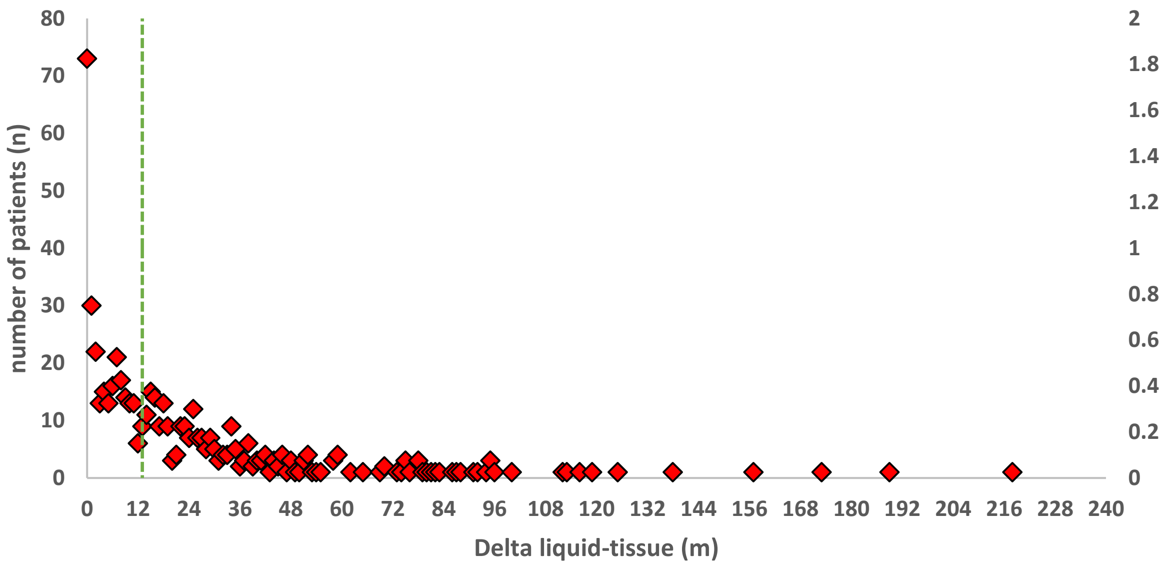

Supplementary Figure S2

Distribution of the time difference (delta) between tissue and liquid samples in months. More than 50% of the liquid samples were collected within a period of less than 13 months after the corresponding tissue samples.

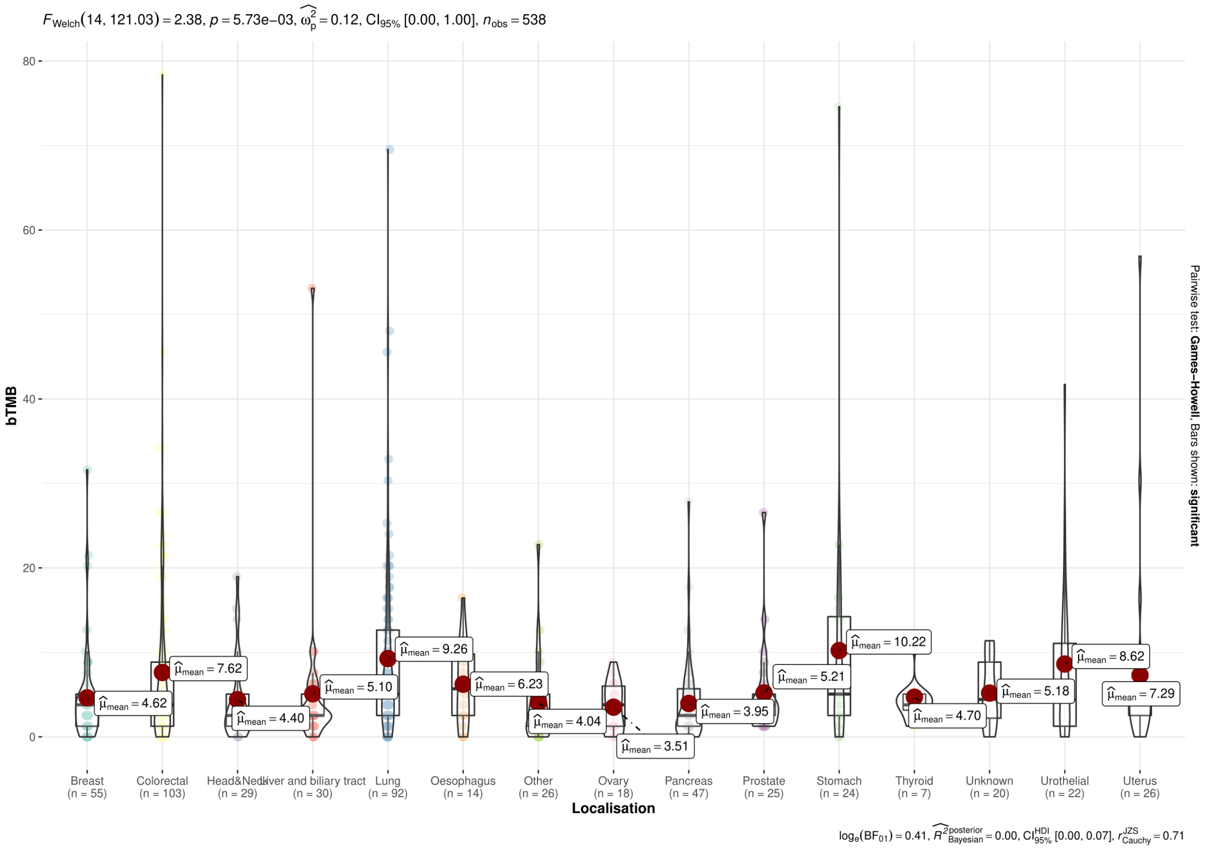

Supplementary Figure S3

Boxplot representing the different bTMB across the different cancer types after elimination of 4 outliers values.

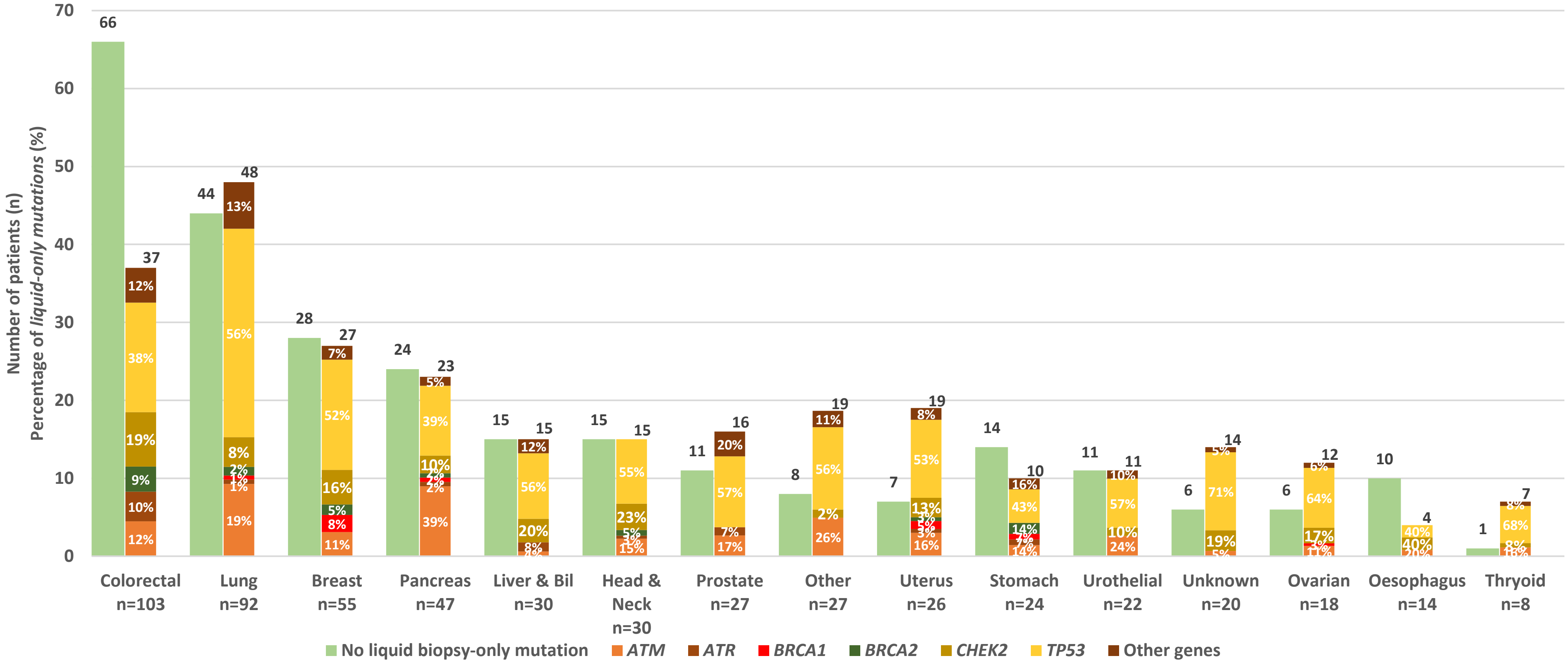

Supplementary Figure S4

For each localization, the height of the left bar on the histogram represents the number of patients without any *liquid biopsy-only mutations* and the height of the right bar represents the number of patients with at least one *liquid biopsy-only mutations*.

The right clustered bar is segmented proportionally to the distribution of *liquid biopsy-only mutations* affecting the different genes (*ATM*, *ATR*, *BRCA1*, *BRCA2*, *CHEK2*, *TP53* or other genes).

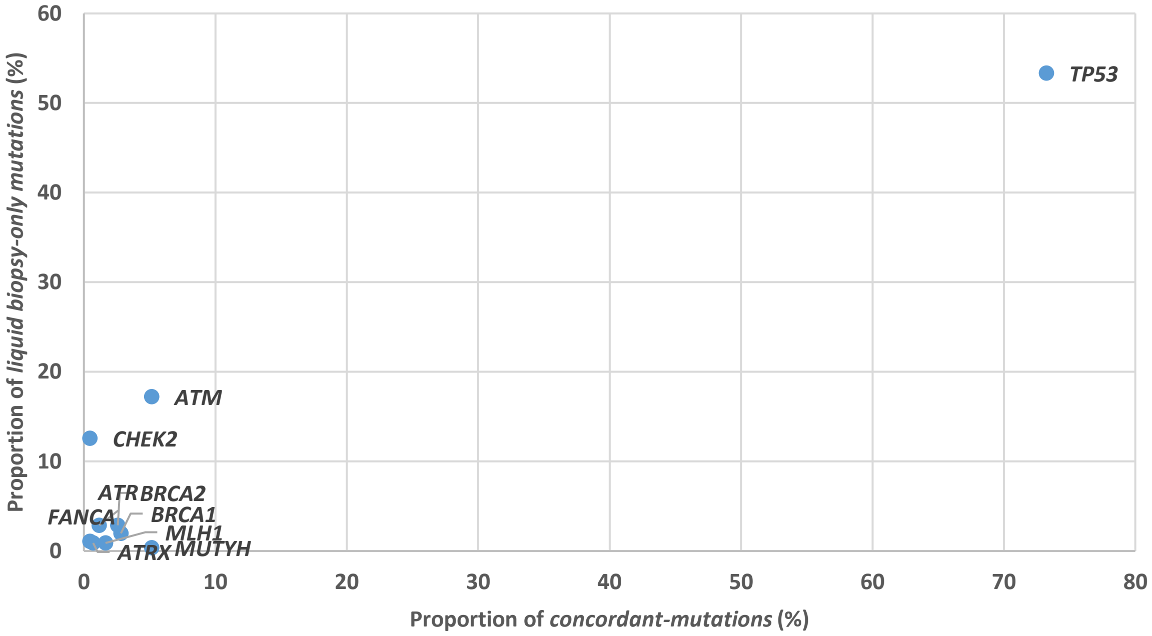

Supplementary Figure S5

Scatter plot representing the proportion of *liquid biopsy-only mutations* vs *concordant-mutations* occurring in the ten most mutated genes.

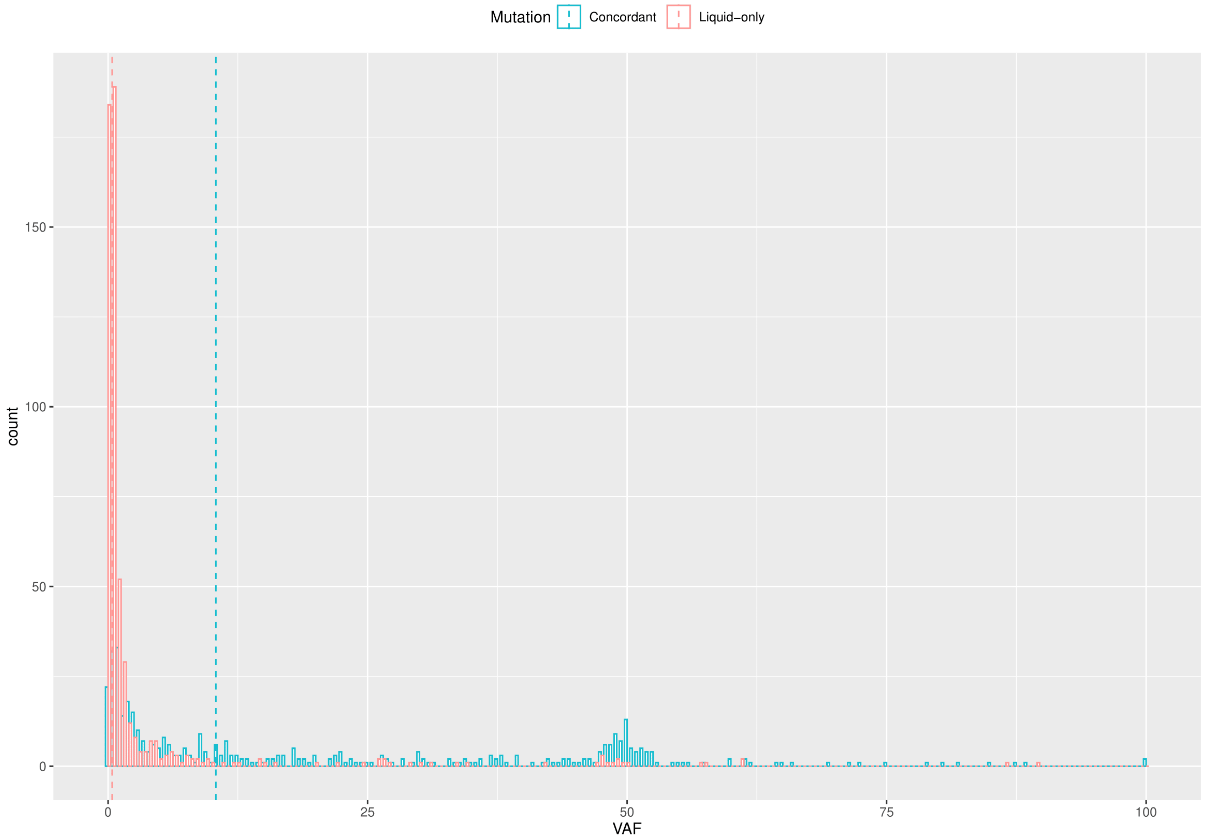

Supplementary Figure S6

Histogram representing the distribution of the VAF for *liquid biopsy-only mutations* and *concordant-mutations* across *TP53* and DDR genes.

Characteristics	Parameter	Mutation occurring in DNA Damage Response (DDR) gene or in *TP53*			
		DDR gene		*TP53*	
		Likely *liquid biopsy-only mutation*	Likely *concordant mutation*	Likely *liquid biopsy-only mutation*	Likely *concordant mutation*
Liquid biopsy	Variant allelic frequency	<1.0%	>10.0%	<1.3%	≥1.3%
	Hotspot amino acid	ATM p.Gly2891<br>ATM p.Arg3008	-	TP53 p.Thr125<br>TP53 p.Cys176<br>TP53 p.Ile195<br>TP53 p.Met237<br>TP53 p.Arg273<br>TP53 p.Arg280	-
	Variant type	Splice site	Missense	Splice site	Frameshift<br>Non sense
	Tumor fraction	Low	High	Low	High
Patient	Median age (IQR)	Older [64 (56 - 71)]	Younger [58 (48 - 66)]	Older [64 (56 - 71)]	Younger [58 (48 - 66)]
	Prior treatment lines	≥2 lines	<2 lines	≥2 lines	<2 lines
	Tumor type	-	Colorectal cancer	-	Colorectal cancer

Supplementary Figure S7

Summary of characteristics and parameters to classify mutations occurring in DDR genes and *TP53*
